# Correction: Health, Health Inequality, and Cost Impacts of Annual Increases in Tobacco Tax: Multistate Life Table Modeling in New Zealand

**DOI:** 10.1371/journal.pmed.1002211

**Published:** 2016-12-22

**Authors:** Tony Blakely, Linda J. Cobiac, Christine L. Cleghorn, Amber L. Pearson, Frederieke S. van der Deen, Giorgi Kvizhinadze, Nhung Nghiem, Melissa McLeod, Nick Wilson

The authors identified an error in model coding used to produce results in the original article. This involved a swapping of a + and − sign for females within the model, which flowed out to many of the results. Therefore, many numbers have now slightly changed in the corrected versions of the main manuscript, Figs 1–5, S1 Fig and the extra results in S2 Text.

**Fig 1:**
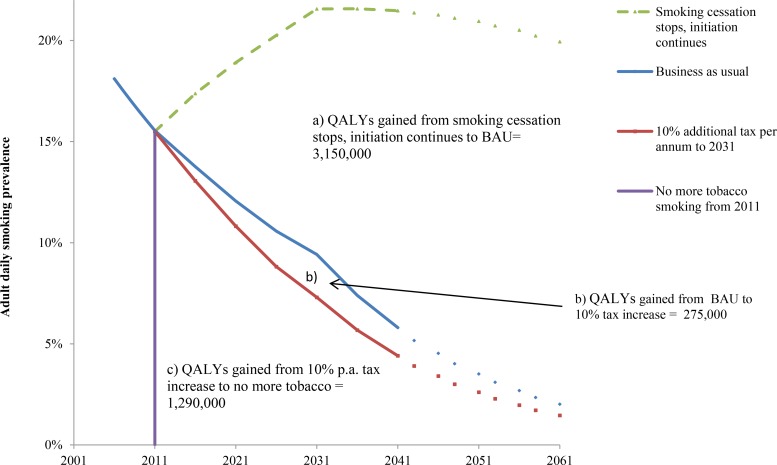
Future smoking prevalence in the New Zealand 2011 population by scenario and QALY gains between scenarios. QALYs gained for areas between the curves A, B, and C are undiscounted. QALYs discounted at 3% are (A) 655,000, (B) 58,000, and (C) 391,000. “Smoking cessation stops, initiation continues” = scenario of no further net smoking cessation among those already smoking in 2011, ongoing initiation of people aged up to 20 y of age in 2011. The prevalence therefore increases up to 2031 in this closed cohort (due to new smokers outnumbering differential deaths by smoking status), then declines over time because of aging of the population and the higher mortality rate of smokers. BAU = scenario of net cessation and initiation rate trends observed between 2006 and 2013 censuses continuing into the future (tax effects removed), including differential mortality from smoking (i.e., additionally allowing for higher mortality of current (and ex-) smokers that will also decrease prevalence).

**Fig 2:**
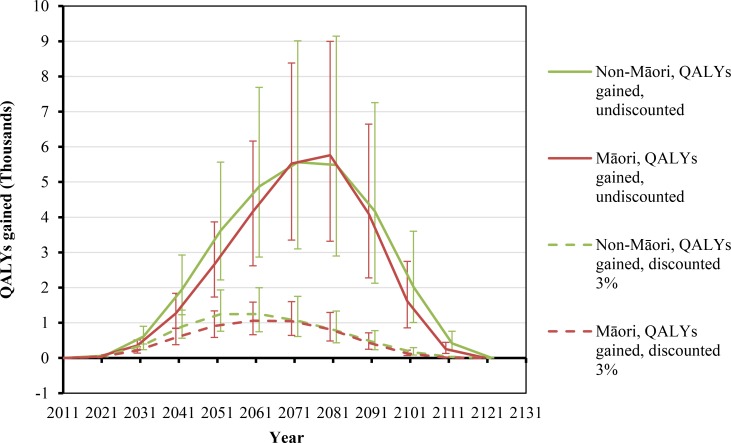
Projected QALYs gained by future year for 10% per annum tax increases from 2011 to 2031, by sex and ethnicity (in the 2011 cohort of the New Zealand population without replacement). Similar graphs by age group in 2011 are shown in S1 Fig.

**Fig 3:**
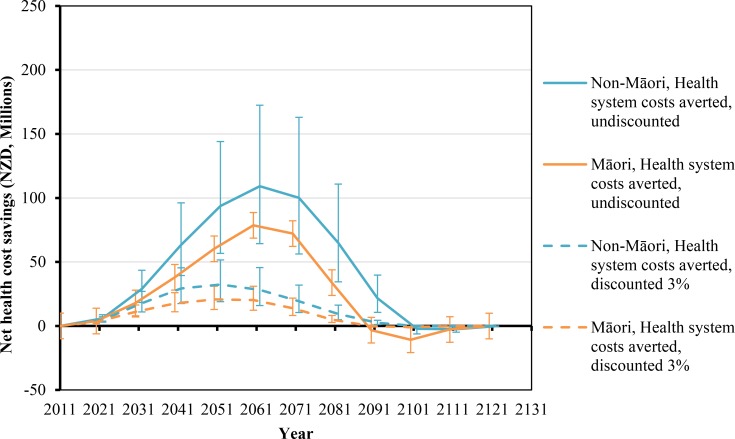
Projected net health system cost savings by future year for 10% per annum tax increases from 2011 to 2031, by sex and ethnicity (in the 2011 cohort of the New Zealand population without replacement). Similar graphs by age group in 2011 are shown in S1 Fig.

**Fig 4:**
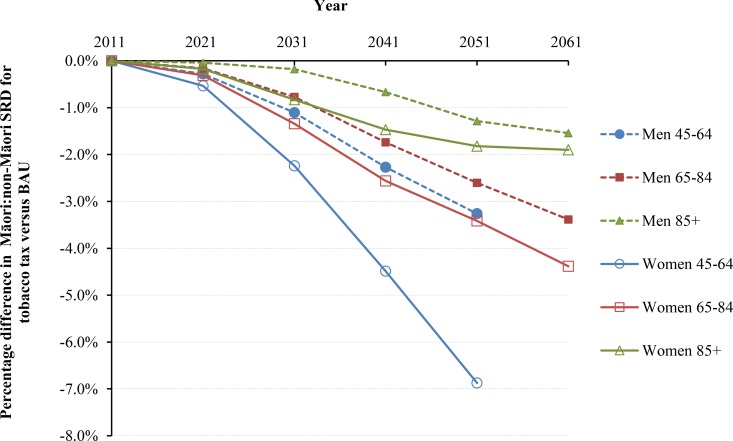
Projected percentage changes in ethnic inequalities in all-cause mortality rates for 10% increases in tobacco tax per annum from 2011 to 2031—Standardized rate differences (SRD) in mortality. Rates are standardized to the WHO world population.

**Fig 5:**
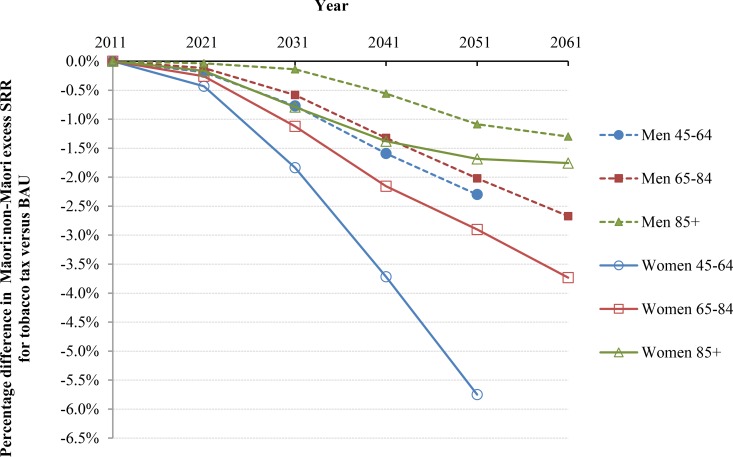
Projected percentage changes in ethnic inequalities in all-cause mortality rates for 10% increases in tobacco tax per annum from 2011 to 2031—Standardized rate ratios (SRR; percentage change in “excess” SRR or SRR-1). Rates are standardized to the WHO world population.

In addition to providing new versions of the main and S1 Fig, specific data have also been amended in the following files:

- S1 Manuscript Table 2: *Sex and age groups combined*; all *Women* data; *Per capita (QALYs/1*,*000 people and $)*
- S1 Manuscript Table 3: all data
- S2 Text S6 Table: all data
- S2 Text S7 Table: all data
- S2 Text S8 Table: all data
- S2 Text S9 Table: all data
- S2 Text S11 Table: *Sex and age groups combined*; all *Women* data; *Per capita (QALYs/1*,*000 people and $)*

Corrected versions of Figs 1–5, S1 Fig, S2 Text, and the manuscript have been provided below.

All qualitative conclusions in the text and the patterns in the graphs remain unchanged from the former version. That is the modeled tobacco tax interventions remain effective at generating large population health gains, reducing ethnic inequalities in health, and generating large cost savings to the health system.

## Supporting Information

S1 Manuscript Corrected manuscript. (DOCX)

S1 Fig Projected QALYs gained (thousands) and net health system costs saved (millions) by year for 10% per annum tax increase to 2031, by age cohort in 2011. (TIF)

S2 Text Corrected model, supplementary results, DISMOD II example, validation, epidemiological inputs, and health system costs (DOC)

## References

[1] Blakely T, Cobiac LJ, Cleghorn CL, Pearson AL, van der Deen FS, Kvizhinadze G, et al (2015) Health, Health Inequality, and Cost Impacts of Annual Increases in Tobacco Tax: Multistate Life Table Modeling in New Zealand. PLoS Med 12(7): e1001856 doi: 10.1371/journal.pmed.1001856 26218517 10.1371/journal.pmed.1001856 PMC4517929

