# Supplementary figures and images for: Health, Health Inequality, and Cost Impacts of Annual Increases in Tobacco Tax: Multistate Life Table Modeling in New Zealand
Source: PLoS Med. 2015 Jul 28;12(7):e1001856. doi: 10.1371/journal.pmed.1001856 (PMC4517929; doi:10.1371/journal.pmed.1001856)

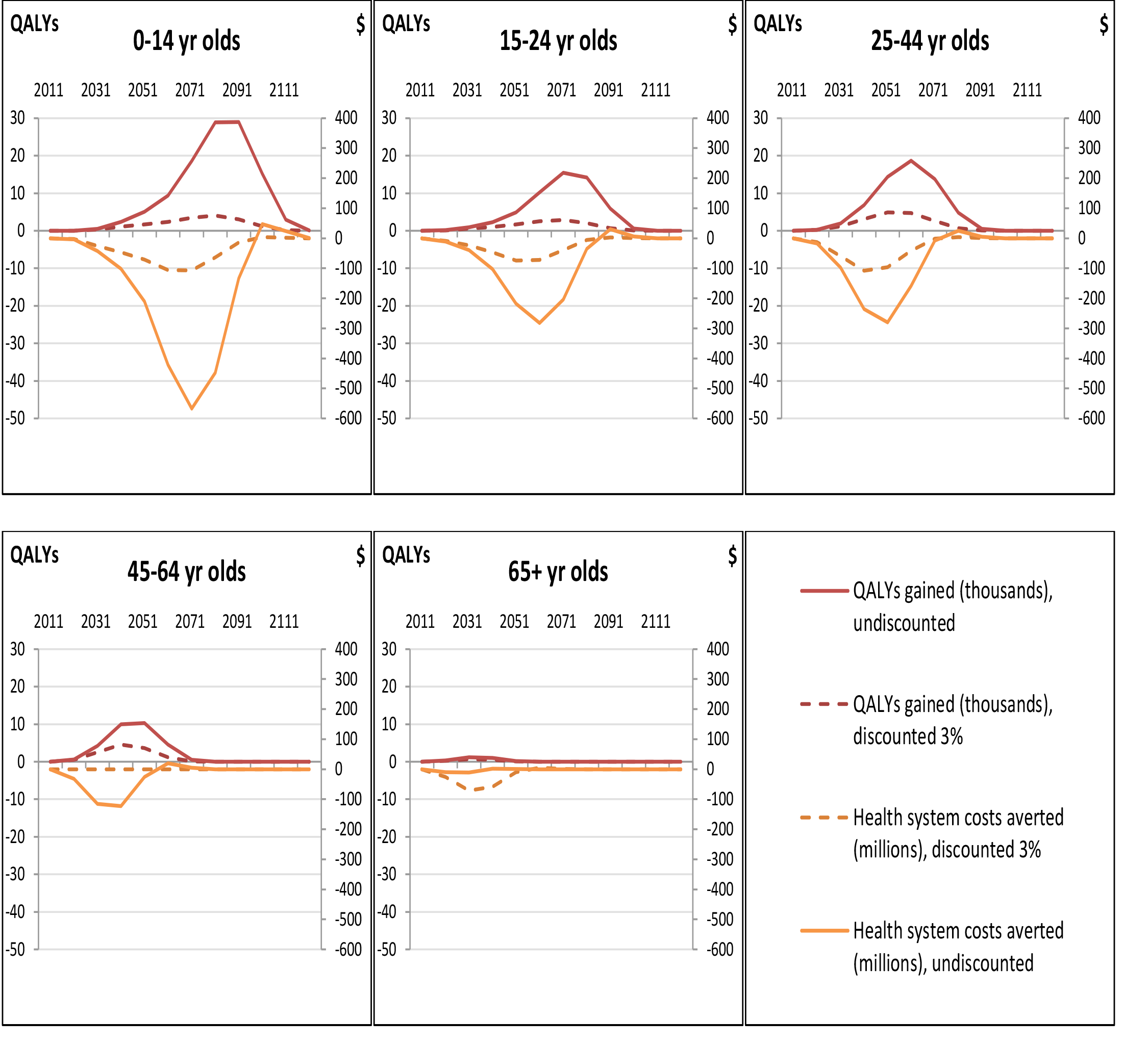

Supplement: S1 Fig — * Same as Fig S4 in S2 Text. (TIF) [file pmed.1001856.s001.tif]
