# Supplementary material for: Health, Health Inequality, and Cost Impacts of Annual Increases in Tobacco Tax: Multistate Life Table Modeling in New Zealand
Source: PLoS Med. 2015 Jul 28;12(7):e1001856. doi: 10.1371/journal.pmed.1001856 (PMC4517929; doi:10.1371/journal.pmed.1001856)
Supplement: S1 Table — (DOCX) [file pmed.1001856.s002.docx]

**S1 Table: QALYs gained by disease from a 10% per annum increase in tobacco tax (from 2011 to 2031), among the New Zealand population alive in 2011***

|  | **‘Best’ model†** | | | **Alternative model using CPS II relative risks for CHD and stroke‡** | | |
| --- | --- | --- | --- | --- | --- | --- |
|  | **QALYs gained** | **QALYs attributable to disease** | **% attributable** | **QALYs gained** | **QALYs attributable to disease** | **% attributable** |
| All disease included | 257,000 |  |  | 277,000 |  |  |
| *Cause deleted* |  |  |  |  |  |  |
| CHD | 234,000 | 23,500 | 9.2% | 233,000 | 44,200 | 15.9% |
| Stroke | 238,000 | 18,900 | 7.4% | 259,000 | 18,100 | 6.5% |
| COPD | 122,000 | 135,000 | 52.6% | 142,000 | 135,000 | 48.9% |
| Lung cancer | 190,000 | 66,900 | 26.0% | 210,000 | 67,000 | 24.2% |
| Mouth and oropharyngeal cancer | 255,000 | 2,260 | 0.9% | 275,000 | 2,260 | 0.8% |
| Esophageal cancer | 255,000 | 2,320 | 0.9% | 275,000 | 2,320 | 0.8% |
| Pancreatic cancer | 256,000 | 1,440 | 0.6% | 276,000 | 1,440 | 0.5% |
| Bladder cancer | 254,000 | 3,420 | 1.3% | 274,000 | 3,420 | 1.2% |
| Kidney cancer | 257,000 | 631 | 0.2% | 276,000 | 631 | 0.2% |
| Stomach cancer | 256,000 | 1,610 | 0.6% | 275,000 | 1,610 | 0.6% |
| LRTI | 256,000 | 1,240 | 0.5% | 276,000 | 1,240 | 0.4% |
| Liver cancer | 255,000 | 2,310 | 0.9% | 275,000 | 2,310 | 0.8% |
| Cervical cancer | 257,000 | 388 | 0.2% | 277,000 | 389 | 0.1% |
| Endometrial cancer | 258,000 | -447 | -0.2% | 277,000 | -448 | -0.2% |
| Melanoma | 259,000 | -2,080 | -0.8% | 279,000 | -2,080 | -0.7% |
| Thyroid cancer | 257,000 | -80 | 0.0% | 277,000 | -80 | 0.0% |

†10% per annum tax increase to 2031, undiscounted
‡ Alternate relative risks were used for CHD and stroke, from New Zealand specific estimates in the ‘Best’ model (RR for CHD 1.6 for males and 1.7 for females, and for stroke 2.5 and 2.2) [[1](#_ENREF_1), [2](#_ENREF_2)] to Cancer Prevention Study (CPS II; RR for CHD 35-64/65+ years 2.6/1.5 for males and 3.2/1.7 for females, and for stroke 2.4/1.5 and 3.8/1.6) [[3](#_ENREF_3)] in the ‘alternative model’.

*Same as Table S6 in S2 Text.

**References**

1. Hunt D. Mortality from smoking in New Zealand. Dunedin: University of Otago; 2003.

2. Hunt D, Blakely T, Woodward A, Wilson N. The smoking-mortality association varies over time and by ethnicity in New Zealand. Int J Epidemiol. 2005;34(5):1020-1028.

3. Thun M, Apicella L, Henley S. Smoking vs other risk factors as the cause of smoking-attributable deaths: confounding in the courtroom. JAMA. 2000;284(6):706-712.
