# Supplementary material for: Health, Health Inequality, and Cost Impacts of Annual Increases in Tobacco Tax: Multistate Life Table Modeling in New Zealand
Source: PLoS Med. 2015 Jul 28;12(7):e1001856. doi: 10.1371/journal.pmed.1001856 (PMC4517929; doi:10.1371/journal.pmed.1001856)
Supplement: S2 Table — (DOCX) [file pmed.1001856.s003.docx]

S2 Table: Scenario analyses about QALY and life year gains and health system cost savings for tobacco tax compared to BAU*

| **Scenario**† | **QALYs gained** | **Cost savings (millions)** |
| --- | --- | --- |
| ‘Best’ model‡ | 260,000 (155,000 to 419,000; ratio upper to lower 2.71) | $3,770 ($2200 to $5940; ratio upper to lower 2.70) |
| *Alternative taxes* |  |  |
| 20% per annum tax increase to 2031 | 499,000 | $7,130 |
| 10% per annum increase to 2021 | 257,000 | $2,130 |
| 10% per annum increase to 2041 | 314,000 | $2,500 |
| *Varying price elasticity by ethnicity* |  |  |
| Price elasticities the same for Māori and non-Māori | 241,000 (88,200 Māori, 153,000 non-Māori) | $3560 ( $1040 Māori, $2520 non-Māori) |
| *Discount rate* |  |  |
| 3% per annum | 60,400 | $1,190 |
| 6% per annum | 19,000 | $330 |
| *Varying uncertainty* |  |  |
| Reduced uncertainty (2.5%, 5% and 10% SDs instead of 5%, 10% and 20% SDs; see Methods) | 257,000 (198,000 to 327,000) | $3730 ($2820 to $4790) |
| Increased uncertainty (10%, 20% and 40% SDs instead of 5%, 10% and 20% SDs; see Methods) | 267,000 (91,000 to 600,000) | $3770 (1290 to 8210) |
| Doubling confidence interval about all relative risks used in model. | 257,000 (150,000 to 411,000) | $3690 (2070 to 6130) |
| *Varying CHD and Stroke RR to CPS II*** |  |  |
| All sexes, ages and ethnic groups combined | 277,000 | $3,880 |
| *Disease trend continue post 2026* |  |  |
| All sexes, ages and ethnic groups combined | 338,000 | $1,280 |
| **Life years gained per capita (i.e. no morbidity reduction benefits included in model)** | | |
| *Morbidity set to zero, 3% discount rate* |  |  |
| All sexes, ages and ethnic groups combined | 50,300 (30,500 to 77,500) | na |
| *Morbidity set to zero, 0% discount rate* |  |  |
| All sexes, ages and ethnic groups combined | 246,000 (143,000 to 400,000) | na |
| -          Māori, 0-14 yrs | 56,300 (32,520 to 93,300) | na |
| -          Māori, 15-24 yrs | 22,700 (13,600 to 35,800) | na |
| -          Māori, 25-44 yrs | 21,600 (13,700 to 32,500) | na |
| -          Māori, 45-64 yrs | 8,800 (5,600 to 13,400) | na |
| -          Māori, 65+ yrs | 510 (320 to 780) | na |
| -          Non-Māori, 0-14 yrs | 44,400 (23,600 to 78,300) | na |
| -          Non-Māori, 15-24 yrs | 27,500 (14,700 to 47,900) | na |
| -          Non-Māori, 25-44 yrs | 38,500 (22,500 to 62,000) | na |
| -          Non-Māori, 45-64 yrs | 23,100 (14,000 to 36,000) | na |
| -          Non-Māori, 65+ yrs | 2,410 (1,500 to 3,710) | na |

† Alternative taxes and varying price elasticity by ethnicity scenarios are undiscounted
‡10% per annum tax increase to 2031, undiscounted, price elasticities 20% higher for Māori

* Same as Table S7 in S2 Text.

** As in S1 Table (and footnotes).
