# Supplementary material for: Health, Health Inequality, and Cost Impacts of Annual Increases in Tobacco Tax: Multistate Life Table Modeling in New Zealand
Source: PLoS Med. 2015 Jul 28;12(7):e1001856. doi: 10.1371/journal.pmed.1001856 (PMC4517929; doi:10.1371/journal.pmed.1001856)
Supplement: S3 Table — (DOCX) [file pmed.1001856.s004.docx]

S3 Table: QALYs gained and health system costs averted, with uncertainty, from a 10% per annum increase in tobacco tax (from 2011 to 2031), among the New Zealand population alive in 2011 (the same as Table 2 in the main manuscript, but showing uncertainty)*

|  | **Non-Māori** | | **Māori** | | | **Ethnic groupings combined** | |
| --- | --- | --- | --- | --- | --- | --- | --- |
| **Sex and age (in 2011)** | **QALYs** | **Cost savings (millions)** | **QALYs** | **QALYs – equity†** | **Cost savings (millions)** | **QALYs** | **Net cost savings (millions) ‡** |
| Sex and age groups combined | 156,000 (90,300 to 254,000) | $2550 (1460 to 4060) | 105,000 (64,100 to 163,000) | 156,000 (91,300 to 247,000) | $1220 ($738 to $1880) | 260,000 (155,000 to 419,000) | $3770 ($2200 to $5940) |
| *Men* |  |  |  |  |  |  |  |
| 0-14 year olds | 29,600 (16,600 to 49,500) | $604 (342 to 969) | 29,100 (17,800 to 45,500) | 44,700 (26,000 to 71,100) | $510 (309 to 776) | 58,700 (34,500 to 94,700) | $1110 (652 to 1740) |
| 15-24 year olds | 18,000 (9,860 to 30,600) | $321 (174 to 527) | 11,900 (7,110 to 18,800) | 18,400 (10,500 to 29,900) | $181 (106 to 282) | 29,900 (17,100 to 49,100) | $502 (281 to 808) |
| 25-44 year olds | 20,000 (11,500 to 32,300) | $285 (157 to 461) | 7,840 (4,800 to 12,200) | 12,500 (7,440 to 19,800) | $93 (54 to 146) | 27,800 (16,400 to 44,300) | $378 (212 to 605) |
| 45-64 year olds | 7,100 (4,160 to 11,400) | $82 (45 to 133) | 1,620 (970 to 2,550) | 2,870 (1,670 to 4,630) | $15 (8.6 to 25) | 8,750 (5,150 to 14,000) | $97 (54 to 158) |
| 65+ year olds | 320 (190 to 510) | $3.0 (1.7 to 4.8) | 30 (20 to 50) | 60 (40 to 110) | $0.4 (0.2 to 0.6) | 350 (210 to 550) | $3.3 (1.9 to 5.4) |
| All ages | 75,100 (42,300 to 124,000) | $1290 (724 to 2090) | 50,500 (30,700 to 79,000) | 78,600 (45,800 to 125,000) | $799 (477 to 1230) | 125,600 (73,600 to 203,000) | $2090 (1200 to 3330) |
| *Women* |  |  |  |  |  |  |  |
| 0-14 year olds | 26,100 (14,400 to 44,900) | $493 (275 to 812) | 27,000 (15,500 to 45,300) | 38,000 (20,700 to 63,500) | $252 (146 to 407) | 53,200 (30,100 to 89,400) | $745 (422 to 1210) |
| 15-24 year olds | 14,900 (8,260 to 25,200) | $271 (150 to 444) | 9,810 (6,090 to 15,100) | 13,700 (8,070 to 21,400) | $83 (50 to 130) | 24,700 (14,400 to 40,300) | $354 (202 to 569) |
| 25-44 year olds | 22,100 (13,300 to 34,700) | $324 (189 to 512) | 11,300 (7,300 to 16,900) | 16,500 (10,200 to 24,900) | $67 (41 to 105) | 33,400 (20,700 to 51,400) | $391 (229 to 612) |
| 45-64 year olds | 15,900 (9,810 to 24,500) | $155 (91 to 245) | 5,530 (3,530 to 8,350) | 8,880 (5,490 to 13,580) | $21 (12 to 34) | 21,400 (13,400 to 32,800) | $176 (104 to 278) |
| 65+ year olds | 1,880 (1,180 to 2,880) | $13 (7.4 to 20) | 360 (220 to 550) | 640 (390 to 980) | $1.5 (0.8 to 2.3) | 2,200 (1,400 to 3,400) | $14 (8.3 to 22) |
| All ages | 80,900 (47,500 to 133,000) | $1250 (713 to 2040) | 54,000 (32,900 to 85,800) | 77,800 (45,000 to 123,000) | $425 (251 to 673) | 135,000 (80,200 to 217,000) | $1680 (963 to 2680) |
| Per capita (QALYs /1000 people & $) | 42 (24 to 68) | $683 (390 to 1090) | 155 (95 to 242) | 232 (135 to 367) | $1820 (1090 to 2790) | 59 (35 to 95) | $856 (499 to 1350) |

†Māori ‘QALYs equity’ are calculated using non-Māori background mortality and morbidity rates, so as not to ‘penalize’ Māori in terms of future health gain due to poorer current background mortality and morbidity.

‡Includes both the cost offsets and intervention cost, the latter being the cost of a law (NZ$3.5 million, 95% UI $2.0 to $6.2 million [1]) to introduce tobacco taxes increases of 10% per annum to 2031, distributed pro-rata across all citizens alive in 2011.

*Same as Table S11 in S2 Text.

**Reference**

1. Wilson N, Nghiem N, Foster R, Cobiac L, Blakely T. Estimating the cost of new public health legislation. Bull World Health Organ. 2012;90: 532-539.
