# Supplementary material for: Health, Health Inequality, and Cost Impacts of Annual Increases in Tobacco Tax: Multistate Life Table Modeling in New Zealand
Source: PLoS Med. 2015 Jul 28;12(7):e1001856. doi: 10.1371/journal.pmed.1001856 (PMC4517929; doi:10.1371/journal.pmed.1001856)
Supplement: S1 Text — (DOCX) [file pmed.1001856.s005.docx]

# S1 Text: Literature reviews’ methods and results

## Studies of the health and cost impacts of tobacco tax (cost-effectiveness and cost-benefit analyses)

***Search strategy:*** We searched PubMed on 22 June 2014 for original articles on the impact of tobacco tax on health (or disease) and future health system costs (usually averted or saved), as well as actual cost-effectiveness or cost-benefit studies. The search terms (separately and in combination) were: “tobacco”, “price or tax”, and “QALY or DALY or HALY or life year” (for the health metric). We also did a manual search based on bibliographies of papers we found.

There were 11 studies identified in the peer-reviewed literature which estimated the health gain from a tobacco tax, and also the related costs (intervention costs and/or related health care costs; S1 Text Table 1). Most were for developed countries (6/11), four were for developing countries, and one was a mix. All reported overall health gains and the tax increases were reported as either very cost-effective or were cost-saving. Methodologies varied e.g., use of different discount rates and different considerations around which costs were included. Some studies considered cost offsets from tobacco-related diseases only, and only two studies considered these as well as the health costs associated with extra life lived as a result of the intervention. None of the studies considered multiple tobacco tax increases (even though these are increasingly used internationally e.g., in New Zealand (every year from 2010 to 2015 and planned to 2016 [1]), Australia (four increases legislated from 2013 [2]), and Germany (five increases in 2001-2006 [3]).

Three studies considered differences in health gain by sex and for all these the gain for men was greater. One study in the Netherlands considered socioeconomic status (SES) [4]. It found that in terms of QALYs gained per person this was greatest in the lowest (least educated) SES group at 1.8 per 1000 people (vs 1.6 QALYs in the highest SES group). But QALY gain per smoker was actually higher in the highest SES group (6.9 vs 5.9 QALYs per 1000 people). This study also found that discounted costs were greater with tobacco prevention. The underlying model, the RIVM Chronic Disease Model (CDM), accrues costs per prevalent case modelled out to the future, and it has been shown elsewhere with this model that obesity prevention and tobacco control are estimated to result in increased health sector costs [5]. (See the text relating to S1 Text Table 3 below for extra comment on this RIVM-CDM model.) As a result, the overall cost-effectiveness was most favourable for the highest SES group at 4500 Euros per QALY gained (vs 6100 in the lowest SES group). In a further age-stratified analysis (published as a subsequent letter [6]) the extra QALY gain was largest in the 41-60 year age group of smokers (7.3 per 1000 in the highest SES group vs 6.5 in the lowest SES group).

Another study we identified (and which is not included in the table since it lacked cost data) was a modeling study for a tobacco tax increase in California [7]. It reported that the short-term (1 year) and long-term (75 years) health gain was greater for the Latino population relative to the general population in California (eg, for the long-term: 4606 vs 3658 QALYs gained/million smokers respectively, which is 1.26 times more). Although most results in scenario analyses favoured greater health gain from the tax increase for the Latino population of smokers (per million smokers), there were three out of ten scenarios that did not.

S1 Text Table 1: Summary of 11 published studies that quantified health gain from tobacco tax (eg, in QALYs or HALYs gained, or DALYs averted) and which also considered costs (intervention and/or health care costs) as well

| **Study** | **Tobacco tax intervention** | **Main findings and selected notes** | **Cost-offsets, Costs from extra life?** | **Ethnic/ SES inequal-ities aspect?** | **Gender inequal-ities aspect?** |
| --- | --- | --- | --- | --- | --- |
| **Developed countries (or a mix of developed and developing countries)** | | | | | |
| Over et al 2014[4] | Total price rise of 5% | This study of adults in the Netherlands reported an overall ICER of 5,000 Euros per QALY gained. The SES patterning was complex. In terms of QALYs gained per 1000 persons this was greatest in the lowest SES group at 1.8 (vs 1.6 QALYs in the highest SES group). But QALY gain per 1000 *smokers* was higher in the highest SES group (6.9 vs 5.9 QALYs) – especially in the 41-60 year age group of smokers (7.3 in the highest SES group vs 6.5 in the lowest SES group) [4]. As a result the overall cost-effectiveness was higher for the highest SES group at 4500 Euros per QALY gained (vs 6100 in the lowest SES group). The discount rate (DR) was 4% for costs and 1.5% for health gain and a 75 year time horizon was used. Intervention cost for tax was zero. Various scenario analyses were included and the ICER became “cost saving” when considering just the programme costs and the costs of smoking-related diseases (for all SES groups). This study also considered other tobacco control interventions. See the text and S1 Text Table 3 for more comment on this study. | Both considered | Yes for SES (and pro equity in terms of QALYs gained per 1000 population) | Higher deaths averted for men vs women |
| Vos et al 2010 (ACE Prevention Australia [8]) | One-off 30% (scenarios: 10%, 15%, 50%, 60%). | This study of the entire Australian population found that for a 30% increase, with annual indexation to inflation, there would be 270,000 DALYs averted (13.6 per 1000 population) [using the June 2003 Australian population of 19.88 million]. There would be $A 18 million in lifetime intervention costs spent, and cost-offsets of $A -690 million (so net cost-saving). But this study did not consider health costs from extra years of life lived. Strength of evidence for taxation benefits was considered “likely”. The second filter was “political will”. | Cost offsets only | Not detailed | Not detailed |
| Reed 2010 [9] | 5% increase in tobacco prices | This study was a cost-benefit analysis (CBA) in the UK (for ASH) using a societal perspective. It assumed a price elasticity for smoking prevalence (PE) = -0.35. It estimated that 190,000 smokers would quit with UKP10.2 billion [B] in cost savings (of which UKP5.74B was value of extra life years, UKP1.97B NHS savings, UKP1.36B reduced absenteeism). | Cost offsets only | Not detailed | Not detailed |
| Van Baal et al 2007 [10] | Price increase of 10% | This study reported that the price increase was cost-effective at 2500 Euros per QALY gained for the Netherlands (when considering additional medical costs from the life years gained). The study used a DR=4% for costs, 1.5% for health gain, and the model was the RIVM CDM. The intervention was cost saving if short time horizon (25 years) was considered. With no discounting and 100 year time horizon, ICER = 9700 Euros per QALY. See S1 Text Table 3 for more on this study. | Both considered | Not detailed | Not detailed |
| Ahmad 2005 [11] | 20% price increase | This study was for the population of California, USA. QALYs were gained and there were cumulative medical savings (after 75 years). But the model did not consider any intervention costs, did not use discounting, and it is unclear if costs from extra life were considered. Of note was the idea that smuggling might erode some of the benefits: “research suggests that the impact of smuggling is not large, and smuggling may reduce but will not eliminate the economic benefits of excise tax increases.” | Cost offsets & unclear as to extra life costs | Not detailed | Not detailed |
| Fishman et al 2005 [12] | $1 per pack tax (albeit combined with a media campaign) | This US study considered both a tax increase and an anti-smoking mass media campaign. This package resulted in a year of life saved for an average of $US 622 ($559–$669) when considering the direct health costs (DR=3%). At DR=7% the figures were: $2,353 ($2,099–$2,376). Other calculations indicated net cost savings (eg, when tax revenue was included in the calculations). Costs of harm to neonates from smoking and to children from secondhand smoke were considered. | Cost offsets only | Not detailed | Not detailed |
| Ranson et al 2002 [13] | One-off increase in real price of 10% | This study reported that this price increase would “cost 3-70 US dollars per DALY saved in low-income and middle-income regions”. Also, “In high-income countries, price increases were found to have a cost-effectiveness of 83-2771 US dollars per DALY.” Discount rates (DR) ranging from 3% to 10% (both health gain and costs) were used. This study also compares the results for tax with a league table of other (non-tax) CEAs in tobacco control. | Neither considered | Not detailed | Not detailed |
| **Developing countries** | | | | | |
| Ortegon et al 2012 [14] | A one-off tax increase of 60% (also 40%) | This study calculated an ICER of $I 448 per DALY averted in the sub-Saharan Africa region and $I 87 per DALY averted in the South East Asian region. It considered intervention costs and allowed for comparisons with a large range of other interventions (123 preventive and treatment interventions in total). | Neither | Not detailed | Not detailed |
| Salomon et al 2012 [15] | One-off from 60% of the pack price to 80% | This study was for Mexico and calculated ICERs were: $I 103 per DALY averted (3% discount rate, no age weighting) or $137 (3% with age weighting). (Detailed in the Appendix of the study). This analysis also combined tobacco tax with other interventions (NRT, and clean air laws). A total of 101 interventions were considered. | Neither | Not detailed | Not detailed |
| Higashi et al 2011 [16] | Increase from 55% of pack price to 65%,75%,85% | This study was based in Vietnam and tax increases were found to be highly cost-effective in averting DALYs (but less so than graphic warnings on packs). All interventions were cost-saving, and therefore dominant in cost-effectiveness terms (when considering cost-offsets for just the 3 main disease groups). | Only selected cost-offsets | Not detailed | Much higher health gain for men vs women |
| Asaria et al 2007 [17] | Increase in real price enough to reduce prevalence by 10% | This study was of low and middle income countries which found that tobacco tax generated health gain in terms of deaths averted. The intervention cost for the tax component per person was very low (range US$0.02 to US$0.17). Nevertheless, the study didn’t consider health care costs (and did not calculated a CER or ICER from the intervention cost). | Neither | Not detailed | Higher deaths averted for men vs women |

## Reviews of the health-related equity/inequality impacts of tobacco tax

***Search strategy:*** To provide context around the equity impacts of tobacco taxation, we searched PubMed on 22 June 2014 for systematic reviews on tobacco taxes and equity (for the period: 1 January 2005 to 22 June 2014). The search terms (separately and in combination) were: “systematic review”, “tobacco or smoking”, “price or tax”, and “equity or inequal*”. We also did a manual search based on bibliographies of papers we found.

This section considers whether the health gains (not the cost) from a tobacco tax preferentially benefit social groups with poorer initial health. That is, does tobacco tax cause a greater health gain among socially disadvantaged groups? Given the relatively large literature on the topic we focused on just ***systematic reviews*** of inequality (socioeconomic, racial/ethnicity) impacts of tobacco tax or prices (as per the search strategy detailed above).

We identified six relevant systematic reviews published since 2005 (as detailed in S1Text Table 2 below). The two most recent ones indicated that tobacco price/tax increases tended to have a positive impact on equity (i.e., reduction in inequalities in smoking prevalence by socioeconomic status) for both adults [18] and youth [19]. The four other reviews also reported this same general pattern – albeit more tentatively. The focus tended to be on low vs high SES (using education or income), with relatively few studies considering inequalities based on ethnicity, age, or sex. Nevertheless, in terms of ethnic inequalities, one included study was a US one that found that “Hispanic smokers and non-Hispanic black smokers were more likely than white smokers to reduce or quit smoking in response to a price increase. This pattern was consistent for all age groups” [20]. For example, the decrease in smoking (for the 18-24 year age group) was: 27% for Hispanics, 10% for non-Hispanic blacks, and 1% for non-Hispanic whites (based on Figure 1 in the journal publication).

None of these systematic reviews attempted a quantitative synthesis of the results from the different studies (e.g., in terms of price elasticity of demand or reduction in consumption). Also they all focused on smoking prevalence/tobacco consumption and not differences in health status (i.e., reduced disease rates, mortality rates or QALYs gained from tobacco interventions).

As noted earlier in this Appendix, there is also some evidence from studies that used health metrics that identified some pro-equity benefits from a tobacco tax in terms of socioeconomic groups in the Netherlands [4] and for the Latino population in California [7].

S1 Text Table 2: Summary of systematic reviews examining the inequality impacts of tobacco taxation (published since 2005)

| **Review** | **Main findings** | **Comment / extra details** |
| --- | --- | --- |
| Brown et al 2014 [18] (adults) | This review concluded that: “Fourteen studies demonstrated a positive [socioeconomic] equity impact, six studies were neutral, one mixed and two unclear. Four studies showed a negative equity impact (including studies of HIV-positive adults (Peretti-Watel et al., 2009) and pregnant women (Ringel and Evans, 2001).” The main conclusion was that: “Few studies have assessed the [socioeconomic] equity impact of tobacco control policy/interventions. Price/tax increases had the most consistent positive equity impact.” | This seems the most up-to-date systematic review on this topic (for adults). It included 27 studies on price/tax included, which spanned the years 1998 to 2012. The review also reported that: “Low SES smokers appeared more responsive to price/tax increases in terms of larger price elasticities compared with high SES smokers. However, larger price elasticities among lower SES adults might be capturing short-term effects which do not translate into sustained quitting. In addition, cross-border sales or smuggling were not accounted for in most econometric studies. This omission might have biased the results: lower SES adults might be more likely to mitigate the effects of price/tax increases by switching to cheaper brands or buying illicit tobacco. However, a recent European study (Nagelhout et al., 2013a,b) showed that cross-border cigarette purchasing was more often reported by high SES smokers.” This review didn’t mention ethnic inequalities specifically. The studies also just focused on smoking prevalence and consumption – and not health outcomes. |
| Brown et al 2014 [19] (youth) | This review concluded that: “Very few studies have assessed the equity impact of tobacco control interventions/policies on young people. Price/tax increases had the most consistent positive [socioeconomic] equity impact.” | This seems the most up-to-date systematic review on this topic for youth smoking. It included 7 studies on price/tax. “Four studies demonstrated a positive equity impact, one study was neutral and two negative.” This review did not report ethnic inequalities specifically. There was no quantitative synthesis of the results. The results of this systematic review contrast with those of another systematic review of individual-level smoking cessation interventions (none were tax/price related) to reduce smoking in adults (delivered in European countries) [19]. It found that “the distribution of equity effects on quitting was 10 neutral, 18 negative and 1 unclear”. |
| Hill et al 2014 [21] | This review concluded that: “We found strong evidence that increases in tobacco price have a pro-equity effect on socioeconomic disparities in smoking. Evidence on the equity impact of other interventions is inconclusive, with the exception of non-targeted smoking cessation programmes which have a negative equity impact due to higher quit rates among more advantaged smokers.” | This review involved data from 77 primary studies and seven reviews were synthesized via narrative review. |
| Chaloupka et al 2011 [22] (based on the IARC Report [23]) | This review was based on the work of an expert panel which assessed the effectiveness of price/tax policies in 2010. They concluded that there was “strong but not sufficient” evidence that lower income populations (in high income *countries*) are more responsive to price/tax increases than higher income groups. | In the full IARC Report,[23] the studies listed tended to show higher price elasticities for demand of tobacco in populations that had lower income or lower levels of education. But no quantitative synthesis (i.e. meta-analysis) was performed (possibly due to the large variation in study design). |
| Thomas et al 2008 [24] | This review concluded that: “Increasing the price of tobacco products may be more effective in reducing smoking among lower-income adults and those in manual occupations, although there was also some evidence to suggest that adults with higher levels of education may be more price-sensitive. Young people aged under 25 are also affected by price increases, with some evidence that boys and non-white young people may be more sensitive to price.” | The lead author of this review also contributed to the one directly below (Main et al, 2008). |
| Main et al 2008 [24] | This review reported that: “We found tentative evidence that the effect of increasing the unit price of tobacco products may vary between ethnic and socio-economic groups, and between males and females.” The conclusion was: “There is preliminary evidence that increases in the unit price of tobacco may have the potential to reduce smoking related health inequalities.” | This was a systematic review of systematic reviews in tobacco control. |

## Studies reporting the overall lifetime health system cost impacts of smoking and tobacco control interventions

***Search strategy:*** To provide context around the debate of whether or not smoking increases or decreases lifetime health costs, we searched PubMed on 15 July for relevant articles. The search was for the period: 1 January 2005 to 15 July 2014, and the search terms (in combination) were: “tobacco or smoking” and “costs” and “lifetime”. We also did a manual search based on bibliographies of papers we found.

There is some divergence in the literature over whether or not smoking results in overall increases in lifetime health costs (i.e., smoking-related illness may result in increased health costs in the short-term – but in the longer term smokers tend to have shorter lives and this can result in reductions in health costs). This section considers this issue, based on the results of the search strategy detailed above.

We identified one previous review on this topic that was published in 2001 [25]. It reported that smoking increased lifetime medical costs: “The overwhelming body of evidence in the literature asserts that smoking imposes costs on an annual basis, that it leads to increased medical costs over the life span, and that many of these costs are borne by employers”. But there have been more sophisticated modeling studies since this time so our literature search was just focused on relevant studies published since January 2005 and which also considered health costs for non-tobacco related diseases. Of the studies we identified (as detailed in the S1 text Table 3 below) most did report overall increased net lifetime health costs from reduced smoking (i.e., non-smokers and ex-smokers were more expensive in the long-term when also considering non-tobacco-related lifetime costs). But most of these studies used the same model: the RIVM Chronic Disease Model (CDM) for the Netherlands.

Two studies provided mixed results: one suggesting cost savings in the first two decades due to reduced smoking, but not thereafter (actually a study using the RIVM-CDM model – van Baal et al 2007 [10]). Another study found overall lifetime cost savings for women, but not men, a US study by Yang et al [26].

Only one study clearly reported overall net cost savings associated with reduced smoking – a study in Denmark (Rasmussen et al [27]).

There can be tremendous variations in the net health system cost depending on how diseases are costed, model structure assumptions, whether timing of costs (which may change with prevention) are included, and so on. Van Baal et al provides an analysis of the impacts of many of these aspects [28].

For the various studies in S1 Text Table 3 below there was certainly variation in the results according to such factors as the discount rate. For example, one of the RIVM-CDM model studies gave cost-saving results if the discount rate was at least 5.7%. Another study using the RIVM-CDM model which showed lower costs with smoking, actually used a zero discount rate (Kanters et al [29]).

More specific reasons why the RIVM-CDM model might tend to produce higher lifetime costs for ex-smokers and non-smokers (relative to smokers) are considered below:

- The RIVM-CDM model includes resthome costs within the health sector costing – which tends to increase costs for ex-smokers and non-smokers who live longer (than smokers). Even so, when these costs were excluded in a scenario analysis in one of the Dutch studies (to make them more internationally comparable), the main result of smokers costing less over their lifetimes was still obtained (Scenario 5 in van Baal et al 2008 [5]).
- Another consideration is if the timing of disease costs are partitioned i.e., the first year of diagnosis, the last year of life, and the period in-between (as we have done in our New Zealand modeling – see the main manuscript). The RIVM-CDM model studies (nor any other of the studies in S1Text Table 3 below) do not appear to have done such partitioning. The likely result of this is that in the “averaged prevalent” cases of disease among non-smokers and ex-smokers, the cumulative costs may have been artificially increased.
- The method used in the RIVM-CDM model may have involved some underestimation of the disease costs for smokers (relative to non-smokers and ex-smokers). That is, in in the relevant cost-of-illness study used, 8.9% of health costs were not allocated directly to illnesses and 8.6% of healthcare-related costs were for “unavailable diagnoses” [30]. Furthermore, this Dutch cost-of-illness study was for 2003, and since then there is likely to have been changes in healthcare expenditure patterns in the Netherlands (e.g., spending on cardiovascular disease has possibly been rising faster than other diseases which creates more potential for cost savings through smoking cessation [Personal Communication, Pieter van Baal with Tony Blakely, 2 September 2014]).

Regardless of these issues around healthcare costs, when expanding the perspective from the health sector and considering issues such as lost productivity costs from smoking, then many studies find that smoking generates higher total (health plus productivity) costs to society [9, 26, 27, 31-33].

S1 Text Table 3: Studies identified that quantify future lifetime and net health system costs from smoking and tobacco control interventions (published from 2005 on and which consider tobacco-related disease costs and also costs from extra life lived by non-smokers/ex-smokers)

| **Study** | **Overall impact of smoking / quitting on lifetime health costs** | **Selected additional details** |
| --- | --- | --- |
| **Studies suggesting smokers cost relatively less (health system costs)** | | |
| Over et al 2014 [4] | Reducing smoking (from various interventions) increased overall health costs | This study applied a health care perspective. Incremental costs of the modeled tax rise were negligible, given that a tobacco tax was already in place and only increased by a small amount (5%). Reimbursement costs consisted of the specific reimbursed costs for cessation therapy along with the cost of two consultations with a general practitioner (GP) for pharmacotherapy. Costs were estimated bottom-up by multiplying resource use with unit costs. Future costs were discounted at 4% (health gain by 1.5%). A computer simulation model (RIVM-CDM) that linked risk factors to the incidence and prevalence of 20 chronic diseases was used to estimate future health care costs by age, gender, and SES. The output of the RIVM-CDM consists of population health care costs for diseases both related and unrelated to smoking. The total incremental costs (sum of the intervention cost and incremental health care costs) for the tax rise scenario were 25, 29, 27 and 15 million euros for lowest, low, high and highest SES groups respectively. In this scenario the total incremental costs were equal to incremental health care costs as intervention costs were set at zero. For the reimbursement scenario the total incremental costs were 130, 170, 190 and 100 million euros for lowest, low, high and highest SES groups from which 38, 56, 61 and 43 million were incremental health care costs and the rest were intervention costs. See S1 Text Table 1 for other details. |
| Kanters et al 2013 [29] | Smokers had modestly lower lifetime and per life-year health costs | This study considered lifetime health costs (and revenue for insurers) for 20 years old in different groups in the Netherlands. It also used the RIVM-CDM model and used smoking and obesity as risk factors to simulate the prevalence of 22 related diseases. All other diseases were assumed to be unrelated to smoking or obesity in these analyses and were summed in one variable. The prevalence of unrelated diseases was simulated for this variable and was only dependent on the numbers of survivors in the cohorts. Lifetime health costs (Euros) were as follows: Healthy living 123,121; Smokers 106,435; Obese 118,120. Given that the remaining life expectancy for those groups were 64.4, 57.4 and 59.9 years respectively, it resulted in the per life year cost (on average) in Euros being: Healthy living: 1911; Smoking: 1855; Obese: 1973. But of note is that this study did not use discounting. |
| Hayashida et al 2009 [34] | Smokers had lower lifetime medical costs (albeit a small difference) | This Japanese modeling study was based on data from an actual cohort study with health cost data from individuals aged between 40 and 90 years (DR=3%). It found higher death rates in smokers (with lives that were on average 3.5 years shorter than non-smokers). Also that “although non-smokers had lower long-term cumulative medical expenditures between 64 and 81 years of age, their lifetime medical expenditures were higher by a minimal amount”. That is cumulative medical expenditure of US$ 49,980 vs US$ 51,771 (smokers vs non-smoking males). “We also observed tendencies for smokers to have higher inpatient expenditures, but non-smokers to have higher outpatient expenditures.” “There was little difference between smokers and non-smokers in their long-term cumulative medical expenditures at the discount rate of 5%.” At the DR=0% level the difference was larger (approximately US$ 112,000 in smokers vs 120,000 in non-smokers – based on results in Figure 1). Other aspects of note:   - Study included unrelated (non-tobacco) disease costs: Yes (fully captured given the cohort study data) - Study modeled the timing of costs: This was not dealt with precisely given that this study averaged the cost data to some degree: “we calculated the annual medical expenditures in 5-year age groups, but calculated that of those who died in their 40s as a 10-year age group because data were sparse”. - Study applied non-allocated health costs to all people: Yes (fully captured given the cohort study data) |
| van Baal et al 2008 [5] | Smokers had lower lifetime health costs (compared to obese and healthy populations) – albeit depending on the discount rate | This study compared annual and lifetime medical costs attributable to obesity with similar costs attributable to smoking. Three cohorts of people aged 20 years old were considered: obese, smokers and healthy-living. Cost of illness (COI) data from The Netherlands for 2003 were used to estimate health-care expenditure for each of three cohorts. The System of Health Accounts (SHA) was considered in the baseline analysis. The RIVM-CDM model was used to link prevalence of disease with risk factors and the model outcomes (prevalence) by disease were multiplied by the annual cost of disease. To calculate unrelated health care costs (for all “other” diseases), the number of survivors from the RIVM-CDM model was multiplied by age and sex-specific cost profiles of ‘‘remaining’’ costs. These annual related and unrelated cost where added over remaining lifetime to obtain lifetime costs. Expected remaining lifetime health costs for 20 year olds were: 250,000, 281,000 and 220,000 in 2003 Euros respectively for obese, healthy and smoker cohorts. Sensitivity analysis showed that under 3% and 4% of discounting, reduced smoking would result in additional healthcare costs of 7.1 and 3.4 million Euros respectively and it would need at least 5.7% discounting to make smoking reduction interventions cost saving. Smoking resulted in lower lifetime health-care costs for all seven scenario studied. Of note was Scenario 5 which adopted a narrower definition of healthcare costs by excluding all expenditure on nursing and residential care mentioned in the SHA definition. These costs can cause substantial variation in cost-of-illness estimations between countries. The narrower set of costs improves the international comparability of the figures presented. But the results still showed lower costs for smokers: 204,000, 215,000 and 186,000 Euros respectively for obese, healthy and smoker cohorts. |
| Vijgen et al 2008 [35] | Smoking cessation increased lifetime health costs | The aim of this study was to evaluate cost-effectiveness of the Dutch school-based smoking education programme. Savings for smoking-related diseases and differences in total health care costs were taken into account along with intervention costs. The RIVM-CDM model was used to simulate the long-term effects of differences in smoking prevalence on the incidence, prevalence, mortality and costs of 14 smoking-related diseases. Assuming 32 quit from a cohort of 1000 adolescents, the cost-savings for smoking-related diseases (15,800 Euros) were outweighed by future increases in health care costs of diseases unrelated to smoking. The cumulative difference in total health care cost was 21,600 Euros. All costs were discounted at 4%. |
| **Mixed results – depending on age/sex group or timing** | | |
| Yang et al 2012 [26] | Small lifetime increase in health costs from quitting overall but not in some groups (including females overall) | This US study found that the: “average lifetime health care expenditures rise by about $1,700 per quitter if cessation-related weight gain is not avoided and by considerably less—$1,000 per quitter—if it is.” Also that, “Overall, the average net lifetime health care cost increase of $1,700 or less per ex-smoker would be modest and, for employed people, more than offset by even one year’s worth of productivity gains.” The extra lifetime cost was for men and quitting was cost saving for women overall – though not in some age groups (all base case analysis had DR=3%). Also quitting was cost saving in all female age groups when cessation-related weight gain was not considered. There are questions about the generalisability of this study as it was of a military and ex-military population. Other aspects of note:   - Study included unrelated (non-tobacco) disease costs: Yes (48 disease were modeled of which 15 were tobacco-related). - Study modeled the timing of costs (initial period & near death): This may have been captured given the nature of the data source but timing issues are not specifically discussed. - Study applied non-allocated health costs to all people: Yes |
| Van Baal et al 2007 [10] | Overall extra cost of quitting in the long-term – but cost-savings in the first 20 years | This was another study from the Netherlands that used the RIVM-CDM model. The aim of it was to estimate the cost-effectiveness of tobacco tax increases from a health care perspective, explicitly considering medical costs in life years gained. The time horizon was 100 years. A tobacco tax increase resulted in estimated price increase of 10%. The cumulative difference in health care costs of smoking related diseases and cumulative difference in total health care costs were evaluated separately. Additional tax revenue was not taken into account. Like in the other similar studies, the savings in health care costs of smoking related diseases are outweighed by increases in the health care costs of diseases not related to smoking in life years gained. Cumulative increases in health care costs of smoking related diseases were 31, 62, 93 million Euros for 25%, 50% and 75% increase in quitting rates respectively, while taking into account unrelated future health care costs. All costs were discounted at 4%. Of note was that the costs took until after the year 2040 to peak and “cost savings were obtained over the first 20 years” from quitting. See S1 Text Table 1 for other details. |
| **Study suggesting smokers are relatively more expensive (health system costs)*** | | |
| Rasmussen et al 2005 [27] | Savings from quitting smoking in all adult age groups (and both sexes) | In this study from Denmark the authors found that total lifetime health cost savings at all ages, but particularly for those men and women who quit at age 35 years (DR=5%). Eg, for the 35 year old age group of women, these direct health cost savings were around 9,000 Euros for light smokers, 12,000 for moderate smokers, and 15,000 for heavy smokers (with fairly similar, albeit lower, results for men). The equivalent savings for the older age group (65 years) were all under 8000 Euros. There were still sizeable cost savings when DR=8%. This study also considered productivity costs. Other aspects of note:   - Study included unrelated (non-tobacco) disease costs: Yes – used data from a previous study for never-smoker and ever-smoker health costs. Increased survival in these groups taken into account. - Study modeled the timing of costs (initial period & near death): No - Study applied non-allocated health costs to all people: Probably yes (though this is not entirely clear) |

* There were excluded studies which considered lifetime cost savings from reduction of tobacco-related diseases but which did not consider the extra health costs associated with increased survival in people quitting smoking [31-33, 36].

**References**

1. Cobiac LJ, Ikeda T, Nghiem N, Blakely T, Wilson N. Modelling the implications of regular increases in tobacco taxation in the tobacco endgame. Tob control. 2015;24: e154–160.

2. The Parliament of the Commonwealth of Australia. Excise Tariff Amendment (Tobacco) Bill 2014. Canberra: The Parliament of the Commonwealth of Australia. Available: http://www.austlii.edu.au/au/legis/cth/bill_em/etab2014276/memo_0.html. Accessed 22 June 2014.

3. Hanewinkel R, Isensee B. Five in a row--reactions of smokers to tobacco tax increases: population-based cross-sectional studies in Germany 2001-2006. Tob control. 2007;16(1): 34-37.

4. Over EA, Feenstra TL, Hoogenveen RT, Droomers M, Uiters E, van Gelder BM. Tobacco control policies specified according to socioeconomic status: health disparities and cost-effectiveness. Nicotine Tob Res. 2014;16(6): 725-732.

5. van Baal P, Polder J, de Wit G, Hoogenveen R, Feenstra T, Boshuizen H, et al. Lifetime Medical Costs of Obesity: Prevention No Cure for Increasing Health Expenditure. PLoS Med. 2008;5(2): e29.

6. Over EA, Feenstra TL, Hoogenveen RT, Droomers M, Uiters E, van Gelder BM. Reply to the comment by Van der Deen et al.: "Possible methodological reason for the finding that 'Neither tax increase nor reimbursement reduced health disparities'". Nic Tob Res 2014;16(7):1031-1032.

7. Emery S, White MM, Gilpin EA, Pierce JP. Was there significant tax evasion after the 1999 50 cent per pack cigarette tax increase in California? Tobacco control. 2002;11(2):130-134.

8. Vos T, Carter R, Barendregt J, Mihalopoulis C, Veerman L, Magnus A, et al. Assessing Cost-Effectiveness in the Prevention (Ace-Prevention): Final Report. Brisbane and Melbourne: University of Queensland and Deakin University; 2010.

9. Reed H. The effects of increasing tobacco taxation. A cost benefit and public finances analysis. London: UK ASH (Action on Smoking and Health), Landman Economics. Available: <http://www.ash.org.uk/tax/analysis;> 2010.

10. van Baal PH, Brouwer WB, Hoogenveen RT, Feenstra TL. Increasing tobacco taxes: a cheap tool to increase public health. Health policy. 2007;82(2):142-52.

11. Ahmad S. Increasing excise taxes on cigarettes in California: a dynamic simulation of health and economic impacts. Prev Med. 2005;41(1):276-83.

12. Fishman PA, Ebel BE, Garrison MM, Christakis DA, Wiehe SE, Rivara FP. Cigarette tax increase and media campaign cost of reducing smoking-related deaths. Am J Prev Med. 2005;29(1): 19-26.

13. Ranson MK, Jha P, Chaloupka FJ, Nguyen SN. Global and regional estimates of the effectiveness and cost-effectiveness of price increases and other tobacco control policies. Nicotine Tob Res. 2002;4(3): 311-319.

14. Ortegon M, Lim S, Chisholm D, Mendis S. Cost effectiveness of strategies to combat cardiovascular disease, diabetes, and tobacco use in sub-Saharan Africa and South East Asia: mathematical modelling study. BMJ. 2012;344: e607.

15. Salomon JA, Carvalho N, Gutierrez-Delgado C, Orozco R, Mancuso A, Hogan DR, et al. Intervention strategies to reduce the burden of non-communicable diseases in Mexico: cost effectiveness analysis. BMJ. 2012;344: e355.

16. Higashi H, Truong KD, Barendregt JJ, Nguyen PK, Vuong ML, Nguyen TT, et al. Cost effectiveness of tobacco control policies in Vietnam: the case of population-level interventions. Appl Health Econ Health Policy. 2011;9(3): 183-196.

17. Asaria P, Chisholm D, Mathers C, Ezzati M, Beaglehole R. Chronic disease prevention: health effects and financial costs of strategies to reduce salt intake and control tobacco use. Lancet. 2007;370(9604): 2044-2053.

18. Brown T, Platt S, Amos A. Equity impact of population-level interventions and policies to reduce smoking in adults: a systematic review. Drug Alcohol Depend. 2014;138: 7-16.

19. Brown T, Platt S, Amos A. Equity impact of interventions and policies to reduce smoking in youth: systematic review. Tob Control. 2014; doi: 10.1136/tobaccocontrol-2013-051451.

20. Centers for Disease Control and Prevention. Response to increases in cigarette prices by race/ethnicity, income, and age groups-United States, 1976-1993. MMWR. 1998;47(29): 605-609.

21. Hill S, Amos A, Clifford D, Platt S. Impact of tobacco control interventions on socioeconomic inequalities in smoking: review of the evidence. Tob Control. 2014;23: e89-e97.

22. Chaloupka FJ, Straif K, Leon ME. Effectiveness of tax and price policies in tobacco control. Tob Control. 2011;20(3): 235-238.

23. IARC. Effectiveness of Tax and Price Policies for Tobacco Control. Lyon, France: International Agency for Research on Cancer, 2011.

24. Main C, Thomas S, Ogilvie D, Stirk L, Petticrew M, Whitehead M, et al. Population tobacco control interventions and their effects on social inequalities in smoking: placing an equity lens on existing systematic reviews. BMC Public Health. 2008;8: 178.

25. Max W. The financial impact of smoking on health-related costs: a review of the literature. AJHP. 2001;15(5): 321-331.

26. Yang W, Dall TM, Zhang Y, Zhang S, Arday DR, Dorn PW, et al. Simulation of quitting smoking in the military shows higher lifetime medical spending more than offset by productivity gains. Health Aff. 2012;31(12): 2717-2726.

27. Rasmussen SR, Prescott E, Sorensen TI, Sogaard J. The total lifetime health cost savings of smoking cessation to society. Eur J Public Health. 2005;15(6): 601-606.

28. van Baal PH, Feenstra TL, Polder JJ, Hoogenveen RT, Brouwer WB. Economic evaluation and the postponement of health care costs. Health Econ. 2011;20(4): 432-445.

29. Kanters TA, Brouwer WB, van Vliet RC, van Baal PH, Polder JJ. A new prevention paradox: the trade-off between reducing incentives for risk selection and increasing the incentives for prevention for health insurers. Social Sci Med. 2013;76(1): 150-158.

30. Slobbe LCJ, Kommer GJ, S m, J.M., Groen J, Meerding WJ, Polder JJ. Kosten van Ziekten in Nederland 2003 [Cost of illness in Netherland (2003)]. Bilthoven: Rijksinstituut voor Volksgezondheid en Milieu (RIVM); 2006.

31. Bolin K, Borgman B, Gip C, Wilson K. Current and future avoidable cost of smoking-estimates for Sweden 2007. Health policy. 2011;103(1): 83-91.

32. Magnus A, Cadilhac D, Sheppard L, Cumming T, Pearce D, Carter R. Economic benefits of achieving realistic smoking cessation targets in Australia. Am J Public Health. 2011;101(2): 321-327.

33. Cadilhac DA, Magnus A, Sheppard L, Cumming TB, Pearce DC, Carter R. The societal benefits of reducing six behavioural risk factors: an economic modelling study from Australia. BMC Public Health. 2011;11: 483.

34. Hayashida K, Imanaka Y, Murakami G, Takahashi Y, Nagai M, Kuriyama S, et al. Difference in lifetime medical expenditures between male smokers and non-smokers. Health Policy. 2010;94(1): 84-89.

35. Vijgen SM, van Baal PH, Hoogenveen RT, de Wit GA, Feenstra TL. Cost-effectiveness analyses of health promotion programs: a case study of smoking prevention and cessation among Dutch students. Health Educ Res. 2008;23(2): 310-318.

36. Bauld L, Boyd KA, Briggs AH, Chesterman J, Ferguson J, Judge K, et al. One-year outcomes and a cost-effectiveness analysis for smokers accessing group-based and pharmacy-led cessation services. Nicotine Tob Res. 2011;13(2): 135-145.
