# Supplementary material for: Correction: Health, Health Inequality, and Cost Impacts of Annual Increases in Tobacco Tax: Multistate Life Table Modeling in New Zealand
Source: PLoS Med. 2016 Dec 22;13(12):e1002211. doi: 10.1371/journal.pmed.1002211 (PMC5179055; doi:10.1371/journal.pmed.1002211)
Supplement: S1 Manuscript — (DOCX) [file pmed.1002211.s001.docx]

**Health, Health Inequality, and Cost Impacts of Annual Increases in Tobacco Tax: Multistate Life Table Modeling in New Zealand**

Tony Blakely^1^*, Linda J. Cobiac^1,2^, Christine L. Cleghorn^1^, Amber L. Pearson^1,3^, Frederieke S. van der Deen^1^, Giorgi Kvizhinadze^1^, Nhung Nghiem^1^*,* Melissa McLeod^1^, Nick Wilson^1^

1 Burden of Disease Epidemiology, Equity and Cost Effectiveness Programme, Department of Public Health, University of Otago, Wellington, New Zealand,

2 British Heart Foundation Centre on Population Approaches to NCD Prevention, Nuffield Department of Population Health, University of Oxford, Oxford, United Kingdom,

3 Department of Geography, Michigan State University, East Lansing, Michigan, United States of America

* [tony.blakely@otago.ac.nz](mailto:tony.blakely@otago.ac.nz)

**Abbreviations:** ENDS, electronic nicotine delivery systems; APC, annual percentage change; ASH, Action on Smoking and Health; BAU, business as usual; CFR, case-fatality rates; CHD, coronary heart disease; COPD, chronic obstructive pulmonary disease; CPS II, cancer prevention study 2; DR, disability rate; FCTC, Framework Convention on Tobacco Control; LRTI, lower respiratory tract infection; NCD, noncommunicable disease; NZBDS, New Zealand Burden of Disease Study; QALY, quality-adjusted life-year; RR, relative risk; SD, standard deviation; SES, socioeconomic status; SNZ, Statistics New Zealand; SRD, standardized rate differences; SRR, standardized rate ratio; UI, uncertainty interval; YLD, years of life lived with disability

# Abstract

**Background:** Countries are increasingly considering how to reduce or even end tobacco consumption, and raising tobacco taxes is a potential strategy to achieve these goals. We estimated the impacts on health, health inequalities, and health system costs of ongoing tobacco tax increases (10% annually from 2011 to 2031, compared to no tax increases from 2011 [“business as usual,” BAU]), in a country (New Zealand) with large ethnic inequalities in smoking-related and noncommunicable disease (NCD) burden.

**Methods and Findings:** We modeled 16 tobacco-related diseases in parallel, using rich national data by sex, age, and ethnicity, to estimate undiscounted quality-adjusted life-years (QALYs) gained and net health system costs over the remaining life of the 2011 population (*n* = 4.4 million). A total of 275,000 (95% uncertainty interval [UI]:158,000–431,000) QALYs were gained among the 2011 cohort exposed to annual tobacco tax increases, compared to BAU, and cost savings were US$2,620 million (95% UI: US$1,570 to US$4,030). QALY gains and cost savings took 50 y to peak, owing to such factors as the price sensitivity of youth and young adult smokers. The QALY gains per capita were 4.9 times greater for Māori (indigenous population) compared to non-Māori because of higher background smoking prevalence and price sensitivity in Māori. Health inequalities measured by differences in 45+ y-old standardized mortality rates between Māori and non-Māori were projected to be 2.49% (95% UI: 1.61% to 3.69%) less in 2041 with ongoing tax rises, compared to BAU. Percentage reductions in inequalities in 2041 were maximal for 45–64 -y-old women (3.12%). As with all such modeling, there were limitations pertaining to the model structure and input parameters.

**Conclusions:** Ongoing tobacco tax increases deliver sizeable health gains and health sector cost savings and are likely to reduce health inequalities. However, if policy makers are to achieve more rapid reductions in the NCD burden and health inequalities, they will also need to complement tobacco tax increases with additional tobacco control interventions focused on cessation.

# Introduction

Tobacco use is a leading risk factor for health loss internationally [[1](#_ENREF_1)]. Therefore, reducing tobacco use is a leading strategy proposed for achieving the global “25 x 25 NCD [noncommunicable disease] mortality reduction targets” [[2](#_ENREF_2)].

Tobacco taxation is an important tobacco control measure, with advantages that include (i) being a typically well-established function of government, (ii) having a strong evidence-base for effectiveness [[3](#_ENREF_3)], and (iii) having the potential benefit of generating extra government revenue that can then fund other aspects of tobacco or NCD control.

We identified 11 relevant studies on tobacco tax in which both a health impact and a costing metric were estimated as outputs (see S1 Text). Most of these studies were for developed countries (six out of 11), four were for developing countries, and one was a mix. All studies reported overall health gains from tobacco tax increases, and these were reported as either very cost-effective or were cost saving. However, study methodologies varied greatly, e.g., in the use of different discount rates and the different considerations around which costs were included. Some studies considered cost offsets from tobacco-related diseases only, and only two studies considered these as well as the health costs associated with extra life lived as a result of the tax intervention (despite the latter probably being the most appropriate [[4](#_ENREF_4)]). Also, none of the studies considered repeated annual tobacco tax increases (even though these are increasingly used internationally, e.g., in New Zealand (every year from 2010 to 2015 and planned to 2016 [[5](#_ENREF_5)]), Australia (four increases legislated from 2013 [[6](#_ENREF_6)]), and Germany (five increases in 2001–2006 [[7](#_ENREF_7)]). Indeed, to achieve marked reductions in tobacco-related disease, repeated tax increases over many years are probably necessary.

Only three of the 11 relevant studies we identified considered differences in health gain by sex, and for all these the gain was greater for men. Also, only one study considered socioeconomic status (SES) [[8](#_ENREF_8)], finding that the quality-adjusted life-years (QALYs) gained per person were greatest in the lowest (least-educated) SES group.

Our background literature search (detailed in S1 Text) also identified six systematic reviews on the impact of tobacco prices/taxes on equity that had been published since 2005. The two most recent such reviews indicated that tobacco price/tax increases tended to have a positive impact on equity (i.e., reduction in inequalities in smoking prevalence by SES) for both adults [[9](#_ENREF_9)] and youth [[10](#_ENREF_10)]. The four other reviews also reported this same general pattern, albeit more tentatively. However, despite this body of work on equity, we could not identify quantitative assessment of the health gain (e.g., mortality rate differences by socioeconomic group) that might arise as a result of regularly increased tobacco taxes.

These knowledge gaps in how tobacco taxes might work matter for governments that are seeking incremental reductions in the tobacco epidemics in their country but are particularly relevant to those aiming to achieve a tobacco “endgame” (e.g., Finland [[11](#_ENREF_11)], Ireland [[12](#_ENREF_12)], Scotland [[13](#_ENREF_13)], multiple Pacific Island states [[14](#_ENREF_14)], and New Zealand [[15](#_ENREF_15)] currently have official endgame targets). The latter countries particularly need to know how much of the potential health gain from eliminating tobacco in a country can be achieved through tobacco tax increases relative to “business as usual” (BAU) tobacco control measures.

New Zealand is a relatively informative setting in which to address the knowledge gaps outlined above. It has rich data on disease incidence and mortality by sociodemographics, including attribution of publicly funded health care costs to individually linked health datasets. The country is also characterized by marked health inequalities between the indigenous Māori (15% of the total population) and non-Māori, with much higher Māori smoking prevalence (33% compared to 14% for New Zealand Europeans in 2013 [[16](#_ENREF_16)]), all-cause mortality rates over twice as high for Māori [[17](#_ENREF_17)], and a life expectancy gap of 7.3 y between Māori and non-Māori in 2010–2012. Smoking is a major cause of health inequalities in New Zealand [[18](#_ENREF_18),[19](#_ENREF_19)], as is true in many other settings internationally. Furthermore, New Zealand has an endgame goal for smoking [[15](#_ENREF_15)] and is also implementing a program of regular tobacco tax increases (as detailed above). Other than this particular program, it is a fairly typical high-income country in terms of utilizing many other tobacco control measures, such as tight restrictions on tobacco marketing, smoke-free laws for indoor environments (including bars and restaurants), restrictions on tobacco displays in retail outlets, provision of pictorial warnings on tobacco packaging, the episodic (albeit low-budget [[20](#_ENREF_20)]) use of mass media campaigns, and the provision of subsidized smoking cessation support such as a national Quitline service.

Given this background, the objectives of this paper were to estimate the future impact of annual tobacco tax increases of 10% per annum from 2011 to 2031 (as per a continuation of the current New Zealand strategy), compared to no tax increases from 2011 (defined as the BAU comparator in this paper), on the magnitude and timing of (i) health gains, (ii) changes in net health system expenditure, and (iii) changes in health inequalities (while incorporating uncertainty).

# Methods

## Overview of Modeling

The entire New Zealand population alive in 2011 was simulated to death in a life table that uses projected all-cause mortality and morbidity rates by sex, age, and ethnicity (Māori and non-Māori). In parallel, there were 16 tobacco-related disease life tables, in which proportions of the population simultaneously reside: coronary heart disease, stroke, chronic obstructive pulmonary disease (COPD), lower respiratory tract infection (LRTI), and multiple cancers: lung, esophageal, stomach, liver, head and neck, pancreas, cervical, bladder, kidney, endometrial, melanoma, and thyroid (with smoking protecting against the latter three cancers [[21](#_ENREF_21)]). The proportion of the population (by sex, age, and ethnicity) in each disease life table is a function of the disease incidence (i.e., inflow) and case fatality (and remission for cancers) (i.e., outflow). An overview of the model, as well as additional details about the methods, can be found in S2 Text.

The input data consists of BAU and “intervention” parameters (see S2 Text). The BAU parameters include disease-specific epidemiological parameters (with projected trends to 2026), health system costs for different states as calculated for 2011, and projected future smoking prevalence.

The intervention effect is captured through changes to BAU smoking prevalence from tax increases acting through price elasticities of demand. The change in smoking prevalence is then combined with relative risks for the smoking-incidence rate ratios to generate population impact fractions (percentage reductions in future tobacco-related disease incidence) that alter the inflow to the tobacco-related disease life tables.

Modeling the future is inherently uncertain. For example, will past trends in initiation and cessation rates and price elasticities apply into the future? We addressed this by specifying uncertainty about most input parameters to the modeling. For relative risks of smoking to disease incidence, we used confidence intervals from relevant studies (but doubled their width in scenario analyses). For other variables, we used the following generic approach to distributions from which values are sampled in Monte Carlo simulations: a standard deviation (SD) of +/- 5% for reasonably certain variables (e.g., disease incidence rates in 2011); +/- 10% SD for moderately uncertain variables (e.g., health system cost and morbidity); +/- 20% SD for more uncertain variables when extrapolated to the future (e.g., price elasticities and BAU cessation and initiation rates before any tax). Whilst it is important to include plausible uncertainty in the modeling, it is also important to note that our uncertainty analyses are a scenario in and of themselves.

The difference between the intervention and BAU scenarios is quantified as incremental QALYs and health system costs (with uncertainty).

## Input Parameters

**BAU parameters.** All input parameters are shown in Table 1. Each tobacco-related disease had three parameters specified by each sex, age, and ethnicity in 2011: incidence, prevalence, and case fatality (see “Appendix C” in S2 Text). Remission was assumed “zero” for noncancers (except LRTI) but specified as a fourth parameter for cancers. These parameters were calculated using DISMOD II [[22](#_ENREF_22)], a tool that produces epidemiologically and mathematically coherent sets of parameters for a given disease. The inputs into DISMOD were weighted based on their “reliability”, e.g., full weight given to cancer incidence and population mortality rates due to the high quality of data (see an example in “Appendix A” in S2 Text). Using the DISMOD outputs for 2011, we additionally specified future annual percentage changes (to 2026 and then no change) in cancer incidence, case fatality, and remission, using regression estimates of trends from historic data. Trends in COPD incidence and case fatality until 2026 were difficult to estimate because of the evolving nature of the tobacco epidemic, so we noted that COPD mortality (all sexes and ethnic groups combined) is declining at about 2% per annum, assumed that this was split fifty-fifty between incidence and case fatality trends, and applied this uniformly to all four sex x ethnic group categories (given the recent similar relative reduction in tobacco use by these groups). Trends in other disease incidence and case fatality were sourced from the New Zealand Burden of Disease Study (NZBDS) [[23](#_ENREF_23)].

Table 1. Input parameters.

| **Parameter** | **Comment** | **Trend, Uncertainty, and Any Scenario Analyses** |
| --- | --- | --- |
| ***Baseline*** |  |  |
| Population | Statistics New Zealand (SNZ) population estimates for 2011 by sex, age group, and ethnicity. | Nil uncertainty. |
| All-cause mortality rates | SNZ mortality rates by sex, age, and ethnicity for 2011. | Trend is equal to the weighted sum (weights = proportion of deaths in 2011, by sex, age, and ethnicity) of trends for each explicitly modeled disease (i.e., [incidence] + [case-fatality] – [remission]), and the remaining causes of death are consistent with long-run mortality trends [[24](#_ENREF_24)] (annual 2.25% mortality decline for Māori and 1.75% per annum for non-Māori). Trends were modeled out to 2026, with 0% per annum decline for both ethnic groupings thereafter. Uncertainty: nil. Scenario analysis: extend trends indefinitely out beyond 2026. |
| Disease-specific incidence, prevalence, and case-fatality rates (and remission rates) | For each tobacco-related disease, coherent sets (by sex, age, and ethnicity) of incidence rates, prevalence, case-fatality rates (CFR), and remission rates (zero for noncancers, the complement of the CFR for cancers to give the expected 5-y relative survival) were estimated using DISMOD II [[22](#_ENREF_22)]. | Cancer incidence and CFR annual percentage change (APC) trends based on historic trends [[25](#_ENREF_25),[26](#_ENREF_26)], projected out to 2026, then constant. (Future prevalence changes dynamically with model.) Uncertainty: Starting in 2011, rates all +/- 5% SD, correlations 1.0 between four sex × ethnic group categories for all diseases. APC all +/- 0.5% SD normal, correlations 1.0 between 4 sex × ethnic groups for all diseases. Scenario analyses: extend trends indefinitely out beyond 2026; halve and double SD uncertainty. |
| Total morbidity per capita in 2011 | The per capita rate of years of life lived with disability (YLD) from the NZBDS [[23](#_ENREF_23)] by sex, age, and ethnicity. | No trend (i.e., assumed constant into the future). Uncertainty +/- 10% SD log-normal. Scenario analysis: halve and double SD uncertainty. |
| Disease morbidity rate per capita | Each disease was assigned a disability rate (DR; by sex and age) equal to YLDs for that disease (scaled down to adjust for comorbidities) from the 2006 NZBDS [[23](#_ENREF_23)] projected forward to 2011, divided by the disease prevalence (above). This DR was assigned to the proportion of the cohort in each disease state. | No trend. Uncertainty: +/- 10% SD normal. Scenario analysis: halve and double SD uncertainty. |
| Health system costs | Linked health data (hospitalizations, inpatient procedures, outpatients, pharmaceuticals, laboratories, and expected primary care usage) for each individual in New Zealand (NZ) for the period 2006–2010 had unit costs assigned to each event, and then five health system costs (NZ$2011; by strata of sex and age) were estimated (see S2 Text). | No trend. Uncertainty: +/- 10% SD log-normal. Scenario analysis: halve and double SD uncertainty. |
| Tobacco smoking prevalence | As in the 2013 NZ census, back-estimated to 2011 using annual net cessation and initiation rates and allowing for tax in 2011 to 2013 (using price elasticities detailed below). | BAU calculated using 2006 to 2013 census-derived trends in initiation and net cessation rates (stripped of “tax effect”) [[16](#_ENREF_16)], projected out to the future. |
|  |  | *Annual proportionate reductions in initiation age 20:* |
|  |  | - Non-Māori: male 0.0339, female 0.0276 |
|  |  | - Māori: male 0.0288, female 0.0322 |
|  |  | Uncertainty: +/- 20% SD beta correlations 1.0 between four sex × ethnic groups. Scenario analysis: halve and double SD uncertainty. |
|  |  | *Annual net cessation rates:* |
|  |  | 20–34 y of age: |
|  |  | - Non-Māori: male 0.0414, female 0.0554 |
|  |  | - Māori: male 0.0393, female 0.0451 |
|  |  | 35–54 y of age: |
|  |  | - Non-Māori: male 0.0384, female 0.0431 |
|  |  | - Māori: male 0.0369, female 0.0472 |
|  |  | 55+ y of age: |
|  |  | - Non-Māori: male 0.0722, female 0.0714 |
|  |  | - Māori: male 0.0769, female 0.0699 |
|  |  | Uncertainty: +/- 20% SD beta, correlations 1.0 between 12 sex × age × ethnic group categories. Scenario analysis: halve and double SD uncertainty. |
| ***Intervention*** |  |  |
| Cost of a law | Cost of a new law in NZ to mandate the series of annual tax rises, NZ$3.54 million [[27](#_ENREF_27)]. | Uncertainty: gamma SD NZ$1.05 million in 2011 only |
| Excise tax increase | 10% | Annually from 2011 to 2031, with scenario analyses about number of years. Uncertainty = +/- 10% (or 1 percentage point of 10% excise tax) SD normal. Scenario analyses: halve and double SD uncertainty. |
| Tobacco tax price elasticities | Non-Māori: Price elasticities of -0.38 (for 15–20-y-olds), -0.29 (for 21–24-y-olds), -0.19 (for 25–34-y-olds), and -0.10 (for 35+ y-olds) for smoking prevalence were applied in the year the tax rise was implemented [[5](#_ENREF_5),[16](#_ENREF_16),[28](#_ENREF_28)]. Māori: Within each iteration, we scaled up the non-Māori price elasticity by 20% for Māori, given economic theory, the patterns in the international literature for other social groupings [[3](#_ENREF_3),29], and some New Zealand evidence for increased price sensitivity for Māori [[30](#_ENREF_30),[31](#_ENREF_31)]. | No trend. Uncertainty: non-Māori, +/- 20% normal, correlated 1.0 across four age groups; Māori absolute scalar of +20% within each age group, +/- 10% normal (i.e., 95% range of absolute scalar of 0.4% to 39.6%). Scenario analyses: no ethnic scalar; halve and double SD uncertainty. |
| Relative risks for smoking and disease incidence | Relative risks of disease incidence for the association of current (or ex-smoker) with never smoker were sourced from NZ linked census-cancer [[21](#_ENREF_21)] and census-mortality [[32](#_ENREF_32)] (censuses include smoking question) and CPS II data for respiratory diseases [[33](#_ENREF_33)]. Attenuation over time since quitting for ex-smokers was modeled using equations and coefficients from Hoogenveen et al. [[34](#_ENREF_34)]. | Standard errors of regression coefficients as published (and in S2 Text—log-normal). Scenario analyses: using higher cancer prevention study 2 (CPS II) relative risks (RRs) for coronary heart disease (CHD) and stroke, compared to NZ RRs; double SD. |

Overall morbidity, by sex, age and ethnicity, was quantified in the model using the years of life lived with disability (YLDs) from the NZBDS, divided by the population count to give “prevalent” YLDs. Disease-specific morbidity was assigned in each disease state, as the total comorbidity-adjusted YLDs for that disease divided by the prevalent population. The health status valuations of these YLDs were disability weights derived from the Global Burden of Disease using pairwise comparisons from multicountry surveys [[35](#_ENREF_35)], as opposed to, for example, disutilities from the EuroQol.

Health system costs by sex and age group, in 2011 New Zealand dollars (see “Appendix D” in S2 Text), were determined using individually linked data for publicly funded health events occurring in 2006–2010 (hospitalizations, inpatient procedures, outpatients, pharmaceuticals, laboratories, and expected primary care usage). Building on an existing framework [[36](#_ENREF_36)] for capturing the timing of health system costs, we assigned everyone “alive” in the model a sex and age-specific annual cost of a citizen without a tobacco-related disease and not in the last 6 mo of life. We then assigned additional disease-specific excess costs for people in the first year of diagnosis or in the last 6 mo of life if dying of the given disease and, otherwise, for prevalent cases of each disease.. We model costs over the lifetime of the cohort, including costs both related and unrelated to the tobacco diseases modeled (meaning that increased longevity due to tobacco control contributed to increased health system costs for some cohort members).

In parallel with the multistate life table, a BAU projection of future tobacco prevalence was estimated assuming APCs in initiation (smoking rates age 20) and net cessation (by sex, age, and ethnicity) between the 2006 and 2013 census (removing the tax effect from 2010 to 2013) continued into the future, combined with mortality rates by smoking status, using methods described previously [[16](#_ENREF_16),[28](#_ENREF_28)].

**Intervention parameters.** The fixed annual 10% tax increase (as per the current New Zealand situation) impacted on tobacco smoking prevalence through price elasticities, whereby current smoker prevalence reduced by 3.8% for 15–20-y-old smokers compared to a 1.0% reduction among 35+ y-old smokers (due to lower price elasticities at older ages; Table 1). This quitting effect applied in the year of tax rise only, consistent with other tobacco tax models. Cumulative 10% tax increases resulted in the 2011 inflation adjusted pack price increasing from NZ$14 in 2011 to NZ$37 in 2025 and NZ$58 in 2031, with pack prices reflecting modest growth in the illegal market as per our previous work [[5](#_ENREF_5)]. Theoretically, one expects price elasticities to be greater among lower-income populations, and therefore one would expect higher price elasticities among Māori compared to non-Māori. Whilst no direct evidence exists, there are both indirect evidence (experimental studies of Māori and non-Māori smoker intentions in New Zealand [[30](#_ENREF_30)]) and parallel evidence (higher price elasticities for food among Māori [[31](#_ENREF_31)]).

Relative risks of the current–never smoker association were calculated from linked New Zealand census-cancer data [[21](#_ENREF_21)], linked census-mortality data for cardiovascular disease [[32](#_ENREF_32)] (assuming mortality rate ratios are the same as incidence rate ratios; scenario analyses using Cancer Prevention Study cardiovascular disease relative risks [[33](#_ENREF_33)] are presented in S2 Table), and elsewhere for COPD and LRTI [[33](#_ENREF_33)]. Among ex-smokers, the current–never rate ratio was specified to decay according to equations provided by Hoogenveen et al. [[34](#_ENREF_34)] and assuming no excess risk after 20 y.

This difference between BAU and “under tax” future tobacco prevalence projections (i.e., the difference between the two projections shown in Fig 1) was then mathematically combined with the rate ratios to calculate population impact fractions and from there the percentage reduction in incidence of each tobacco-related disease.

**Fig 1. Future smoking prevalence in the New Zealand 2011 population by scenario and QALY gains between scenarios.** QALYs gained for areas between the curves A, B, and C are undiscounted. QALYs discounted at 3% are (A) 655,000, (B) 58,000, and (C) 391,000. “Smoking cessation stops, initiation continues” = scenario of no further net smoking cessation among those already smoking in 2011, ongoing initiation of people aged up to 20 y of age in 2011. The prevalence therefore increases up to 2031 in this closed cohort (due to new smokers outnumbering differential deaths by smoking status), then declines over time because of aging of the population and the higher mortality rate of smokers. BAU = scenario of net cessation and initiation rate trends observed between 2006 and 2013 censuses continuing into the future (tax effects removed), including differential mortality from smoking (i.e., additionally allowing for higher mortality of current (and ex-) smokers that will also decrease prevalence).

## Model Validation

Our model is built with extensive and rich New Zealand epidemiological and costing data. The epidemiological parameters of incidence, case fatality, and (for cancers) remission are also estimated using epidemiological equations (i.e., DISMOD II, above) to be coherent. Nevertheless, necessary structural assumptions (e.g., independence of disease incidence) may render the model inaccurate. Therefore, we compared mortality rate and count outputs from the model for various age groups with all trends set to zero and compared them to age-specific mortality rates. There was reasonably close agreement for stroke, coronary heart disease, and lung cancer (see “Appendix B” in S2 Text).

## Modeling and Analysis

The scenarios were simulated 4,000 times in Microsoft Excel (with the Ersatz add-in, an extension to Excel that allows Monte Carlo simulation, uncertainty analysis, and other epidemiological and health economic decision modeling functions ([www.epigear.com](http://www.epigear.com))), with each simulation involving a random draw from the probability density function about those parameters specified with uncertainty in Table 1. The two key outputs were QALYs and net health system costs (which unless stated otherwise are incremental between BAU and the tax intervention).

The net health system cost was the net of the intervention cost (cost of a new law) and any difference in projected future health system expenditure, converted to United States dollars using OECD purchasing power parity for 2011 (1.486 US$ to 1 NZ$).

Future mortality rates and, in particular, rate differences and ratios between Māori and non-Māori were also extracted from the model to estimate changes in all-cause mortality inequalities.

In addition to the intervention of interest (annual 10% tobacco tax increases) and the main comparator (BAU projections of smoking prevalence), we also modeled two additional scenarios to capture the full envelope of health gains possible with tobacco control: (A) no cessation among those already smoking in 2011 and ongoing initiation for those aged less than 20 y in 2011 and (B) complete smoking cessation in 2011.

# Results

Continuing the existing New Zealand policy of annual 10% increments in tobacco tax for the whole 2011 to 2031 period gained an additional 275,000 QALYs compared to BAU (Fig 1; 58,000 discounted at 3%). A counterfactual scenario of no further smoking from 2011 (but allowing for gradual transition of disease rates from current to never smoker rates) would result in an additional 1,290,000 QALYs (391,000 discounted at 3%) gained over and above the tax scenario. Put another way, annual 10% tax increases could achieve 18% (= 275,000 / [275,000 + 1,290,000]) of potential health gain from reducing tobacco use remaining beyond BAU.

Table 2 shows the QALY gains and health system costs averted for the tax intervention compared to BAU, by sex, age, and ethnicity. QALY gain occurred particularly for Māori at 47% of the total (129,000/275,000), despite Māori being only 15% of the population in 2011. That is, there would be 4.9 times as much health gain per capita for Māori (191/39). By age, health gains were largest for those aged less than 45 y in 2011 and minimal for those aged 65+ y in 2011.

By disease, 53.1% of QALY gains were through preventing COPD (with a similar mortality and smoking relative risk to lung cancer but high incidence and prevalence), 9.5% through CHD, 8.4% through stroke, and 27.8% through lung cancer (S1 Table; the contribution of CHD increases to 15.3% in a scenario analysis using CPS II relative risks). Net health system savings were NZ$3,880 million (undiscounted; US$2,620 million, 95% UI: US$1,570 to US$4,030) over the remaining life of the 2011 cohort.

Table 2. QALYs gained and health system costs (NZ$) averted from a 10% per annum increase in tobacco tax from 2011 to 2031, among the New Zealand population alive in 2011 (0% discounting).

|  | **Non-Māori** | | **Māori** | | | **Ethnic Groupings Combined** | |
| --- | --- | --- | --- | --- | --- | --- | --- |
| **Sex and age (in 2011)** | **QALYs** | **Cost savings (millions)** | **QALYs** | **QALYs—Equity†** | **Cost savings (millions)** | **QALYs** | **Net cost savings (millions) ‡** |
| Sex and age groups combined | 145,000 (81,300 to 233,000) | $2430 ($1430 to $3830) | 129,000 (77,300 to 195,000) | 195,000 (117,000 to 305,000) | $1450 ($900 to $2160) | 275,000 (158,000 to 431,000) | $3880 ($2330 to $5970) |
| *Men* |  |  |  |  |  |  |  |
| 0–14 y-olds | 29,600 | $604 | 29,100 | 44,700 | $509 | 58,700 | $1,110 |
| 15–24 y-olds | 18,000 | $321 | 11,900 | 18,400 | $181 | 29,900 | $502 |
| 25–44 y-olds | 20,000 | $285 | 7,840 | 12,500 | $93 | 27,800 | $378 |
| 45–64 y-olds | 7,130 | $81 | 1,620 | 2,870 | $15 | 8,750 | $97 |
| 65+ y-olds | 323 | $3.0 | 31 | 63 | $0.4 | 353 | $3.3 |
| All ages | 75,100 | $1,290 | 50,500 | 78,600 | $799 | 126,000 | $2,090 |
| *Women* |  |  |  |  |  |  |  |
| 0–14 y-olds | 30,000 | $560 | 44,700 | 64,100 | $410 | 74,700 | $969 |
| 15–24 y-olds | 15,700 | $273 | 17,800 | 26,100 | $143 | 33,600 | $417 |
| 25–44 y-olds | 17,400 | $242 | 13,100 | 20,100 | $80 | 30,500 | $322 |
| 45–64 y-olds | 6,580 | $65 | 3,050 | 5,230 | $17 | 9,630 | $82 |
| 65+ y-olds | 370 | $2.5 | 90 | 170 | $0.7 | 460 | $3.2 |
| All ages | 70,100 | $1,140 | 78,700 | 116,000 | $651 | 149,000 | $1,790 |
| ***Per capita (QALYs/1,000 people and $)*** | 39 | $647 | 191 | 288 | $2,130 | 62 | $873 |

† Māori “QALYs—Equity” are calculated using non-Māori background mortality and morbidity rates so as not to “penalize” Māori because of worse background mortality and morbidity.

**‡** Includes both the cost offsets and intervention cost, the latter being the cost of a law (NZ$3.5 million, 95% UI NZ$2.0 to NZ$6.2 million [[27](#_ENREF_27)]) to introduce tobacco taxes increases of 10% per annum to 2031, dis­tributed pro rata across all people alive in 2011. The cost of a law was not partitioned by age, sex, and ethnicity.

See S3 Table for uncertainty distributions.

Table 2 also shows an “equity analysis” for Māori in which non-Māori background mortality and morbidity were used (so as to “value” potential health gains from preventing tobacco-related diseases similarly between Māori and non-Māori [[37](#_ENREF_37)]). The health gains for Māori increased by 51% (129,000 to 195,000) and made the health gains per capita 7.4 times greater for Māori than non-Māori (288/39).

While health gains began immediately, they still took around five decades to peak (Fig 2). Net health system cost savings were over NZ$10 million per year by 2021 but also took five decades to peak at around NZ$160 million per year in 2061 (Fig 3). More apparent with undiscounted costs in Fig 3, annual health system savings became slightly negative late in the century (due to people living longer from not smoking and therefore incurring health system costs).

**Fig 2. Projected QALYs gained by future year for 10% per annum tax increases from 2011 to 2031, by sex and ethnicity (in the 2011 cohort of the New Zealand population without replacement)**. Similar graphs by age group in 2011 are shown in S1 Fig.

**Fig 3. Projected net health system cost savings by future year for 10% per annum tax increases from 2011 to 2031, by sex and ethnicity (in the 2011 cohort of the New Zealand population without replacement).** Similar graphs by age group in 2011 are shown in S1 Fig.

Under BAU, the Māori and non-Māori standardized mortality rates for those aged above 45 y were projected as 1,571.0 and 849.8 per 100,000 in 2041 (Table 3). With tax, these mortality rates would drop by 1.36% (95% UI 0.87% to 2.02%) and 0.39% (0.24% to 0.61%), respectively. In parallel, the standardized rate difference comparing Māori to non-Māori declined from 721.2 in BAU to 703.2 per 100,000 in the tax intervention (-2.49%), and the standardized rate ratio (SRR) declined from 1.848 to 1.830 (-2.11% reduction in “excess” SRR, i.e., SRR-1). Larger reductions in inequalities were seen for younger age groups and for women. The change in inequalities also varied by time into the future (Fig 4 and Fig 5). Accordingly, approximately 4% to 7% reductions in absolute mortality inequalities by ethnicity might be achieved by tax for deaths in women at 45–64 y of age at 30 to 40 y after the tax rises commence. By way of comparison, if all people stopped smoking in 2011, this resulted in similarly shaped graphs to those in Fig 4 and Fig 5, but with reductions of absolute and relative inequalities of a third and a quarter (respectively) for 45 to 64-y-old females.

**Fig 4. Projected percentage changes in ethnic inequalities in all-cause mortality rates for 10% increases in tobacco tax per annum from 2011 to 2031—Standardized rate differences (SRD) in mortality.** Rates are standardized to the WHO world population.

**Fig 5. Projected percentage changes in ethnic inequalities in all-cause mortality rates for 10% increases in tobacco tax per annum from 2011 to 2031—Standardized rate ratios (SRR; percentage change in “excess” SRR or SRR-1).** Rates are standardized to the WHO world population.

Table 3. Standardized all-cause mortality rates^†^ (per 100,000) projected to 2041 by sex, age, and ethnic groupings for tax and no tax to 2031 and ethnic inequality measures (SRDs [per 100,000] and SRRs).

|  |  | **2041 under BAU** | | | **2041 with Tax** | | | **Percentage Decrease Post-tax (95% Simulation Interval)** | | |
| --- | --- | --- | --- | --- | --- | --- | --- | --- | --- | --- |
| **Group** | **Age in 2041** | **Rate** | **SRD** | **SRR** | **Rate** | **SRD** | **SRR** | **Rate** | **SRD (Māori c.f. non-Māori)** | **SRR ^ (Māori c.f. non-Māori)** |
| *Men* |  |  |  |  |  |  |  |  |  |  |
| Māori | 45–64 | 716.9 | 424.0 | 2.448 | 705.2 | 414.3 | 2.424 | -1.62% (-2.37% to -1.04%) | -2.27% (-3.3% to -1.46%) | -1.59% (-2.3% to -1.04%) |
|  | 65–84 | 3187.8 | 1327.8 | 1.713 | 3157.0 | 1304.8 | 1.704 | -0.97% (-1.46% to -0.61%) | -1.74% (-2.6% to -1.11%) | -1.32% (-1.98% to -0.85%) |
|  | 85+ | 14648.8 | 2762.1 | 1.232 | 14617.3 | 2743.7 | 1.231 | -0.22% (-0.36% to -0.12%) | -0.67% (-1.19% to -0.35%) | -0.56% (-1.03% to -0.28%) |
|  | All 45+ | 1689.6 | 720.5 | 1.743 | 1672.2 | 707.0 | 1.732 | -1.02% (-1.52% to -0.65%) | -1.87% (-2.76% to -1.2%) | -1.48% (-2.17% to -0.96%) |
| Non-Māori | 45–64 | 292.9 |  |  | 290.9 |  |  | -0.69% (-1.04% to -0.43%) |  |  |
|  | 65–84 | 1860.0 |  |  | 1852.2 |  |  | -0.42% (-0.65% to -0.26%) |  |  |
|  | 85+ | 11886.7 |  |  | 11873.6 |  |  | -0.11% (-0.18% to -0.06%) |  |  |
|  | All 45+ | 969.1 |  |  | 965.3 |  |  | -0.4% (-0.61% to -0.24%) |  |  |
| *Women* |  |  |  |  |  |  |  |  |  |  |
| Māori | 45–64 | 545.0 | 343.1 | 2.698 | 528.0 | 327.7 | 2.634 | -3.12% (-4.56% to -2.02%) | -4.49% (-6.5% to -2.94%) | -3.72% (-5.34% to -2.46%) |
|  | 65–84 | 2888.2 | 1541.8 | 2.143 | 2843.1 | 1502.3 | 2.119 | -1.56% (-2.37% to -0.98%) | -2.56% (-3.87% to -1.63%) | -2.16% (-3.24% to -1.38%) |
|  | 85+ | 13054.9 | 2777.1 | 1.269 | 13005.1 | 2736.5 | 1.266 | -0.38% (-0.64% to -0.22%) | -1.47% (-2.51% to -0.83%) | -1.38% (-2.37% to -0.77%) |
|  | All 45+ | 1452.3 | 721.9 | 1.987 | 1426.9 | 699.4 | 1.960 | -1.75% (-2.61% to -1.12%) | -3.12% (-4.64% to -2.02%) | -2.74% (-4.05% to -1.77%) |
| Non-Māori | 45–64 | 201.9 |  |  | 200.3 |  |  | -0.8% (-1.24% to -0.49%) |  |  |
|  | 65–84 | 1346.4 |  |  | 1340.8 |  |  | -0.42% (-0.67% to -0.25%) |  |  |
|  | 85+ | 10277.8 |  |  | 10268.6 |  |  | -0.09% (-0.15% to -0.05%) |  |  |
|  | All 45+ | 730.4 |  |  | 727.5 |  |  | -0.39% (-0.62% to -0.24%) |  |  |
| *Sexes combined* |  |  |  |  |  |  |  |  |  |  |
| Māori | All 45+ | 1571.0 | 721.2 | 1.848 | 1549.6 | 703.2 | 1.830 | -1.36% (-2.02% to -0.87%) | -2.49% (-3.69% to -1.61%) | -2.11% (-3.1% to -1.37%) |
| Non-Māori | All 45+ | 849.8 |  |  | 846.4 |  |  | -0.39% (-0.61% to -0.24%) |  |  |

† Age-standardized using WHO world population mean over 4,000 model simulations.

^ Percentage difference in excess SRR (i.e., SRR – 1).

Scenario analyses (see S2 Table) suggested that higher taxes, up to 20% per annum, would lead to increased health gains by 81% (undiscounted QALYs: 499,000). Setting the Māori price elasticity at the same as non-Māori (compared to 20% higher) would reduce the QALYs gained for Māori by 14%. Halving the SDs around most parameters with uncertainty would roughly halve the uncertainty interval (UI) ranges for both QALYs gained and net savings, and doubling the SDs would roughly double the intervals but would not result in the 95% UI including either health loss or increased net costs. By setting all morbidity parameters in the model and the discount rate to zero, we estimated 259,000 life years saved for the 10% per annum tax increase from 2011 to 2031 compared to BAU.

# Discussion

**Main Results and Interpretation**

This modeling was able to quantify sizeable health gains and net health system cost savings arising from annual tax increases for 20 y (from 2011 to 2031)—consistent with the international modeling literature for one-off price/tax increases. Although tobacco tax increases begin to accrue immediate health gains and cost savings, the maximal health gains and cost savings from tobacco tax occurred decades into the future. This is because smoking prevalence is higher among younger age groups, the tobacco tax effect is greater among young people (i.e., higher price elasticity), and this younger cohort is decades away from their peak point of benefiting from reduced rates of NCDs.

Our finding of net health system cost savings from reducing tobacco consumption adds to the limited existing evidence, although some studies (Dutch in particular) tend to find that reducing tobacco consumption actually increases costs as people live longer. However, these studies also have a wider scope of what is included in health system costing that is subject to debate, e.g., housing and living costs of residential care settings (see S1 Text).

A unique aspect of this study was the detailed quantification of health inequality impacts from raising tobacco taxes. It is increasingly recognized that population-wide interventions to address risk factors for NCDs (e.g., high blood pressure, obesity, and smoking) tend to reduce health inequalities [[38](#_ENREF_38),[39](#_ENREF_39)]. Indeed, our findings were consistent with a “pro-health equity” benefit for Māori and consistent with the other (albeit less-quantified) literature around tobacco prices/taxes and equity (see S1 Text). Even so, we found that ongoing tobacco tax increases are no panacea for reducing health inequalities given that they achieve only a 2%–3% reduction in future mortality inequalities by ethnicity. Hence, many programs across multiple risk factors and diseases and upstream social determinants are needed to substantially reduce health inequalities.

**Generalizability of Findings**

In countries with higher smoking prevalence than New Zealand and/or less rapidly diminishing smoking prevalence trends under BAU, per capita health gains and cost savings would be greater from a program of regular tobacco tax increases. Indeed, our results for the Māori population with relatively high smoking rates (33% in 2013 [[16](#_ENREF_16)]) serve as an approximation for populations elsewhere with high tobacco prevalence and disease rates.

These health gains will probably differ in jurisdictions where the “nicotine market” includes electronic nicotine delivery systems (ENDS) (e.g., nicotine-containing electronic cigarettes). Initially, coexistence of (possibly cheaper) substitutes for smoked tobacco may increase the responsiveness to tax (i.e., increased price elasticity), increasing the effectiveness of tax. In the long run, though, the absolute health and cost effects of tax in the presence of ENDS are difficult to predict. On the one hand, and if well regulated, ENDS in and of themselves may drive the prevalence down quickly, leaving little marginal gain for additional taxes. On the other hand, and if poorly regulated, ENDS may keep the smoking prevalence high because of normalization and dual use, paradoxically making tax more effective (as there remains a large pool of smokers).

In jurisdictions where some of the tobacco tax revenue is “recycled” into other tobacco control activities (e.g., California), there could be a compounding beneficial effect. In contrast, the health gains might be less in jurisdictions where nontaxed smuggled tobacco is readily available or where the climate suits homegrown tobacco production. The cost savings to a government as a whole would also be less if jurisdictions had to spend more on enforcement around illegal sales and on border control to limit smuggling as the prices of legal (taxed) tobacco kept rising. Such costs could be funded from the additional tobacco tax revenue from higher tobacco taxes (which we have estimated for New Zealand in a separate analysis [[5](#_ENREF_5)]).

**Study Strengths and Limitations**

This modeling work benefited from rich local data (including country-specific relative risks for smoking-related mortality), heterogeneity by ethnic group for inequality analyses, allowing for decay of smoking effect post quitting among ex-smokers, and the quantification of timing of health and cost impacts into the future. The costing data we used were particularly detailed, including the cost of passing a new law to enable the program of tax increases and both for related and unrelated health costs (to capture the costs of people living longer as a result of not smoking). Such detail of cost allocation has rarely been achieved in other studies. Nevertheless, future improvements to the New Zealand costing data are pending (e.g., scaling to better match the national health accounts, incorporation of mental health and maternity data [although this should make little difference to tobacco-related costing], and inclusion of disability support payments). These improvements will probably increase the absolute dollar amounts presented in this paper but are unlikely to alter the relative distribution by sociodemographics and time.

In addition to pending improvements to costing data, another potential limitation of this study was the unchanging price elasticities at much higher tobacco prices. Although there is no strong evidence to inform this issue, we note that price elasticities for much more expensive drugs such as cocaine, heroin, and cannabis (range: -0.23 to -0.50) are similar to those for current day tobacco [[40](#_ENREF_40)]. We have also likely underestimated the health gains, as we do not include “spillover" benefits of declining tobacco use such as reduced exposure to secondhand smoke (still common in countries like New Zealand [[41](#_ENREF_41)]) and increased denormalization of smoking. Furthermore, we did not model the health benefits from reducing the number of cigarettes smoked per day by smokers due to tax effects on nonquitters (though this might be countered by more intensive smoking of each cigarette consumed—for which there is some New Zealand evidence for poorer smokers [[42](#_ENREF_42)]).

Finally, “true uncertainty” will be wider than that shown in our UIs, as there are many necessary structural assumptions for such long-run modeling forecasts. For example, we assume (as do other tobacco models) that the effect of tobacco tax on cessation rates is only experienced in the year of the tax increase. Also, a major global economic downturn could influence smoker behavior in various ways, including via reducing tobacco affordability or changes in the tobacco smuggling market.

**Possible Implications for Further Research and for Policy**

Additional country-specific research is warranted on price elasticity variation by age and social groups, given these parameters are particularly critical to the impact of tobacco tax increases. Similarly, how these elasticities might change because of cross-price elasticity effects via availability of electronic cigarettes/ENDS (as per Grace et al. [[43](#_ENREF_43)]) and via changes in smuggling levels warrants research. Also relevant is how tobacco tax increases might work in conjunction with other tobacco control policies (e.g., intensive mass media campaigns) and the joint impact of multiple NCD control interventions. In particular, there may be prohealth synergies between the impact of alcohol taxes and tobacco taxes [[44–46](#_ENREF_44)].

A broader societal perspective would also consider the economic benefit of preventing tobacco-related morbidity and mortality of working-age adults. Such a perspective could also encompass the issue of potential regressivity of increased tobacco taxes. That is, although increased tobacco taxes may be “pro-health equity” overall, they may have mixed effects in terms of increasing financial burden. Thus, for smokers who quit or cut down to the point of lower tobacco spending, there will be likely financial benefits, but for smokers who continue to smoke at the same level or higher, there will be increased risk of financial hardship from the higher taxes. Governments could ameliorate the latter with targeting of more intensive smoking cessation support to this group of smokers or reducing their overall financial hardship in other ways (e.g., improvements to employment opportunities, the minimum wage, or benefit payment levels). Of note, however, is that other New Zealand modeling work suggests that the likely health harm from tobacco-tax-related hardship is small compared to that from smoking itself [[47](#_ENREF_47)].

Based on this modeling work, to achieve more rapid health gains and cost savings, tobacco tax increases need to be complemented by other measures that achieve high cessation rates among middle- and older-age smokers (e.g., well-designed mass media campaigns [[48](#_ENREF_48)]). Taxation could also be combined with potentially novel strategies such as denicotinization of tobacco [[49](#_ENREF_49)], phasing down of tobacco retail outlets [[50](#_ENREF_50)], or other proposed endgame strategies [[51](#_ENREF_51)]. Countries taking an incremental approach to tobacco control might wish to select a mix of the most cost-effective interventions (possibly a mix of tax increases and enhanced legal controls rather than more costly mass media campaigns). However, countries with endgame goals might wish to apply all feasible interventions to ensure the goal is achieved on target—regardless of relative cost-effectiveness of specific interventions. Such a mix of interventions might actually result in unexpected synergies that more rapidly accelerate progress towards the endgame goal.

Thinking more widely in terms of NCD prevention and global targets [[2](#_ENREF_2)], in societies with low and steadily declining smoking prevalence, other interventions that affect nearly everyone (e.g., raising alcohol taxes and taxing unhealthy processed foods) may still have larger impacts on population health than tobacco control programs. Indeed, our own modeling work on salt reduction in the processed food supply would tend to suggest higher levels of health gain compared to tobacco taxation [[52](#_ENREF_52)]. Nevertheless, tobacco tax generates large health gains compared to many widely accepted prevention programs (e.g., new vaccination programs [[53](#_ENREF_53)]), should reduce health inequalities, and is probably an essential component of any package of interventions to reduce the NCD burden in both developed and developing countries.

In conclusion, this modeling work has suggested that ongoing tobacco tax increases deliver sizeable health gains and health sector cost savings and are likely to reduce health inequalities. However, if policy makers are to achieve more rapid reductions in the NCD burden and health inequalities, they need to complement tobacco tax increases with additional tobacco control interventions focused on cessation.

# References

1. Lim SS, Vos T, Flaxman AD, Danaei G, Shibuya K, Adair-Rohani H, et al. A comparative risk assessment of burden of disease and injury attributable to 67 risk factors and risk factor clusters in 21 regions, 1990-2010: a systematic analysis for the Global Burden of Disease Study 2010. Lancet. 2012;380(9859): 2224-2260.

2. Beaglehole R, Bonita R, Ezzati M, Alleyne G, Dain K, Kishore SP, et al. NCD Countdown 2025: accountability for the 25 x 25 NCD mortality reduction target. Lancet. 2014;384(9938): 105-107.

3. IARC. Effectiveness of tax and price policies for tobacco control. IARC handbooks of cancer prevention in tobacco control, Volume 14. Lyon: International Agency for Research on Cancer (IARC); 2011.

4. van Baal P, Meltzer D, Brouwer W. Future costs, fixed healthcare budgets, and the decision rules of cost-effectiveness analysis. Health Econ. 2014; E-pub ahead of print. doi: 10.1002/hec.3138.

5. Cobiac LJ, Ikeda T, Nghiem N, Blakely T, Wilson N. Modelling the implications of regular increases in tobacco taxation in the tobacco endgame. Tob control. 2015;24: e154–160

6. The Parliament of the Commonwealth of Australia. Excise tariff amendment (tobacco) bill 2014. Canberra: The Parliament of the Commonwealth of Australia. http://www.austlii.edu.au/au/legis/cth/bill_em/etab2014276/memo_0.html. Accessed 22 June 2014

7. Hanewinkel R, Isensee B. Five in a row - reactions of smokers to tobacco tax increases: population-based cross-sectional studies in Germany 2001-2006. Tob Control. 2007;16(1): 34-37.

8. Over EA, Feenstra TL, Hoogenveen RT, Droomers M, Uiters E, van Gelder BM. Tobacco control policies specified according to socioeconomic status: health disparities and cost-effectiveness. Nicotine Tob Res. 2014;16(6): 725-732.

9. Brown T, Platt S, Amos A. Equity impact of population-level interventions and policies to reduce smoking in adults: a systematic review. Drug Alcohol Depend. 2014;138: 7-16.

10. Brown T, Platt S, Amos A. Equity impact of interventions and policies to reduce smoking in youth: systematic review. Tob Control. 2014; 23:e2 e98-e105

11. Ministry of Social Affairs and Health. Roadmap to a tobacco-free Finland: Action plan on tobacco control. Helsinki: Ministry of Social Affairs and Health; 2014.

12. Department of Health. Tobacco Free Ireland - report of the tobacco policy review group. Dublin: Department of Health; 2013.

13. The Scottish Government. Creating a tobacco-free generation: A tobacco control strategy for Scotland. Edinburgh: The Scottish Government; 2013.

14. World Health Organization Western Pacific Region. Apia communiqué on healthy islands, NCDs and the post-2015 development agenda - tenth pacific health ministers meeting 2013. <http://www.wpro.who.int/southpacific/pic_meeting/2013/meeting_outcomes/10th_PHMM_Apia_Commnique.pdf>. Accessed 5 May 2015.

15. New Zealand Parliament. Government response to the report of the Māori Affairs Select Committee on its inquiry into the tobacco industry in Aotearoa and the consequences of tobacco use for Māori (final response). Wellington: New Zealand Parliament; 2011.

16. van der Deen FS, Ikeda T, Cobiac L, Wilson N, Blakely T. Projecting future smoking prevalence to 2025 and beyond in New Zealand using smoking prevalence data from the 2013 census. N Z Med J. 2014;127(1406): 71-79.

17. Tobias M, Blakely T, Matheson D, Rasanathan K, Atkinson J. Changing trends in indigenous inequalities in mortality: lessons from New Zealand. Int J Epidemiol. 2009;38(6): 1711-1722.

18. Blakely T, Fawcett J, Hunt D, Wilson N. What is the contribution of smoking and socioeconomic position to ethnic inequalities in mortality in New Zealand? Lancet. 2006;368(9529): 44-52.

19. Blakely T, Thomson G, Wilson N, Edwards R, Gifford H. The Māori Affairs Select Committee Inquiry and the road to a smokefree Aotearoa. N Z Med J. 2010;123(1326): 7-18.

20. Edwards R, Hoek J, van der Deen FS. Smokefree 2025 - use of mass media in New Zealand lacks alignment with evidence and needs. Aust N Z J Public Health. 2014;38(4): 395-396.

21. Blakely T, Barendregt JJ, Foster RH, Hill S, Atkinson J, Sarfati D, et al. The association of active smoking with multiple cancers: national census-cancer registry cohorts with quantitative bias analysis. CCC. 2013;24: 1243–1255.

22. Barendregt J, Oortmarssen GJ, Vos T, Murray CJL. A generic model for the assessment of disease epidemiology: the computational basis of DisMod II. Popul Health Metr. 2003;1(1): 4.

23. Ministry of Health. Health Loss in New Zealand: A report from the New Zealand Burden of Diseases, Injuries and Risk Factors Study, 2006–2016. Wellington: Ministry of Health; 2013.

24. Woodward A, Blakely T. The healthy country? A history of life and death in New Zealand. Auckland: University of Auckland Press; 2014.

25. Costilla R, Atkinson J, Blakely T. Incorporating ethnic and deprivation variation to cancer incidence estimates over 2006-2026 for ABC-CBA. Wellington: Department of Public Health, University of Otago, Wellington; 2011.

26. Blakely T, Costilla R, Soeberg M. Cancer excess mortality rates over 2006-2026 for ABC-CBA. Wellington: Department of Public Health, University of Otago, Wellington; 2012.

27. Wilson N, Nghiem N, Foster R, Cobiac L, Blakely T. Estimating the cost of new public health legislation. Bull World Health Organ. 2012;90: 532-539.

28. Ikeda T, Cobiac L, Wilson N, Carter K, Blakely T. What will it take to get to under 5% smoking prevalence by 2025? Modelling in a country with a smokefree goal. Tob Control. 2015;24: 139-145.

29. Verguet S, Gauvreau CL, Mishra S, MacLennan M, Murphy SM, Brouwer ED, et al. The consequences of tobacco tax on household health and finances in rich and poor smokers in China: an extended cost-effectiveness analysis. Lancet Glob Health. 2015;3(4): e206-16.

30. Grace RC, Kivell BM, Laugesen M. Predicting decreases in smoking with a cigarette purchase task: evidence from an excise tax rise in New Zealand. Tob Control. 2014. doi: 10.1136/tobaccocontrol-2014-051594

31. Ni Mhurchu C, Eyles H, Schilling C, Yang Q, Kaye-Blake W, Genc M, et al. Food prices and consumer demand: differences across income levels and ethnic groups. PloS ONE. 2013;8(10): e75934.

32. Hunt D, Blakely T, Woodward A, Wilson N. The smoking-mortality association varies over time and by ethnicity in New Zealand. Int J Epidemiol. 2005;34: 1020-1028.

33. Thun M, Apicella L, Henley S. Smoking vs other risk factors as the cause of smoking-attributable deaths: confounding in the courtroom. JAMA. 2000;284(6): 706-712.

34. Hoogenveen R, van Baal P, Boshuizen H, Feenstra T. Dynamic effects of smoking cessation on disease incidence, mortality and quality of life: The role of time since cessation. Cost Eff Resour Alloc. 2008;6: 1.

35. Salomon JA, Vos T, Hogan DR, Gagnon M, Naghavi M, Mokdad A, et al. Common values in assessing health outcomes from disease and injury: disability weights measurement study for the Global Burden of Disease Study 2010. Lancet. 2012;380(9859): 2129-2143.

36. van Baal PHM, Feenstra TL, Polder JJ, Hoogenveen RT, Brouwer WBF. Economic evaluation and the postponement of health care costs. Health Econ. 2011;20(4): 432-445.

37. McLeod M, Blakely T, Kvizhinadze G, Harris R. Why equal treatment is not always equitable: the impact of existing ethnic health inequalities in cost-effectiveness modeling. Popul Health Metr. 2014;12(1): 15.

38. Kivimäki M, Shipley MJ, Ferrie JE, Singh-Manoux A, Batty GD, Chandola T, et al. Best-practice interventions to reduce socioeconomic inequalities of coronary heart disease mortality in UK: a prospective occupational cohort study. Lancet. 2008;372(9650): 1648-1654.

39. Capewell S, Graham H. Will Cardiovascular Disease Prevention Widen Health Inequalities? PLoS Med. 2010;7(8): e1000320.

40. Gallet CA. Can price get the monkey off our back? A meta-analysis of illicit drug demand. Health economics. 2014;23(1): 55-68.

41. Wilson N, Edwards R, Parry R. A persisting secondhand smoke hazard in urban public places: results from fine particulate (PM2.5) air sampling. N Z Med J. 2011;124(1330): 34-47.

42. Thomson G, Wilson N, Bushell L, Al Matar W, Ball B, Chiu J, et al. Butt lengths differ by area deprivation level: a field study to explore intensive smoking. Nicotine Tob Res. 2008;10(5): 927-931.

43. Grace RC, Kivell BM, Laugesen M. Estimating cross-price elasticity of e-cigarettes using a simulated demand procedure. Nicotine Tob Res. 2015;17(5): 592-598.

44. Jimenez S, Labeaga JM. Is it possible to reduce tobacco consumption via alcohol taxation? Health Econ. 1994;3(4): 231-241.

45. Lee JM, Chen MG, Hwang TC, Yeh CY. Effect of cigarette taxes on the consumption of cigarettes, alcohol, tea and coffee in Taiwan. Public Health. 2010;124(8): 429-436.

46. Young-Wolff KC, Kasza KA, Hyland AJ, McKee SA. Increased cigarette tax is associated with reductions in alcohol consumption in a longitudinal U.S. sample. Alcohol Clin Exp Res. 2014;38(1): 241-248.

47. Wilson N, Thomson G, Tobias M, Blakely T. How much downside? Quantifying the relative harm from tobacco taxation. J Epidemiol Comm Health. 2004;58(6): 451-454.

48. Bala MM, Strzeszynski L, Topor-Madry R, Cahill K. Mass media interventions for smoking cessation in adults. Cochrane Database Syst Rev 6. 2013;6: CD004704.

49. Donny EC, Hatsukami DK, Benowitz NL, Sved AF, Tidey JW, Cassidy RN. Reduced nicotine product standards for combustible tobacco: Building an empirical basis for effective regulation. Prev Med. 2014;67: 17-22.

50. Pearson AL, van der Deen FS, Wilson N, Cobiac L, Blakely T. Theoretical impacts of a range of major tobacco retail outlet reduction interventions: modelling results in a country with a smokefree nation goal. Tob Control. 2014;24: e32-338.

51. Warner KE. An endgame for tobacco? Tob Control. 2013;22: i3-i5.

52. Nghiem N, Blakely T, Cobiac L, Pearson A, Wilson N. Health and economic impacts of eight different dietary salt reduction interventions. PloS ONE. 2015;10(4): e0123915.

53. Blakely T, Kvizhinadze G, Karvonen T, Pearson AL, Smith M, Wilson N. Cost-effectiveness and equity impacts of three HPV vaccination programmes for school-aged girls in New Zealand. Vaccine. 2014;32: 2645–2656.

**Editors’ Summary**

**Background.**

Worldwide, approximately 21% of the population aged 15 and above smoke cigarettes and other tobacco products. However, tobacco is a killer. Half of all tobacco users die because of cancer, lung disease, heart disease, or another tobacco-related disease. Tobacco caused 100 million deaths in the 20th century; if current trends continue, it could cause a many more deaths in the 21st century. In response to this global tobacco epidemic, individual countries are considering how to reduce or even end tobacco consumption (the “tobacco endgame”), and international bodies have drawn up various tobacco control directives. The World Health Organization, for example, has developed an international instrument for tobacco control called the Framework Convention on Tobacco Control (FCTC). Countries that are party to the FCTC agree to implement a wide range of measures designed to reduce tobacco use. For example, they agree to implement bans on tobacco advertising, promotion, and sponsorship and to protect people from tobacco smoke exposure in public spaces and indoor workplaces.

**Why Was This Study Done?**

An important tobacco control measure is the implementation of taxation policies aimed at reducing tobacco consumption, promoting cessation, and preventing young people from taking up smoking. But less is known about the impact on population health, health inequality (higher rates of illness and death in specific parts of the population), and health system costs of annual tobacco tax increases, a strategy that is being used or considered by many countries. Here, the researchers estimate the impact of annual tobacco tax increases compared with no tax increases in New Zealand using a mathematical approach called multistate life table modeling. In New Zealand, a country that collects data on disease incidence and mortality by sociodemographic status and has implemented annual tobacco taxation increases since 2010, there are marked health inequalities between indigenous Māori (15% of the population) and non-Māori residents. Smoking is a major cause of these existing health inequalities (as in many other countries)—33% of Māori smoke compared to 14% of New Zealand Europeans. A life table shows, for each age, the probability that a person of that age will die before his or her next birthday.

**What Did the Researchers Do and Find?**

The researchers estimated quality-adjusted life-years (QALYs; a measure of disease burden that includes both the quantity and quality of life lived) gained and net health system costs over the remaining life of New Zealand’s 2011 population exposed to annual 10% tobacco tax increases or to no tax increases by modeling 16 tobacco-related diseases in parallel using national data on all-cause mortality and morbidity (illness). Compared to the 2011 population exposed to no tax increases, 260,000 QALYs were gained among the population exposed to annual tax increases from 2011 to 2031 and followed up for the remainder of their lives. The net health system cost savings associated with this intervention were estimated at US$2,550 million over the remainder of the 2011 population’s life. Both the health gains and cost savings began to accrue immediately but took five decades to peak. The QALY gains per capita associated with annual tobacco tax increases were 3.7-fold higher for Māori than for non-Māori because of higher smoking levels and price sensitivity among Māori (the tobacco purchasing behavior of Māori is affected more by the price of tobacco than non-Māori because they have less disposable income). Finally, projected health inequalities measured by the difference in mortality rates among Māori and non-Māori aged 45+ y were 2.3% lower in 2041 with ongoing tax rises than with no tax rises.

**What Do These Findings Mean?**

These findings suggest that in New Zealand (and probably in other similar populations), ongoing tobacco tax increases should deliver sizeable health gains and health system cost savings and should modestly reduce health inequalities. Notably, the health gains and cost savings will not peak for several decades because smoking is more common among younger age groups and the tobacco tax effect is greater among young people (who have limited disposable income) but young people do not benefit maximally from reduced rates of tobacco-related diseases for many decades. As with all modeling studies, the accuracy of these findings depends on the assumptions built into the model and the data fed into it. Overall, however, these findings support the use of annual increases of tobacco tax as a way to improve population health, save health system costs, and reduce health inequalities but suggest that policy makers will need to introduce other tobacco control interventions focused on smoking cessation among middle-age to older smokers if they want to achieve more rapid reductions in tobacco-related diseases and health inequalities.

**Additional Information.**

This list of resources contains links that can be accessed when viewing the PDF on a device or via the online version of the article at <http://dx.doi.org/10.1371/journal.pmed.1001856>.

- The World Health Organization provides information about the dangers of [tobacco](http://who.int/topics/tobacco/en/index.html) (in several languages), about [FCTC](http://www.who.int/fctc/en/), and about [tobacco control economics](http://www.who.int/tobacco/economics/en/); its [2013 report on the global tobacco epidemic](http://who.int/tobacco/global_report/2013/en/) is available
- The United States Centers for Disease Control and Prevention provides detailed information about all aspects of [smoking and tobacco use](http://www.cdc.gov/tobacco/)
- The United Kingdom National Health Services website provides information about the [health risks associated with smoking](http://www.nhs.uk/chq/Pages/2344.aspx?CategoryID=53)
- The UK campaigning public health not-for profit organization [Action on Smoking and Health (ASH)](http://ash.org.uk/) provides detailed information on [tobacco taxation](http://ash.org.uk/pathfinder/price-and-tax)
- Information about [tobacco control in New Zealand](http://www.health.govt.nz/our-work/preventative-health-wellness/tobacco-control) is available
- [SmokeFree](http://smokefree.nhs.uk/), a website provided by the UK National Health Service, offers advice on quitting smoking and includes personal stories from people who have stopped smoking
- [Smokefree.gov](http://www.smokefree.gov/), from the US National Cancer Institute, offers online tools and resources to help people quit smoking

# Supporting Information

**S1 Fig. Projected QALYs gained (thousands) and net health system costs saved (millions) by year for 10% per annum tax increase to 2031, by age cohort in 2011*.** * Same as Fig S4 in S2 Text.

**S1 Table. QALYs gained by disease from a 10% per annum increase in tobacco tax (from 2011 to 2031), among the New Zealand population alive in 2011.**

**S2 Table. Scenario analyses about QALY and life year gains and health system cost savings for tobacco tax compared to BAU.**

**S3 Table. QALYs gained and health system costs averted, with uncertainty, from a 10% per annum increase in tobacco tax (from 2011 to 2031), among the New Zealand population alive in 2011 (the same as Table 2 in the main manuscript but showing uncertainty).**

**S1 Text. Literature reviews’ methods and results.**

**S2 Text. Supporting information: Model, supplementary results, DISMOD II example, validation, epidemiological inputs, and health system costs.**
