# Supplementary figures and images for: Correction: Health, Health Inequality, and Cost Impacts of Annual Increases in Tobacco Tax: Multistate Life Table Modeling in New Zealand
Source: PLoS Med. 2016 Dec 22;13(12):e1002211. doi: 10.1371/journal.pmed.1002211 (PMC5179055; doi:10.1371/journal.pmed.1002211)

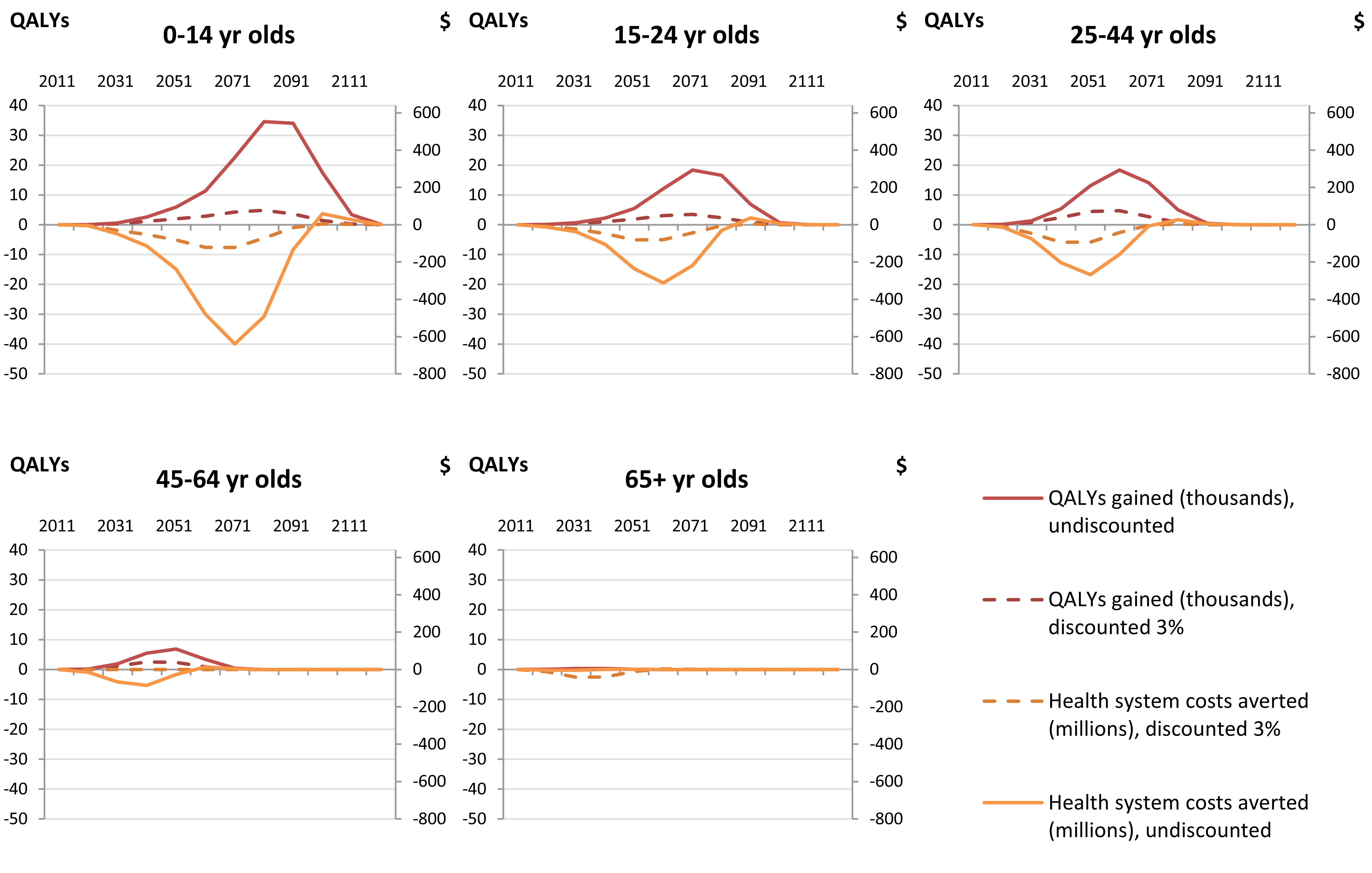

Supplement: S1 Fig — (TIF) [file pmed.1002211.s002.tif]
