# Supplementary material for: Correction: Health, Health Inequality, and Cost Impacts of Annual Increases in Tobacco Tax: Multistate Life Table Modeling in New Zealand
Source: PLoS Med. 2016 Dec 22;13(12):e1002211. doi: 10.1371/journal.pmed.1002211 (PMC5179055; doi:10.1371/journal.pmed.1002211)
Supplement: S2 Text — (DOC) [file pmed.1002211.s003.doc]

# S2 Text – Supporting Information: Model, supplementary results, DISMOD II example, validation, epidemiological inputs, and health system costs

Supporting Information for ***Health, Health Inequality, and Cost Impacts of Annual Increases in Tobacco Tax: Multistate Life Table Modeling in New Zealand***

Contents

[Model 2](#__RefHeading___Toc421711855)

[Model overview 2](#__RefHeading___Toc421711856)

[Design, purpose and specification 2](#__RefHeading___Toc421711857)

[Life table analysis 2](#__RefHeading___Toc421711858)

[Tobacco-related disease models 4](#__RefHeading___Toc421711859)

[Diseases included in BODE^3^ tobacco multistate life table model 5](#__RefHeading___Toc421711860)

[Model: Baseline specification and parameters 6](#__RefHeading___Toc421711861)

[Background population inputs 6](#__RefHeading___Toc421711862)

[Data sources, processing, DISMOD, and inputs to BODE^3^ multistate life table models 6](#__RefHeading___Toc421711863)

[Final processing of incidence and prevalence estimates 10](#__RefHeading___Toc421711864)

[Future disease trends (incidence, remission and case-fatality) 10](#__RefHeading___Toc421711865)

[Disease cost inputs 11](#__RefHeading___Toc421711866)

[Validation 12](#__RefHeading___Toc421711867)

[Model: Intervention specification and parameters 13](#__RefHeading___Toc421711868)

[Health impact of changing exposure to tobacco smoke 13](#__RefHeading___Toc421711869)

[Relative risks of smoking-related diseases 13](#__RefHeading___Toc421711870)

[Smoking prevalence 15](#__RefHeading___Toc421711871)

[Model: Analysis 16](#__RefHeading___Toc421711872)

[Supplementary results: figures and tables to the main manuscript 17](#__RefHeading___Toc421711873)

[References 27](#__RefHeading___Toc421711874)

[Appendix A: DISMOD II example for lung cancer 28](#__RefHeading___Toc421711875)

[Appendix B: Validation 32](#__RefHeading___Toc421711876)

[Appendix C: Epidemiological inputs 36](#__RefHeading___Toc421711877)

[Appendix D: Health system costs 60](#__RefHeading___Toc421711878)

# Model

## Model overview

### Design, purpose and specification

A proportional multistate life table model was developed in Excel to evaluate the effectiveness of interventions to reduce tobacco smoking and related diseases, and additionally to estimate costs and cost-effectiveness. The model evaluates intervention effect, cost and cost-effectiveness over the remaining lifetime of the New Zealand population alive in baseline year 2011, using information on baseline and projected future business-as-usual (BAU) trends in tobacco and disease epidemiology, and health system costs, overlaid with tobacco intervention costs and effects.

### Life table analysis

The difference in years of life lived between a New Zealand population that smokes tobacco at projected BAU trends, and an identical population that receives the intervention (therefore altering future projected tobacco smoking rates and thence tobacco-related disease rates), is calculated in a proportional multistate life table (Fig. S1). The population was divided into five-year age group cohorts (from age 0 to age 105+), and simulated in the life table until 110 years of age.

**Prevalent years lived with disability**Years of life lived by each cohort are adjusted at each age for time spent in poor health (‘disability’) due to disease or injury, using disability estimates from the New Zealand Burden of Disease (NZBDS)study which in turn are derived from the Global Burden of Disease (GBD) Study 2010.In the main life table, morbidity in the BAU scenario at each age is the total years of life lived with disability (so called prevalent YLDs, or pYLDs) from the 2006 NZBDS (scaled down to adjust for overlapping morbidities), divided by the total number of people in the population. (These YLDs were first projected forward to 2011 using expected trends in disease incidence, remission and case-fatality (see later in this Appendix)).

In each disease-specific life table, morbidity is assigned by using the corresponding disease’s YLDs from the NZBDS divided by the population size, or so-called ‘disability rates’. These disability rates can be loosely thought of an equivalent to disabilities for the disease in question, by age. For example, if in a given age group there are an estimated 50 pYLDs and 200 prevalent cases, the DR is 0.25. This DR is assigned to that disease state in the BODE^3^ tobacco multistate life table model, and results in a 25% loss in quality of life for any year lived with that disease. These pYLDs are specific to each disease group, sex and age.

**Quality-Adjusted Life-Years (QALYs)**Previous BODE^3^ modelingtermed health gain as ‘disability-adjusted life-years (DALYs) averted’. We currently refer to them as QALYs gained. The disability weighting (in this case DRs, which in term stem from DWs applied in the BDS itself) assigned is just one variant of health status valuation (HSVs); QALYs use a variety of HSVs (e.g. those from EQ5D, etc). Furthermore, DALYs in a Burden of Disease Study use an external or reference life table (to generate a health gap or loss measure); in this multistate life table, the DALYs averted are at the incremental margin for the 2011 New Zealand population, the same concept and method as used for QALYs. The only conceptual difference between the QALYs we calculate and the various QALYs presented in much other research, is the HSV metric. In other cost-utility analyses the source of HSV is likely to vary between studies (arguably to fit the population’s preference, but more usually due to the pragmatics of different questionnaires used) whereas our QALYs are derived from one very large and coherent set of disability weights calculated in the GBD2010 from multi-country surveys. We do not claim that the HSV in our QALY is ‘better’ than that used in other QALY estimates – there is genuine uncertainty in all HSVs.

The QALY metric captures health gain (assuming the intervention is beneficial) that arises from a mix of change in years of life and quality of each year of life. Usually a gain in QALYs (in prevention interventions at least) is due to a gain in life years lived (with or without change in quality of life). Note, however, that it is possible to achieve QALY gains with a reduction in life years lived (but very good improvements in quality of life), or with an increase in life years gained that is greater than any ‘penalty’ from living in lower quality of life.

Fig. S1 gives a schematic of how the differences in health adjusted life expectancy (and hence differences in DALYs averted or QALYs gained) is calculated. In descriptive terms, imagine the cohort of the entire New Zealand population with no model-specific diseases – that is, all the living and dying occurs in the ‘overall’ life table part of the model. The cohort simply lives the remaining years expected given defined overall mortality and morbidity rates.

Second, imagine we now specify two separate disease processes – say CHD and stroke. These disease processes actually have their own separate life tables acting on a ‘subsidiary’ cohort of people with that disease’s incidence, case-fatality and morbidity parameters. Note that the proportion of the cohort with CHD or stroke (and alive ‘in’ these disease process states) are also still alive in the main life table as well – hence the term ‘*multistate’* life table model as proportions of the cohort can be in multiple states simultaneously.

Third, imagine we now want to model the impact on health adjusted life expectancy (and therefore QALYs) for a preventive intervention that reduces the incidence of stroke and CHD. The model structure of how this intervention works will vary depending on the intervention type (e.g. price changes for tobacco, mass media campaigns for smoking cessation, etc), but the modeling process for that effect is the same for all types of interventions. Namely, one uses standard population impact fraction (PIF) estimations. PIFs are the percentage change in disease incidence for a given intervention acting through a risk factor with (usually) a given relative risk. Assume the intervention lowers the incidence of selected (e.g. tobacco-related diseases), which leads to a corresponding increase in health adjusted life years by:

1. Lowering the incidence of the tobacco-related diseases in the ‘intervention’ scenario.
2. Which in turn results in lowered mortality and morbidity from each tobacco-related disease.
3. Which is then subtracted from the total mortality and morbidity rates in the main life table (repeated for every tobacco-related disease modeled in parallel), to give a post-intervention total health adjusted life expectancy and a post-intervention expected total QALYs remaining for the population.
4. The QALYs gained due to the intervention is, simply, the total post-intervention QALYs expected for the population minus the total pre-intervention or BAU QALYs.

Fig.S1: Schematic of a proportional multistate life table, showing the interaction between disease parameters and life table parameters, where x is age, i is incidence, p is prevalence, m is mortality, w is disability-adjustment (or health status valuation), q is probability of dying, l is number of survivors, L is life years, Lw is health adjusted life expectancy, and where ‘-‘ denotes a parameter that specifically excludes modeled diseases, and ‘+’ denotes a parameter for all diseases (i.e. including modeled diseases) .

**Health system costs**Just as proportions of the cohort ‘alive’ in the overall and disease process are rewarded with additional QALYs for each annual cycle they live, so too can health system costs be ‘rewarded’. In the BODE^3^multistate life table models, we have five types of health system cost:

- Main life table:
  1. Annual cost to the New Zealand health system for being alive for a given sex and age, and *not* in the last six months of life and *not* concurrently alive with one of the modeled diseases (i.e. tobacco-related). All members of the cohort are assigned this cost; it is the base cost.
  2. Excess cost to A for being in the last six months of life if dying of a disease *other than* one of the modeled diseases (i.e. dying of a non-tobacco-related disease).
- Disease process life tables:

1. Excess cost to A for being in first year of diagnosis of a tobacco-related disease.
2. Excess cost to A for being alive with a tobacco-related disease, and neither in the first year of diagnosis nor in the last six months of life if dying of that disease.
3. Excess cost to A for being in the last six months of life if dying of a tobacco-related disease.

The derivation of these five costs is described later in this Appendix.

Finally, because the modeling focus has been on disease prevention, we have described interventions which reduce future disease incidence. It is also possible to parameterize interventions that change disease morbidity or case-fatality – but they must be undertaken carefully given how morbidity and case-fatality rates are derived for the model, and they are beyond the scope of this Appendix.

### Tobacco-related disease models

Tobacco smoking has been linked to increased incidence of various cancers (e.g. lung, head and neck), cardiovascular diseases (e.g. coronary heart disease (CHD), stroke), and respiratory conditions (e.g. chronic obstructive pulmonary disease (COPD), lower respiratory tract infection (LRTI)). These diseases were modeled explicitly using rates of disease incidence, disease remission, mortality from the disease (or case-fatality), and a background mortality rate (from both the diseased and healthy states) (Fig. S2). Note that this model occurs in each separate or parallel disease-specific life table, and generates differences in mortality and morbidity (and prevalence and cost) rates between the BAU and intervention scenarios, and these differences in rates are then feedback into the main life table (Fig. S1).

Healthy

Diseased

Dead

(disease)

Dead

(other)

Mortality

(other)

Remission

Incidence

Case-fatality

Mortality (other)

Fig.S2: Each disease was modeled with four states (healthy, diseased, dead from the disease, and dead from all other causes) and transition hazards between states of incidence, remission, case-fatality and mortality from all other causes.

### Diseases included in BODE^3^ tobacco multistate life table model

The following table (Table S1) includes all the diseases included in the BODE^3^ tobacco multistate life table model. Note that an example (lung cancer) of the specific details of data sources and both pre-DISMOD processing and DISMOD manipulation prior to inclusion in the BODE^3^ tobacco multistate life table model are included in the Appendix A.

There are many diseases associated with smoking, with varying evidence in terms of the contribution of tobacco use to their incidence. In principle, we prioritised inclusion in the model for: 1) diseases which were included as smoking-related in the New Zealand Burden ofDisease Study; 2) cancers that were found to have a significant association with smoking in the New Zealand CancerTrends study; and 3) other smoking-related diseases that contributed to at least 0.5% of the total disease burden in New Zealand in 2006 (as measured in DALYs in the NZBDS). Diseases needed to meet criteria 1 and 3 or criteria 2 to be included in the model. Most of the included diseases were chronic diseases (e.g. various cancers) with the exception of acute lower respiratory tract infections.

Some diseases with relatively high relative risks (RRs) were excluded such as age-related macular degeneration (ARMD) and peripheral vascular disease, as these each make up <0.5% of the total disease burden in New Zealand (and therefore would contribute negligible health gains in a smoking intervention model). Also, data availability and reliability were poor for the following conditions: gum disease, impotence, female infertility, hip fracture risk, fire-related injuries and other injuries, and mental health effects such as depression, anxiety, and for the psychological state of being “addicted” to tobacco. We did not include diseases from second-hand smoke and smoking in pregnancy as this was deemed too complex for our macro-simulation modeling – thus there is an inherent bias in our modeling to underestimate health gains from tobacco reduction (albeit probably slightly). Regarding female breast cancer IARC states “limited” evidence for the association with smoking, therefore we did not include it. Last, we did not include “ovary mucinous” cancer, as this was not included in the NZBDS and would be a minor contributor to total health gains (even though IARC states that there is sufficient evidence for an association with smoking). Also of note, we included three diseases for which smoking appears to have protective effects: melanoma, thyroid cancer and endometrial cancer (based on the CancerTrends study).

Table S1: Tobacco Smoking Related Diseases included in BODE^3^ tobacco multistate life table model

| **NZBDS codes** | **Condition** |
| --- | --- |
| ***Cancers*** |  |
| C09 | Lung cancer |
| C01 | Head & neck cancer |
| C02 | Esophageal cancer |
| C03 | Stomach cancer |
| C06 | Liver cancer |
| C08 | Pancreatic cancer |
| C13 | Cervical cancer |
| C18 | Bladder cancer |
| C19 | Kidney cancer |
| C14 | Endometrial cancer |
| C10 | Melanoma |
| C21 | Thyroid cancer |
| ***Respiratory disease*** |  |
| I02 | Chronic obstructive pulmonary disease |
| A08 | Lower respiratory tract infection |
| ***Cardiovascular disease*** |  |
| E01 | Coronary heart disease which includes “congestive heart failure” |
| E10 | Stroke |

## Model: Baseline specification and parameters

### Background population inputs

The following population parameters were included: 1) population size; 2) total pYLDs; and 3) total mortality rates, all by 5-year age groups for each sex and ethnicity. Population counts were compiled using Statistics New Zealand 2011 estimates. Total pYLDs were calculated using the total (corrected for multiple morbidity) YLDs for all diseases in the NZBDS divided by the total population in New Zealand for each age, sex and ethnicity group. Population mortality rates were calculated from data from the Statistics New Zealand life tables for 2010-2012. The trend in future total mortality rates was a function of trends set for each disease, plus trends in the mortality for diseases not included in the model. For example, imagine a model with only two diseases – CHD and stroke. Assume the future annual percentage change (APC) in incidence for both these diseases is -2%, and for case-fatality is also -2%. Then, once the model is equilibrated, the APC for mortality from CHD and stroke will be -4%. Assume that for a given sex, ethnic and age group the CHD deaths in 2011 made up 15% of all death, and stroke 5% of all deaths. Assume that the other diseases had an APC in mortality rate of 1.75% for non-Māori and 2.25% for Māori (consistent with long-run trends). The estimated APC for non-Māori all-cause mortality in the first year is a weighted average: 15% × -4% + 5% × -4% + 80%× -1.75% = -2.2%. This APC is then (slightly) adjusted for each year out into the future by changing proportionate mortality. Such estimated APC in all-cause mortality (as the input to the BAU part of the overall life table) were included out to 2026, then 0% APC was assumed.

### Data sources, processing, DISMOD, and inputs to BODE^3^ multistate life table models

The basic steps for generating disease inputs for BODE^3^ tobacco multistate life table model were: 1) data compilation; 2) preliminary processing of the data; and 3) DISMOD II estimation of epidemiologic parameters.

**Step 1)**: Data for these diseases were compiled from various sources (see Table S2).

**Step 2)**: Some parameters were further processed to give ‘best’ (pre-DISMOD) estimates for 2011. For example, data on prevalence for less common diseases were compiled and then regression-smoothed prior to inserting into DISMOD II. Readers can refer to Appendix A for a step by step description of data compilation and processing in DISMOD II for one example disease, lung cancer. (Similar documentation for all other diseases is available from the authors on request.)All parameters were generated by 5-year age groups by sex and ethnicity (Māori/Non-Māori), except cervical and endometrial cancers which were only compiled for women.

**Step 3)**: These parameters were then inputted to DISMOD II, separately by sex and ethnicity, to generate an ‘epidemiologically consistent’ set of parameters. For example, if the prevalence estimate was too low given what is known about incidence and case-fatality from the disease (and background ‘competing’ mortality), DISMOD II outputs values that are epidemiologically / mathematically consistent, allowing the user to ‘weight’ the inputs. For cancer incidence, full weighting (setting at “100%”) was given, as it was the most reliable parameter. Typically, mortality was also given full weighting and prevalence was given a 50% weighting (for diseases-specific weighting information, see README files for the disease of interest available upon request from the authors, and for lung cancer (only) in the Appendix A). The outputs from DISMOD II were then used to populate the BODE^3^ multistate life table model.

Table S2: Tobacco-related disease data sources and processing notes for those disease variables subsequently estimated in DISMOD II

| **Disease** | **Incidence** | **Prevalence** | **Disease-specific mortality** | **Case-fatality rate** | **Remission** |
| --- | --- | --- | --- | --- | --- |
| Lung cancer | BODE^3^ estimates^ | NZBDS, 5-year look back | NZBDS | Generated using equations**†** | Generated using equations**†** |
| Head & neck cancer | BODE^3^ estimates^ | NZBDS, 5-year look back, regression smoothed | NZBDS, regression smoothed | Generated using equations**†** | Generated using equations**†** |
| Esophageal cancer | BODE^3^ estimates^ | NZBDS, 5-year look back, regression smoothed | NZBDS, regression smoothed | Generated using equations**†** | Generated using equations**†** |
| Stomach cancer | BODE^3^ estimates^ | NZBDS, 5-year look back | NZBDS | Generated using equations**†** | Generated using equations**†** |
| Liver cancer | BODE^3^ estimates^ | NZBDS, 5-year look back | NZBDS | Generated using equations**†** | Generated using equations**†** |
| Pancreatic cancer | BODE^3^ estimates^ | NZBDS, 5-year look back, regression smoothed | NZBDS, regression smoothed | Generated using equations**†** | Generated using equations**†** |
| Cervical cancer | BODE^3^ estimates^ | NZBDS, 5-year look back, regression smoothed | NZBDS | Generated using equations**†** | Generated using equations**†** |
| Bladder cancer | BODE^3^ estimates^ | NZBDS, 5-year look back | NZBDS | Generated using equations**†** | Generated using equations**†** |
| Kidney cancer | BODE^3^ estimates^ | NZBDS, 5-year look back, regression smoothed | NZBDS, regression smoothed | Generated using equations**†** | Generated using equations**†** |
| Leukemia | BODE^3^ estimates^ | NZBDS, 5-year look back, regression smoothed for females only | NZBDS, regression smoothed | Generated using equations**†** | Generated using equations**†** |
| Endometrial cancer | BODE^3^ estimates^ | NZBDS, 5-year look back | NZBDS | Generated using equations**†** | Generated using equations**†** |
| Melanoma | BODE^3^ estimates^ | NZBDS, 5-year look back, regression smoothed | NZBDS, regression smoothed | Generated using equations**†** | Generated using equations**†** |
| Thyroid cancer | BODE^3^ estimates^ | NZBDS, 5-year look back, regression smoothed | NZBDS, regression smoothed | Generated using equations**†** | Generated using equations**†** |
| COPD | Initial DISMOD output, regression smoothed | NZBDS, 10-year look back | NZBDS | For Māori only: Initial DISMOD output and specified for <20 years | Set to 0 |
| CHD | HealthTracker, regression smoothed for Māori only | NZBDS, 10-year look back | Ministry of Health* |  | Set to 0 |
| Stroke | HealthTracker | HealthTracker | Ministry of Health* |  | Set to 0 |

^Source:

† Using simple assumptions about the mathematical relationship of prevalence and incidence to generate an average duration, then a total rate of ‘exit’ (i.e. remission rate + case-fatality rate (CFR) + background mortality rates), and then estimating the case-fatality rate given the five year relative survival ≈[Remission]/[Remission + CFR].

*http://www.health.govt.nz/publication/mortality-and-demographic-data-2010

The DISMOD output rates (in one year age groups) for incidence, prevalence, case-fatality and remission were then used to populate the multistate life table model for all diseases – except CHD, stroke, COPD and lower respiratory tract infection (LRTI). For CHD, stroke and COPD, only incidence, prevalence, and case-fatality were used (i.e. remission was assumed to be zero as these are usually life-long conditions). For LRTI, only mortality was used.

Disability rates (DRs) were calculated by dividing the NZBDS’s disease-specific pYLDs (adjusted for other co-morbidities, for the year 2006, projected to 2011) by the DISMOD II estimated prevalent cases for all diseases except LRTI. To estimate the pYLDs in 2011 we applied the following equation:


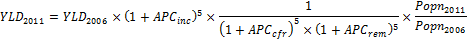


(1)

Where:

YLD[2006] is the corrected for comorbidities YLD in 2006 from NZBDS

APC[inc] = annual percentage change in incidence rate for each disease

APC[cfr] = annual percentage change in CFR for each disease

APC[rem] = annual percentage change in remission rate for cancers only

Popn[2006] = the population count/size for the given sex by age by ethnic group in 2006

Popn[2011] = the population count/size for the given sex by age by ethnic group in 2011

The disease parameters (mortality rates and disability rates) for LRTI were generated directly from the NZBDS (not DISMOD II). For specific details on final parameters for each disease, see Table S3 below.

Generating DRs by dividing pYLDs by prevalent cases for each 5-year age group, for each disease, for each sex by ethnicity, was often too unstable due to sparse data. We therefore aggregated age groupings to ensure the sum of prevalent cases exceeded 10 (e.g. 0-44 year olds were always combined; for common diseases such as CHD and stroke age groupings were: 0-44, 45-54, 55-64, 65-74, and 85+ years; for rare diseases such as pancreatic cancer in Māori males all age groups were combined). For LRTI only, we calculated DRs using the 2006 NZBDS corrected pYLDs divided by the NZBDS population (Statistics NZ) (i.e. no adjustment to 2011 was made for LRTI).

**Table S3: Final disease parameters and sources used in the BODE^3^ tobacco multistate life table model**

| **Disease** | **Incidence** | **Prevalence** | **Case-fatality rate** | **Mortality rate** | **Remission** | **Disability rate** |  |
| --- | --- | --- | --- | --- | --- | --- | --- |
| Lung cancer | DISMOD II | DISMOD II | DISMOD II |  | DISMOD II | DISMOD II & NZBDS | |
| Head & neck cancer | DISMOD II | DISMOD II | DISMOD II |  | DISMOD II | DISMOD II & NZBDS | |
| Esophageal cancer | DISMOD II | DISMOD II | DISMOD II |  | DISMOD II | DISMOD II & NZBDS | |
| Stomach cancer | DISMOD II | DISMOD II | DISMOD II |  | DISMOD II | DISMOD II & NZBDS | |
| Liver cancer | DISMOD II | DISMOD II | DISMOD II |  | DISMOD II | DISMOD II & NZBDS | |
| Pancreatic cancer | DISMOD II | DISMOD II | DISMOD II |  | DISMOD II | DISMOD II & NZBDS | |
| Cervical cancer | DISMOD II | DISMOD II | DISMOD II |  | DISMOD II | DISMOD II & NZBDS | |
| Bladder cancer | DISMOD II | DISMOD II | DISMOD II |  | DISMOD II | DISMOD II & NZBDS | |
| Kidney cancer | DISMOD II | DISMOD II | DISMOD II |  | DISMOD II | DISMOD II & NZBDS | |
| Leukemia | DISMOD II | DISMOD II | DISMOD II |  | DISMOD II | DISMOD II & NZBDS | |
| Endometrial cancer | DISMOD II | DISMOD II | DISMOD II |  | DISMOD II | DISMOD II & NZBDS | |
| Melanoma | DISMOD II | DISMOD II | DISMOD II |  | DISMOD II | DISMOD II & NZBDS | |
| Thyroid cancer | DISMOD II | DISMOD II | DISMOD II |  | DISMOD II | DISMOD II & NZBDS | |
| COPD | DISMOD II | DISMOD II | DISMOD II |  |  | DISMOD II & NZBDS | |
| LRTI+ |  |  |  | NZBDS |  | NZBDS | |
| CHD | DISMOD II | DISMOD II | DISMOD II |  |  | DISMOD II & NZBDS | |
| Stroke | DISMOD II | DISMOD II | DISMOD II |  |  | DISMOD II & NZBDS | |

+ Indicates that disease parameters were not processed in DISMOD II, rather parameters were directly inserted into the model. This acute infection was only used to adjust pYLDs in the model (see “Model design, purpose and specification” section above)

### Final processing of incidence and prevalence estimates

**In an effort to more accurately reflect the disease epidemiology in the New Zealand population, some diseases incidence and prevalence rates were forced to be zero at young ages as a final step in processing. Specifically, the incidence/prevalence rates for cancers (except thyroid cancer) were set to 0 for those 20 years and younger. This led to changes for Non-Māori (males [M] and/or females [F]) in the following cancers: head and neck [M, F], stomach [M, F], bladder [M, F], kidney [M, F], lung [F], liver [F], cervical [F], endometrial [F], and melanoma [M]. This led to changes for Māori in the following cancers: stomach [M, F], bladder [M], lung [F], liver [M], cervical [F], endometrial [F], pancreatic [M, F], and melanoma [M, F]. Starting ages for non-zero incidence/prevalence for stroke and CHD were 25 years and 19 years respectively, for all sex and ethnicity groups. The incidence/prevalence of COPD was set at zero for those aged 15 years or less.**

### Future disease trends (incidence, remission and case-fatality)

The above parameterization was for 2011 only. Some key parameters are known to have increasing or decreasing trends in recent decades – and are likely to have such trends in the near-future. Thus, we also specified future disease incidence and case-fatality as percentage annual change from 2011 to 2026. For CHD and stroke, we relied on NZBDS projections for annual changes in incidence and mortality (see Table 8 in a Report). Specifically, we incorporated an annual incidence change of -2% and an annual case-fatality trend of -2% for CHD and stroke.

For cancer trends, we relied on our previous modeling of future cancer incidence. We generated average incidences for cancer types by sex and ethnicity for age groups 45-84 years for the years 2006 to 2026. Then, we calculated an average annual change. However, since the NZBDS did not develop projections for changes in cancer case-fatalities, we calculated the average case-fatality rate and remission rate for those aged 45-84 years in 2011 (DISMOD outputs/multistate life table disease inputs) by sex and ethnicity for each cancer. We then used the coefficient for year since diagnosis from the Excess Mortality Rate models (Table 30 in Costilla et al;<http://www.otago.ac.nz/wellington/otago032865.pdf>) to calculate annual percentage change in case-fatality and remission.

Trends in COPD incidence and case-fatality till 2026 were difficult to estimate due to the evolving nature of the tobacco epidemic, so we noted that COPD mortality (all sexes and ethnic groups combined) is declining at about 2% per annum and assumed that this was split 50:50 between incidence and case-fatality trends, and applied this uniformly to all four sex by ethnic groups (given the recent similar relative reduction in tobacco use by these groups). We assumed no annual trends in incidence or case-fatality for LRTI.

Uncertainty around the incidence, case-fatality and remission disease trends were included in the model for all diseases of 1 percentage point SD about the APC. This uncertainty draw is independent for each epidemiological parameter (i.e. incidence, case-fatality and remission) by disease, but correlated r=1.0across each of the four sex by ethnic groupings and all diseases.

### Disease cost inputs

Cost offsets, due to reduced rates of tobacco-related disease, are calculated using the five types of health system cost described above in this Appendix, and changing the ‘flow’ through the multistate life table by altering disease incidence. We sourced these five costs from the New Zealand HealthTracker database. This database is a linkage system for all nationally collected health events, linked together by a personal unique identifier. Critically, each health event is linked to a unit resource cost, creating a powerful routine costing system to use in disease costing. The health events include pharmaceuticals, laboratories, laboratory usage, inpatient hospitalizations (albeit with some gaps, most notably rest-home care) and outpatient attendance. General practice is included, but only as an ‘average’ capitated funding formula attributed to individuals. All costs are in 2011 New Zealand dollars. At the time of writing this Appendix (late 2014), HealthTracker costs were available for the 2006 to 2010 period. Over time, we plan to update these costs (on the BODE^3^ website).

We calculated costs such as ‘the health system costs for males age 57 in their first year post diagnosis with a stroke’, and more generally the five costs described above, namely:

1. Annual cost to the New Zealand health system for being alive for a given sex and age, and *not* in the last six months of life and *not* concurrently alive with one of the modeled diseases (i.e. tobacco-related in this Report). All members of the cohort are assigned this cost; it is the base cost.
2. Excess cost to A for being in the last six months of life if dying of a disease *other than* one of the modeled diseases (i.e. dying of a non-tobacco-related disease).
3. Excess cost to A for being in first year of diagnosis of a tobacco-related disease.
4. Excess cost to A for being alive with a given disease, and neither in the first year of diagnosis nor in the last six months of life if dying of that tobacco-related disease.
5. Excess cost to A for being in the last six months of life if dying of the tobacco-related disease.

The A and B costs are relatively simply calculated directly from HealthTracker data. Importantly, the excess disease costs (C and D costs) we calculate are not for mutually exclusive groups of people. That is, when calculating the excess costs to A for people in their first year of diagnosis with CHD, some of these people may also have new/prevalent other disease such as stroke and cancer. If we do not adjust for this, the model will overestimate the total health system cost. (This is very similar to the need to identify separately and independently the comorbidity of each disease.) To adjust for this we undertook the following:

- For each age group we calculated total proportion of C and D together (including all diseases).
- Then we calculated proportion by disease for C and D streams.
- The ratio of the former to the latter is then used to scale down C and D costs.

(Workings and spreadsheets are available from the authors on request.)

As a high-level calibration/logic check, we calculated the following.


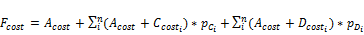
 (2)

and


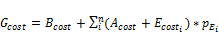
 (3)

Where
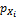
 is probability of having disease
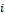
 in C, D or E phases, and the *F* and *G* costs are the total costs for all citizens in New Zealand not in the last six months of life and in the last six months of life, respectively. That is, we confirmed that the disaggregated costs by disease did actually sum to the observed ‘envelope’.

People who do not have any of the diseases of interest in the model will get costs A and B if they are alive or within the last year of life (which assuming they all die half way through this annual cycle equates to using costs in last six months of life) and dying of a non-tobacco-related cause.

People with disease will get
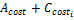
in the first year of diagnosis with disease
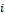
 ,
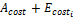
 when in the last six months of life and dying from disease
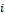
, and
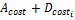
otherwise if a prevalent case of disease
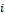
.

Whilst HealthTracker is a rich resource, it is also not (yet) complete. About two thirds of all of New Zealand Government expenditure on health can be accounted for in the above costings assigned to each health event. Remaining Government expenditure on health was assumed to be ‘overhead’ and not able to be altered through future changes in disease incidence. However, there were two remaining ‘missing’ health system costs. First, publicly funded palliative care was not (yet) well captured in this database. Therefore, we scaled up costs in the last six months of life (B and E costs) by 1.1, 1.2 and 1.3 for 65-74, 75-84 and 85+ year olds respectively. Second, 83.2% of all health system expenditure is publicly funded in New Zealand (which is fairly typical of high-income countries); and we assumed that the remaining private and out-of-pocket expenditure was evenly distributed across diseases and simply scaled up all costs by 1.2 (i.e. 1/0.832).

For lower respiratory tract infections (LRTI) only, we generated total costs by age and sex from HealthTracker, which included only hospitalizations. We averaged these annual costs from 2006/7 to 2011/12. Then, we used 2001Australian total health system costs (which included in and out patient hospitalizations, pharmaceuticals, GP visits, imaging and pathology) to generate the scalar to increase our HealthTracker costs to include all other health system costs associated with LRTI, but yet not available using HealthTracker data. We generated a total LRTI cost per age and sex group and divided by the LRTI pYLDs (from NZBDS 2006) for those same age and sex groups.

For examples of cost parameters by disease, see Appendix D. All costs are being made available on the BODE^3^website ([www.otago.ac.nz/bode3](http://www.otago.ac.nz/bode3)), and are subject to ongoing improvements.

### Validation

We conducted a number of tests on the base model, to ensure that disease estimates corresponded with external data sources, with a focus on the main disease drivers in the model (i.e. lung cancer, CHD and stroke). Specifically, we verified our model’s estimated mortality rates for 2011 for four different non-Māori age groups by sex for CHD, stroke and lung cancer compared to those of the Ministry of Health Mortality and Demographic data for 2010 (<http://www.health.govt.nz/publication/mortality-and-demographic-data-2010>).and Burden of Disease Study (updated from 2006 to 2011 estimates using the same methods outlined in Appendix A). We also verified our model’s estimate of the proportion of deaths in New Zealand due to CHD and stroke in 2011 for six to eight different age groups by sex with those of the Ministry of Health’s Mortality and Demographic data for 2010.

For CHD and stroke mortality rates, trends were very similar between our model estimates and the Ministry of Health data for both males and females and across ages. For lung cancer mortality rates, our model estimates for males were slightly lower for males and this difference was less pronounced at older ages. For stroke deaths as a percentage of total deaths, model estimates for both females and males were within 3% of Ministry of Health estimates. Last, for CHD deaths as a percentage of total deaths, model estimates were within 4% or 3% of Ministry of Health estimates for females and males, respectively, where model estimates were slightly higher for males (see Fig.S8 to S12 in Appendix B).

## Model: Intervention specification and parameters

### Health impact of changing exposure to tobacco smoke

We modeled the health benefits of interventions through a reduction in incidence of each smoking-related disease (Equation 4). Note that all calculations were done by age, gender and ethnicity, although we omit these subscripts from the following equations for clarity.

$\text{I}_{x}' = \text{I}_{x} \times\left(1 - \text{PIF}_{x}\right)$ (4)

where:

$\text{I}_{x}$ is the current incidence of disease *x* in the population;

$\text{I}_{x}'$ is the new incidence of disease *x* after an intervention is implemented; and

$\text{PIF}_{x}$ is the population impact fraction for disease *x*.

Each PIF was derived from the current smoking prevalence, the new smoking prevalence following intervention and the relative risks of smoking-related diseases (Equation 5).

(5)

where:

$\text{RR}_{x c}$ is the risk of disease *x* in current smokers, relative to never smokers;

$\text{RR}_{x f}\left(t\right)$ is the risk of disease *x* among former smokers at time *t* since cessation, relative to never smokers;

$\text{p}_{c}$ and $\text{p}'_{c}$ are the prevalence of people who report currently smoking at least one cigarette daily, before and after intervention;

$\text{p}_{n}$ and $\text{p}'_{n}$ are the prevalence of prevalence of people who report never having smoked at least one cigarette daily, before and after intervention; and

$\text{p}_{f}$ and $\text{p}'_{f}$ are the prevalence of prevalence of people who report past smoking of at least one cigarette daily, before and after intervention.

### Relative risks of smoking-related diseases

We applied risks of smoking-related disease from New Zealand-specific epidemiological studies where possible. Unlike the large cohort studies, such as the US based Cancer Prevention Study II (CPS II), the New Zealand studies make use of national cancer registry (and mortality) data linked with data from the regular censuses, which have episodically included questions on smoking behaviour since 1976. For risks of chronic obstructive pulmonary disease and lower respiratory tract infections, however, we had to draw on international studies.Table S4 gives a summary of all the relative risk values used in the modeling and their sources.

Table S4: Relative risks of smoking-related diseases for current versus never smokers

| **Disease** | **Relative risk (95% uncertainty interval)** | **Confounding adjustment*** | **Source** |
| --- | --- | --- | --- |
| Coronary heart disease | Men: 1.61 (1.44 to 1.80)  Women: 1.66 (1.27 to 2.17) |  | New Zealand linked data |
| Stroke | Men: 2.52 (2.12 to 2.99)  Women: 2.20 (1.66 to 2.90) |  | New Zealand linked data |
| Chronic obstructive pulmonary disease | Men: 10.80 (8.40 to 13.90)  Women: 12.30 (9.90 to 15.20) |  | CPS-II |
| Lower respiratory tract infection | Men: 1.90 (1.50 to 2.40)  Women: 2.20 (1.70 to 2.80) |  | CPS-II |
| Lung cancer | 9.28 (8.31 to 10.40)** |  | New Zealand linked data |
| Mouth and oropharyngeal cancer | 2.30 (1.94 to 2.72) ** |  | New Zealand linked data |
| Esophageal cancer | 2.14 (1.73 to 2.65) ** | -0.4 to -0.1 | New Zealand linked data |
| Pancreatic cancer | 1.68 (1.44 to 1.96) ** | 0.1 to 0.2 | New Zealand linked data |
| Bladder cancer | 2.22 (1.94 to 2.55) ** |  | New Zealand linked data |
| Kidney cancer | 1.29 (1.07 to 1.56) ** |  | New Zealand linked data |
| Stomach cancer | 1.42 (1.22 to 1.66) ** | 0.07 to 0.10 | New Zealand linked data |
| Liver cancer | 1.75 (1.37 to 2.24) ** | -0.10 to -0.20 | New Zealand linked data |
| Cervical cancer | 1.82 (1.51 to 2.20) ** |  | New Zealand linked data |
| Endometrial cancer# | 0.67 (0.56 to 0.79) ** | 0.08 | New Zealand linked data |
| Melanoma# | 0.62 (0.56 to 0.69) ** |  | New Zealand linked data |
| Thyroid cancer# | 0.76 (0.58 to 1.00) ** |  | New Zealand linked data |
| * Additional change in RR to additionally correct for potential residual confounding by alcohol and obesity (which were not captured in census data).  **Rate ratio values adjusted for selection bias and confounding (e.g. due to socio-economic factors) and exposure misclassification.  # That is, there is some evidence that tobacco smoking protects against these cancers (possibly due to hormonal effects) | | | |

(Note that we did include a scenario analysis substituting the CPS II relative risks for CHD and stroke for the ‘default’ relative risks above, as presented in Table S7.)

We assumed no excess risk of lower respiratory tract infection immediately after cessation of smoking, but for all other diseases, we assumed that the current smoker disease risks decline with time since cessation, using regression models derived by Hoogenveen et al. The regression model parameters are shown in Table S5. We assumed no excess risk remained after 20 years.

$\text{RR}_{x f}\left(t\right) = 1 + \left(\text{RR}_{x c} - 1\right) \exp\left[-\gamma t\right]$ (6)

$\gamma= \gamma_{0} \exp\left[-\eta\left(a - 50\right)\right]$ (7)

where:

$\gamma$ is a regression coefficient for time (*t*);

$\gamma_{0}$ is the regression coefficient value at age 50 (minimum observed age of age gradient ); and

$\eta$ is a regression coefficient for age (*a*);

Table S5: Regression coefficients used to estimate declining risks of disease with smoking cessation

| **Disease** | ***γ*_0_** | ***η*** |
| --- | --- | --- |
| Coronary heart disease | 0.24228 | 0.05822 |
| Stroke | 0.31947 | 0.01648 |
| Chronic obstructive pulmonary disease | 0.20333 | 0.03087 |
| ***Cancers*** |  |  |
| Lung cancer | 0.15637 | 0.02065 |
| Mouth and oropharyngeal cancer | 0.0493028 | 0 |
| Esophageal cancer | 0.0537424 | 0 |
| Pancreatic cancer | 0.09279 | 0 |
| Bladder cancer | 0.05417 | 0 |
| Kidney cancer | 0.0385957 | 0 |
| Stomach cancer | 0.0264112 | 0 |
| Liver cancer* | 0.0525 | 0 |
| Cervical cancer* | 0.0525 | 0 |
| Endometrial cancer* # | 0.0525 | 0 |
| Melanoma* # | 0.0525 | 0 |
| Thyroid cancer* # | 0.0525 | 0 |
| * Estimated as average of all other cancers, excluding lung cancer.  # For these diseases it is the decline in time of the protective effect – back to zero. | | |

### Smoking prevalence

Smoking prevalence was projected forward from 2011 (the baseline year for BODE^3^ analyses) under ‘no intervention’ and intervention scenarios, using a “NZ tobacco forecasting model”. This is a simple four-state Markov model (current smokers, former smokers, never smokers and dead) that we have described previously and with a post-2013 census update. The model uses observed data (population, mortality, smoker mortality risks and smoking prevalence) to determine background rates of cessation and uptake; these rates are then used in the model to project smoking prevalence forward in time under changing scenarios of uptake and cessation.

Since we wanted to explicitly examine the effects of a taxation intervention, which was implemented in New Zealand in 2010 (i.e. prior to our baseline year of 2011), we needed to first derive background rates of smoking uptake and cessation that excluded the tax effect. We did this in three steps. First, we re-calculated the price for a pack of 20 cigarettes in 2013 as if no tax had been implemented, taking annual changes in goods and services tax and inflation into account.

Second, using price elasticity values for smoking prevalence, we adjusted the smoking prevalence reported in the 2013 census upwards to reflect the likely prevalence in 2013 if no tax had been implemented. The overall price elasticity of demand for tobacco in New Zealand was -0.47 for manufactured cigarettes over the 2002-2011 period.Applying an elasticity age gradient used by Levy et al in their recent analyses of tobacco taxes in the UK and Finland, and solving by the method of least squares, we derived age group-specific prevalence elasticity values of -0.38 (15-20 years), -0.29 (21-24 years), -0.19 (25-34 years) and -0.10 (35+ years), assuming that the prevalence elasticity is around half of the overall demand elasticity, as per a review by IARC.

Third, using smoking prevalence from the 2006 census and the adjusted 2013 census smoking prevalence, we then re-ran the Markov model to determine background rates of uptake and cessation without the tax effects.

These rates of uptake and cessation were then used as the basis for projecting smoking prevalence forward in time under intervention and no intervention scenarios to calculate the disease PIFs in Equation 5. The initial distribution of the 2011 former smokers according to time since cessation ($\text{p} \left(t\right)_{f}$in Equation 5) was also estimated by running the Markov model, Census data from 2006 to 2011. To estimate future tobacco prevalence uncertainty in the BAU model, we have assumed a larger uncertainty of 0.01 applied uniformly across all these parameters, rather than use the estimated SD for the initiation and cessation parameters (usually about 0.001).

## Model: Analysis

For each intervention, the model was run 4000 times using Monte Carlo simulation. Probabilistic uncertainty that was included for intervention effect sizes (e.g. tax increase, price elasticities, relative risks association smoking with disease incidence), intervention costs (e.g. cost of a new tobacco tax law) and selected baseline parameters (i.e. health system costs were assumed gamma distributed with a standard deviation of +/- 10%).We model higher price elasticities among Māori in the base or main model, with uncertainty. Namely, a 20% higher price elasticity for Māori, with SD 10% normal distribution. We report no ethnic differences in price elasticities as a scenario analysis. **Uncertainty around the starting estimates of incidence and case-fatality have been included in the model. 2011 starting estimates have been assigned a log-normal distribution, SD 5%, with random draws in each iteration separately for incidence and case-fatality, by sex and age, but applied uniformly across ages (i.e. independent uncertainty by sex and age, but 100% correlated uncertainty by age within sex by ethnic groups).**

We also include scenario analyses as outlined below:

- 20% per annum tax increase to 2031
- The same price elasticities for Māori and non-Māori
- 10% per annum increase to 2021
- 10% per annum increase to 2041
- Varying CHD and Stroke RR
- Disease trend continuing post 2026
- 3% discount rate
- 6% discount rate per annum
- Reduced uncertainty (2.5%, 5% and 10% SDs instead of 5%, 10% and 20% SDs)
- Increased uncertainty (10%, 20% and 40% SDs instead of 5%, 10% and 20% SDs)
- Doubling confidence interval about all relative risks used in model.
- Morbidity set to zero, 3% discount rate
- Morbidity set to zero, 0% discount rate

All modeling was undertaken in Microsoft Excel, using the add-in tool Ersatz (EpiGear, Version 1.3) for uncertainty analysis.

# Supplementary results: figures and tables to the main manuscript

Fig. S3: Projected all-cause mortality rates (per person), BAU and post 10% per annum tax increase intervention from 2011 to 2031

a: 45-64, 65-84 and 85+ year olds, by ethnicity (sexes combined)


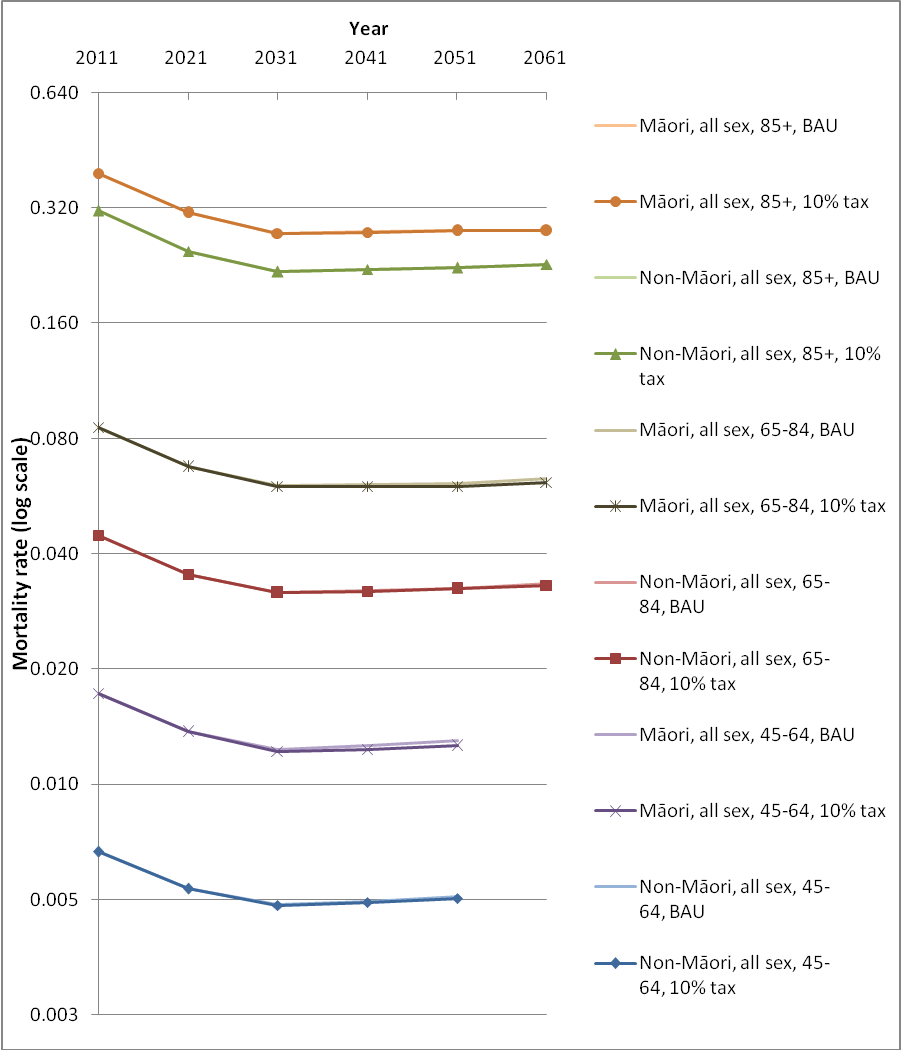


b: 45-64 year olds only, by ethnicity (sexes combined)


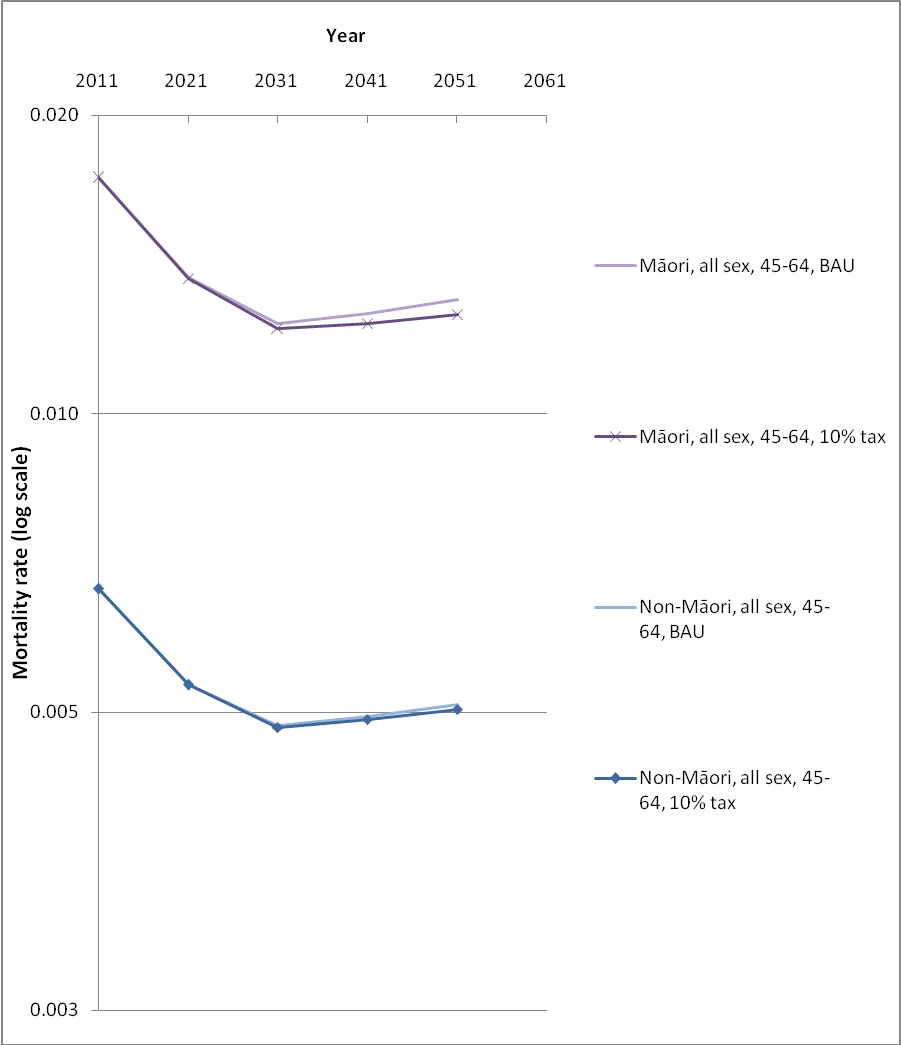


The 2011 values for the all-cause mortality rates are as per Statistics New Zealand, and thereafter are a function of the model parameters (i.e. 2.25%/1.75% per annum decreases for Māori/non-Māori to 2026, then held constant). The key aspect is the ‘gap’ between the BAU and tax intervention scenarios, which are the standardized rate differences presented in Fig. 4 and 5 in the main manuscript – and are not particularly sensitive to variations about the 2.25%/1.75% then 0% structural assumptions for future trends in all-cause mortality rates.

Fig. S4: Projected QALYs gained (1000s) and net health system costs saved (millions) by year for 10% per annum tax increase to 2031, by age cohort in 2011


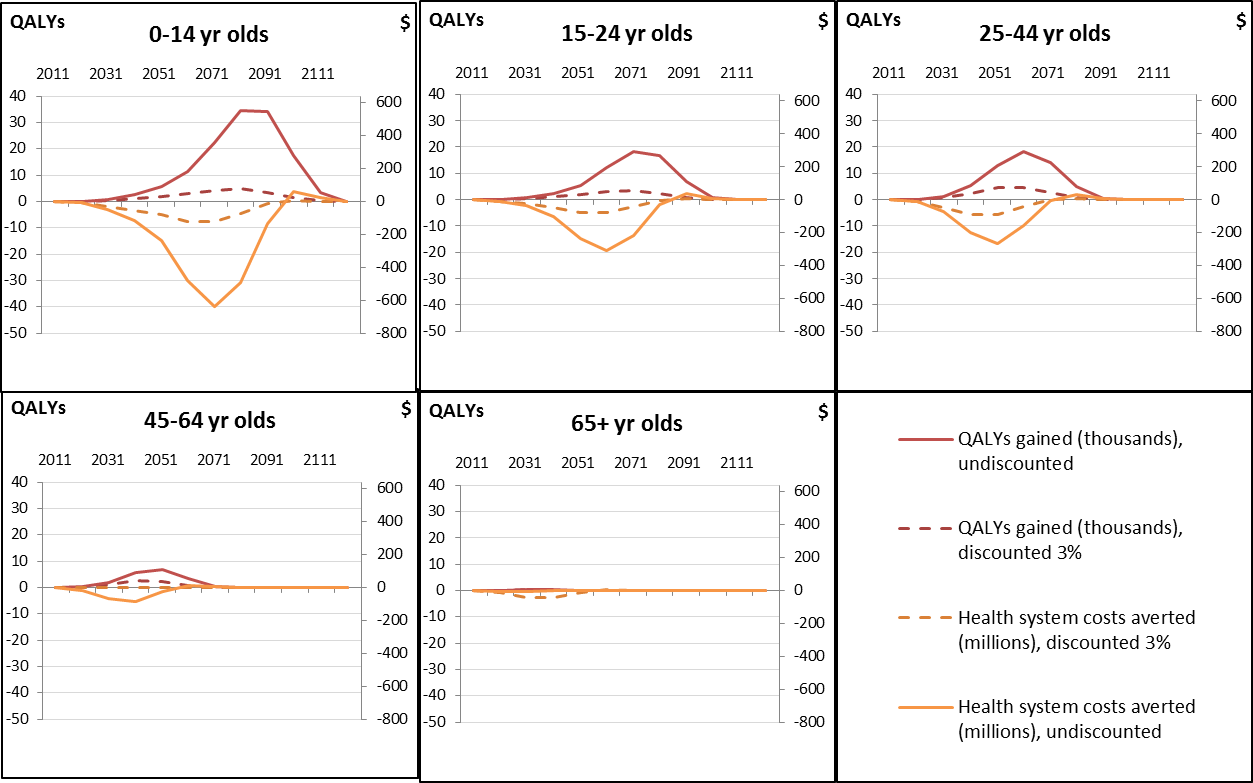


Table S**6: QALYs gained by disease from a 10% per annum increase in tobacco tax (from 2011 to 2031), among the New Zealand population alive in 2011**

|  | **‘Best’ model†** | | | **Alternative model using CPS II relative risks for CHD and stroke‡** | | |
| --- | --- | --- | --- | --- | --- | --- |
|  | **QALYs gained** | **QALY attributable to disease** | **% attributable** | **QALYs gained** | **QALY attributable to disease** | **% attributable** |
| All disease included | **272,000** |  |  | **294,380** |  |  |
| *Cause deleted* |  |  |  |  |  |  |
| CHD | 248,800 | 23,200 | 8.5% | 249,210 | 45,170 | 15.3% |
| Stroke | 252,030 | 19,970 | 7.3% | 273,880 | 20,500 | 7.0% |
| COPD | 129,020 | 142,980 | 52.6% | 151,190 | 143,190 | 48.6% |
| Lung cancer | 198,490 | 73,510 | 27.0% | 220,700 | 73,680 | 25.0% |
| Mouth and oropharyngeal cancer | 269,900 | 2,100 | 0.8% | 292,170 | 2,210 | 0.8% |
| Esophageal cancer | 269,910 | 2,090 | 0.8% | 292,190 | 2,190 | 0.7% |
| Pancreatic cancer | 270,730 | 1,270 | 0.5% | 293,010 | 1,370 | 0.5% |
| Bladder cancer | 268,820 | 3,180 | 1.2% | 291,100 | 3,280 | 1.1% |
| Kidney cancer | 271,520 | 480 | 0.2% | 293,800 | 580 | 0.2% |
| Stomach cancer | 270,580 | 1,420 | 0.5% | 292,860 | 1,520 | 0.5% |
| LRTI | 270,650 | 1,350 | 0.5% | 292,930 | 1,450 | 0.5% |
| Liver cancer | 269,900 | 2,100 | 0.8% | 292,180 | 2,200 | 0.7% |
| Cervical cancer | 271,770 | 230 | 0.1% | 294,050 | 330 | 0.1% |
| Endometrial cancer | 272,480 | -480 | -0.2% | 294,760 | -380 | -0.1% |
| Melanoma | 273,880 | -1,880 | -0.7% | 296,160 | -1,780 | -0.6% |
| Thyroid cancer | 272,170 | -170 | -0.1% | 294,450 | -70 | 0.0% |

†10% per annum tax increase to 2031, undiscounted
‡ Alternate relative risks were used for CHD and stroke, from New Zealand specific estimates in the ‘Best’ model (RR for CHD 1.6 for males and 1.7 for females, and for stroke 2.5 and 2.2) to Cancer Prevention Study (CPS II; RR for CHD 35-64/65+ years 2.6/1.5 for males and 3.2/1.7 for females, and for stroke 2.4/1.5 and 3.8/1.6) in the ‘alternative model’.

Table S7: Scenario analyses about QALY and life year gains and health system cost savings for tobacco tax compared to BAU

| **Scenario**† | **QALYs gained** | **Cost savings (million)** |
| --- | --- | --- |
| ‘Best’ model‡ | 275,000 (158,000 to 431,000; ratio upper to lower 2.73) | $3880 ($2330 to $5970; ratio upper to lower 2.56) |
| *Alternative taxes* |  |  |
| 20% per annum tax increase to 2031 | 499,000 | $7,080 |
| 10% per annum increase to 2021 | 173,410 | $2,510 |
| 10% per annum increase to 2041 | 315,000 | $4,410 |
| *Varying price elasticity by ethnicity* |  |  |
| Price elasticities the same for Māori and non-Māori | 256,000 (111,000 Māori, 145,000 non-Māori) | $3700 (1252 Māori, 1430 non-Māori) |
| *Discount rate* |  |  |
| 3% per annum | 57,500 | $1,160 |
| 6% per annum | 16,000 | $420 |
| *Varying uncertainty* |  |  |
| Reduced uncertainty (2.5%, 5% and 10% SDs instead of 5%, 10% and 20% SDs; see Methods) | 271,000 (207,000 to 347,000) | $3840 ($2940 to $4970) |
| Increased uncertainty (10%, 20% and 40% SDs instead of 5%, 10% and 20% SDs; see Methods) | 288,000 (92,000 to 672,000) | $3970 ($1290 to $8900) |
| Doubling confidence interval about all relative risks used in model. | 276,000 (157,000 to 451,000) | $3870 ($2110 to $6330) |
| *Varying CHD and Stroke RR to CPS II** |  |  |
| All sexes, ages and ethnic groups combined | 294,000 | $4,020 |
| *Disease trend continue post 2026* |  |  |
| All sexes, ages and ethnic groups combined | 369,000 | $3,540 |
| **Life years gained per capita (i.e. no morbidity reduction benefits included in model)** | | |
| *Morbidity set to zero, 3% discount rate* |  |  |
| All sexes, ages and ethnic groups combined | 47,600 (28,000 to 76,600) | na |
| *Morbidity set to zero, 0% discount rate* |  |  |
| All sexes, ages and ethnic groups combined | 259,000 (147,000 to 418,000) | na |
| -          Māori, 0-14 yrs | 74,000 (42,000 to 116,000) | na |
| -          Māori, 15-24 yrs | 31,000 (18,000 to 49,000) | na |
| -          Māori, 25-44 yrs | 24,000 (14,000 to 37,000) | na |
| -          Māori, 45-64 yrs | 5,720 (3,580 to 8,810) | na |
| -          Māori, 65+ yrs | 160 (100 to 250) | na |
| -          Non-Māori, 0-14 yrs | 46,900 (24,400 to 79,500) | na |
| -          Non-Māori, 15-24 yrs | 28,400 (14,600 to 49,100) | na |
| -          Non-Māori, 25-44 yrs | 34,500 (19,800 to 56,300) | na |
| -          Non-Māori, 45-64 yrs | 13,900 (8,200 to 22,200) | na |
| -          Non-Māori, 65+ yrs | 770 (480 to 1,200) | na |

† Alternative taxes and varying price elasticity by ethnicity scenarios are undiscounted
 ‡10% per annum tax increase to 2031, undiscounted, price elasticities 20% higher for Māori
* As in Table S6 (and footnotes).

Table S8: Standardized all-cause mortality rates^†^ (per 100,000) by sex, age and ethnicity projected to 2021 for tax and no tax, and ethnic inequality measures (standardized rate differences (SRD, per 100,000) and ratios (SRR))

|  |  | **2021 under BAU** | | | **2021 with tax** | | | **Percentage decrease post-tax (95% simulation interval)** | | |
| --- | --- | --- | --- | --- | --- | --- | --- | --- | --- | --- |
| **Group** | **Age in 2021** | **Rate** | **SRD** | **SRR** | **Rate** | **SRD** | **SRR** | **Rate** | **SRD (Māori c.f. non-Māori)** | **SRR ^ (Māori c.f. non-Māori)** |
| *Men* |  |  |  |  |  |  |  |  |  |  |
| Māori | 45-64 | 768.5 | 455.6 | 2.455 | 767.0 | 454.3 | 2.453 | -0.2% (-0.29% to -0.13%) | -0.28% (-0.41% to -0.18%) | -0.19% (-0.28% to -0.13%) |
|  | 65-84 | 3691.3 | 1594.5 | 1.760 | 3687.8 | 1591.9 | 1.760 | -0.09% (-0.14% to -0.06%) | -0.16% (-0.24% to -0.1%) | -0.12% (-0.18% to -0.08%) |
|  | 85+ | 16874.1 | 3478.0 | 1.260 | 16871.7 | 3476.5 | 1.260 | -0.01% (-0.03% to 0%) | -0.04% (-0.12% to 0.02%) | -0.04% (-0.12% to 0.03%) |
|  | All 45+ | 1911.1 | 830.8 | 1.769 | 1909.0 | 829.2 | 1.768 | -0.11% (-0.16% to -0.07%) | -0.2% (-0.28% to -0.13%) | -0.15% (-0.22% to -0.1%) |
|  |  |  |  |  |  |  |  |  |  |  |
| Non-Māori | 45-64 | 312.9 |  |  | 312.7 |  |  | -0.09% (-0.13% to -0.06%) |  |  |
|  | 65-84 | 2096.8 |  |  | 2095.9 |  |  | -0.04% (-0.06% to -0.03%) |  |  |
|  | 85+ | 13396.1 |  |  | 13395.2 |  |  | -0.01% (-0.01% to 0%) |  |  |
|  | All 45+ | 1080.2 |  |  | 1079.8 |  |  | -0.04% (-0.06% to -0.03%) |  |  |
| *Women* |  |  |  |  |  |  |  |  |  |  |
| Māori | 45-64 | 604.6 | 384.5 | 2.745 | 602.4 | 382.4 | 2.738 | -0.38% (-0.55% to -0.25%) | -0.53% (-0.78% to -0.35%) | -0.43% (-0.63% to -0.29%) |
|  | 65-84 | 3061.2 | 1623.4 | 2.129 | 3055.4 | 1618.3 | 2.126 | -0.19% (-0.27% to -0.12%) | -0.31% (-0.45% to -0.21%) | -0.26% (-0.38% to -0.17%) |
|  | 85+ | 14208.7 | 2928.2 | 1.260 | 14202.5 | 2923.0 | 1.259 | -0.04% (-0.08% to -0.02%) | -0.18% (-0.34% to -0.06%) | -0.17% (-0.32% to -0.05%) |
|  | All 45+ | 1566.4 | 776.7 | 1.983 | 1563.1 | 773.7 | 1.980 | -0.21% (-0.31% to -0.14%) | -0.38% (-0.55% to -0.25%) | -0.33% (-0.48% to -0.22%) |
|  |  |  |  |  |  |  |  |  |  |  |
| Non-Māori | 45-64 | 220.2 |  |  | 219.9 |  |  | -0.1% (-0.15% to -0.07%) |  |  |
|  | 65-84 | 1437.8 |  |  | 1437.1 |  |  | -0.05% (-0.07% to -0.03%) |  |  |
|  | 85+ | 11280.5 |  |  | 11279.5 |  |  | -0.01% (-0.02% to -0.01%) |  |  |
|  | All 45+ | 789.7 |  |  | 789.3 |  |  | -0.05% (-0.07% to -0.03%) |  |  |
|  |  |  |  |  |  |  |  |  |  |  |
| Sexes combined |  |  |  |  |  |  |  |  |  |  |
| Māori | All 45+ | 1738.7 | 1607.5 | 1.859 | 3472.0 | 1602.9 | 1.857 | -0.16% (-0.23% to -0.1%) | -0.28% (-0.41% to -0.19%) | -0.24% (-0.35% to -0.16%) |
| Non-Māori | All 45+ | 935.0 |  |  | 1869.1 |  |  | -0.04% (-0.07% to -0.03%) |  |  |

† Age-standardized using WHO World Population Mean over 4000 model simulations.

^ Percentage difference in *excess* SRR (i.e. SRR – 1).

Table S9: Standardized all-cause mortality rates^†^ (per 100,000) by sex, age and ethnicity projected to 2031 for tax and no tax, and ethnic inequality measures (standardized rate differences (SRD, per 100,000) and ratios (SRR))

|  |  | **2031 under BAU** | | | **2031 with tax** | | | **Percentage decrease post-tax (95% simulation interval)** | | |
| --- | --- | --- | --- | --- | --- | --- | --- | --- | --- | --- |
| **Group** | **Age in 2031** | **Rate** | **SRD** | **SRR** | **Rate** | **SRD** | **SRR** | **Rate** | **SRD (Māori c.f. non-Māori)** | **SRR ^ (Māori c.f. non-Māori)** |
| *Men* |  |  |  |  |  |  |  |  |  |  |
| Māori | 45-64 | 685.6 | 404.1 | 2.434 | 680.2 | 399.6 | 2.423 | -0.79% (-1.15% to -0.52%) | -1.11% (-1.61% to -0.72%) | -0.77% (-1.11% to -0.51%) |
|  | 65-84 | 3212.8 | 1354.8 | 1.728 | 3198.8 | 1344.3 | 1.724 | -0.43% (-0.66% to -0.27%) | -0.77% (-1.17% to -0.49%) | -0.58% (-0.89% to -0.36%) |
|  | 85+ | 14968.8 | 3203.6 | 1.273 | 14958.4 | 3197.8 | 1.273 | -0.07% (-0.13% to -0.03%) | -0.18% (-0.42% to -0.03%) | -0.14% (-0.37% to 0.01%) |
|  | All 45+ | 1681.2 | 723.2 | 1.755 | 1673.3 | 717.1 | 1.750 | -0.47% (-0.69% to -0.3%) | -0.85% (-1.25% to -0.55%) | -0.67% (-0.98% to -0.43%) |
|  |  |  |  |  |  |  |  |  |  |  |
| Non-Māori | 45-64 | 281.5 |  |  | 280.6 |  |  | -0.34% (-0.5% to -0.22%) |  |  |
|  | 65-84 | 1858.0 |  |  | 1854.5 |  |  | -0.19% (-0.29% to -0.12%) |  |  |
|  | 85+ | 11765.2 |  |  | 11760.5 |  |  | -0.04% (-0.06% to -0.02%) |  |  |
|  | All 45+ | 957.9 |  |  | 956.2 |  |  | -0.18% (-0.27% to -0.11%) |  |  |
| *Women* |  |  |  |  |  |  |  |  |  |  |
| Māori | 45-64 | 546.7 | 344.4 | 2.700 | 538.1 | 336.6 | 2.669 | -1.56% (-2.26% to -1.03%) | -2.24% (-3.23% to -1.49%) | -1.84% (-2.66% to -1.22%) |
|  | 65-84 | 2826.0 | 1518.7 | 2.160 | 2802.7 | 1498.3 | 2.147 | -0.82% (-1.24% to -0.53%) | -1.34% (-2.02% to -0.86%) | -1.12% (-1.69% to -0.72%) |
|  | 85+ | 12509.3 | 2451.0 | 1.243 | 12484.8 | 2430.7 | 1.241 | -0.2% (-0.35% to -0.1%) | -0.83% (-1.49% to -0.42%) | -0.79% (-1.43% to -0.39%) |
|  | All 45+ | 1424.8 | 709.6 | 1.991 | 1411.9 | 698.1 | 1.977 | -0.91% (-1.34% to -0.58%) | -1.61% (-2.38% to -1.05%) | -1.41% (-2.08% to -0.93%) |
|  |  |  |  |  |  |  |  |  |  |  |
| Non-Māori | 45-64 | 202.3 |  |  | 201.5 |  |  | -0.41% (-0.61% to -0.27%) |  |  |
|  | 65-84 | 1307.3 |  |  | 1304.4 |  |  | -0.22% (-0.34% to -0.14%) |  |  |
|  | 85+ | 10058.3 |  |  | 10054.1 |  |  | -0.04% (-0.07% to -0.02%) |  |  |
|  | All 45+ | 715.3 |  |  | 713.8 |  |  | -0.2% (-0.31% to -0.13%) |  |  |
|  |  |  |  |  |  |  |  |  |  |  |
| Sexes combined |  |  |  |  |  |  |  |  |  |  |
| Māori | All 45+ | 1553.0 | 1432.8 | 1.856 | 3085.2 | 1415.2 | 1.847 | -0.67% (-0.98% to -0.43%) | -1.23% (-1.8% to -0.8%) | -1.04% (-1.52% to -0.68%) |
| Non-Māori | All 45+ | 836.6 |  |  | 1670.0 |  |  | -0.19% (-0.29% to -0.12%) |  |  |

† Age-standardized using WHO World Population Mean over 4000 model simulations.

^ Percentage difference in *excess* SRR (i.e. SRR – 1).

Table S10: Standardized all-cause mortality rates^†^ (per 100,000) projected to 2031 by sex, age (in 2031) and ethnicity for tax and no tax to 2031, and sex inequality measures (standardized rate differences (SRD, per 100,000) and ratios (SRR))

|  |  | **2031 under BAU** | | | **2031 with tax** | | | **Decrease post-tax** | | |
| --- | --- | --- | --- | --- | --- | --- | --- | --- | --- | --- |
| **Group** | **Age in 2031** | **Rate** | **SRD** | **SRR** | **Rate** | **SRD** | **SRR** | **Rate** | **SRD** | **SRR ^** |
| *Men* |  |  |  |  |  |  |  |  |  |  |
| Māori | 45-64 | 685.6 | -138.9 | 0.797 | 680.2 | -142.0 | 0.791 | -0.79% (-1.15% to -0.52%) | 2.25% (1.44% to 3.39%) | 3.07% (2% to 4.49%) |
|  | 65-84 | 3212.8 | -386.8 | 0.879 | 3198.8 | -396.1 | 0.876 | -0.43% (-0.66% to -0.27%) | 2.4% (1.53% to 3.61%) | 2.85% (1.81% to 4.3%) |
|  | 85+ | 14968.8 | -2459.5 | 0.835 | 14958.4 | -2473.6 | 0.834 | -0.07% (-0.13% to -0.03%) | 0.57% (0.16% to 1.18%) | 0.64% (0.24% to 1.26%) |
|  | All 45+ | 1681.2 | -256.3 | 0.847 | 1673.3 | -261.4 | 0.843 | -0.47% (-0.69% to -0.3%) | 1.97% (1.25% to 2.97%) | 2.45% (1.57% to 3.68%) |
|  |  |  |  |  |  |  |  |  |  |  |
| Non-Māori | 45-64 | 281.5 | -79.2 | 0.719 | 280.6 | -79.1 | 0.718 | -0.34% (-0.5% to -0.22%) | -0.15% (-0.27% to -0.05%) | 0.19% (0.1% to 0.32%) |
|  | 65-84 | 1858.0 | -550.7 | 0.704 | 1854.5 | -550.1 | 0.703 | -0.19% (-0.29% to -0.12%) | -0.12% (-0.19% to -0.06%) | 0.07% (0.04% to 0.13%) |
|  | 85+ | 11765.2 | -1706.9 | 0.855 | 11760.5 | -1706.4 | 0.855 | -0.04% (-0.06% to -0.02%) | -0.03% (-0.1% to 0.03%) | 0.01% (-0.05% to 0.08%) |
|  | All 45+ | 957.9 | -242.7 | 0.747 | 956.2 | -242.4 | 0.747 | -0.18% (-0.27% to -0.11%) | -0.11% (-0.18% to -0.06%) | 0.07% (0.03% to 0.13%) |
| *Women* |  |  |  |  |  |  |  |  |  |  |
| Māori | 45-64 | 546.7 |  |  | 538.1 |  |  | -1.56% (-2.26% to -1.03%) |  |  |
|  | 65-84 | 2826.0 |  |  | 2802.7 |  |  | -0.82% (-1.24% to -0.53%) |  |  |
|  | 85+ | 12509.3 |  |  | 12484.8 |  |  | -0.2% (-0.35% to -0.1%) |  |  |
|  | All 45+ | 1424.8 |  |  | 1411.9 |  |  | -0.91% (-1.34% to -0.58%) |  |  |
|  |  |  |  |  |  |  |  |  |  |  |
| Non-Māori | 45-64 | 202.3 |  |  | 201.5 |  |  | -0.41% (-0.61% to -0.27%) |  |  |
|  | 65-84 | 1307.3 |  |  | 1304.4 |  |  | -0.22% (-0.34% to -0.14%) |  |  |
|  | 85+ | 10058.3 |  |  | 10054.1 |  |  | -0.04% (-0.07% to -0.02%) |  |  |
|  | All 45+ | 715.3 |  |  | 713.8 |  |  | -0.2% (-0.31% to -0.13%) |  |  |
|  |  |  |  |  |  |  |  |  |  |  |
| Ethnic groups combined |  |  |  |  |  |  |  |  |  |  |
| Men | All 45+ | 1319.6 | -249.5 | 0.811 | 1314.8 | -251.9 | 0.808 | -0.36% (-0.54% to -0.23%) | 0.96% (0.61% to 1.46%) | 1.33% (0.85% to 1.99%) |
| Women | All 45+ | 1070.0 |  |  | 1062.9 |  |  | -0.67% (-1% to -0.43%) |  |  |

† Age-standardized using WHO World Population Mean over 4000 model simulations.

^ Percentage difference in *excess* SRR (i.e. SRR – 1).

Table S11: QALYs gained and health system costs averted, with uncertainty, from a 10% per annum increase in tobacco tax (from 2011 to 2031), among the New Zealand population alive in 2011 (the same as Table 2 in the main manuscript, but showing uncertainty)

|  | **Non-Māori** | | **Māori** | | | **Ethnic groupings combined** | |
| --- | --- | --- | --- | --- | --- | --- | --- |
| **Sex and age (in 2011)** | **QALYs** | **Cost savings (million)** | **QALYs** | **QALYs – equity†** | **Cost savings (million)** | **QALYs** | **Net cost savings (million) ‡** |
| Sex and age groups combined | 145,000 (81,300 to 233,000) | $2430 ($1430 to $3830) | 129,000 (77,300 to 195,000) | 195,000 (117,000 to 305,000) | $1450 ($900 to $2160) | 275,000 (158,000 to 431,000) | $3880 ($2330 to $5970) |
| *Men* |  |  |  |  |  |  |  |
| 0-14 year olds | 29,600 (16,600 to 49,500) | $604 (342 to 969) | 29,100 (17,800 to 45,500) | 44,700 (26,000 to 71,100) | $510 (309 to 776) | 58,700 (34,500 to 94,700) | $1110 (652 to 1740) |
| 15-24 year olds | 18,000 (9,860 to 30,600) | $321 (174 to 527) | 11,900 (7,110 to 18,800) | 18,400 (10,500 to 29,900) | $181 (106 to 282) | 29,900 (17,100 to 49,100) | $502 (281 to 808) |
| 25-44 year olds | 20,000 (11,500 to 32,300) | $285 (157 to 461) | 7,840 (4,800 to 12,200) | 12,500 (7,440 to 19,800) | $93 (54 to 146) | 27,800 (16,400 to 44,300) | $378 (212 to 605) |
| 45-64 year olds | 7,100 (4,160 to 11,400) | $82 (45 to 133) | 1,620 (970 to 2,550) | 2,870 (1,670 to 4,630) | $15 (8.6 to 25) | 8,750 (5,150 to 14,000) | $97 (54 to 158) |
| 65+ year olds | 320 (190 to 510) | $3.0 (1.7 to 4.8) | 30 (20 to 50) | 60 (40 to 110) | $0.4 (0.2 to 0.6) | 350 (210 to 550) | $3.3 (1.9 to 5.4) |
| All ages | 75,100 (42,300 to 124,000) | $1290 (724 to 2090) | 50,500 (30,700 to 79,000) | 78,600 (45,800 to 125,000) | $799 (477 to 1230) | 125,600 (73,600 to 203,000) | $2090 (1200 to 3330) |
| *Women* |  |  |  |  |  |  |  |
| 0-14 year olds | 30,000 (16,500 to 50,300) | $560 (321 to 899) | 44,700 (26,500 to 69,400) | 64,100 (37,900 to 102,000) | $410 (258 to 621) | 74,700 (42,900 to 119,400) | $969 (584 to 1526) |
| 15-24 year olds | 15,700 (8,430 to 27,000) | $273 (152 to 451) | 17,800 (10,500 to 28,100) | 26,100 (15,200 to 41,900) | $143 (88 to 223) | 33,600 (19,000 to 54,900) | $417 (242 to 675) |
| 25-44 year olds | 17,400 (9,980 to 28,400) | $242 (142 to 392) | 13,100 (8,000 to 20,300) | 20,100 (12,100 to 31,400) | $80 (48 to 127) | 30,500 (18,000 to 49,100) | $322 (191 to 517) |
| 45-64 year olds | 6,580 (3,900 to 10,600) | $65 (38 to 105) | 3,050 (1,900 to 4,690) | 5,230 (3,200 to 8,150) | $17 (9 to 27) | 9,630 (5,830 to 15,200) | $82 (47 to 133) |
| 65+ year olds | 370 (230 to 600) | $2.5 (1.5 to 4) | 90 (50 to 150) | 170 (100 to 270) | $0.7 (0.4 to 1.2) | 460 (290 to 720) | $3.2 (1.8 to 5.2) |
| All ages | 70,100 (39,000 to 116,000) | $1140 (660 to 1850) | 78,700 (47,300 to 121,000) | 116,000 (68,700 to 183,000) | $651 (405 to 989) | 149,000 (86,000 to 236,000) | $1790 (1070 to 2840) |
| Per capita (QALYs /1000 people & $) | 39 (22 to 63) | $647 (382 to 1026) | 191 (115 to 290) | 288 (173 to 452) | $2130 (1340 to 3210) | 62 (36 to 98) | $873 (530 to 1356) |

†Māori ‘QALYs equity’ are calculated using non-Māori background mortality and morbidity rates, so as not to ‘penalize’ Māori in terms of future health gain due to poorer current background mortality and morbidity.

‡Includes both the cost offsets and intervention cost, the latter being the cost of a law (NZ$3.5 million, 95% UI $2.0 to $6.2 million ) to introduce tobacco taxes increases of 10% per annum to 2031, distributed pro-rata across all citizens alive in 2011.

# References

# Appendix A: DISMOD II example for lung cancer

| **NZBDS Code** | **NZBDS Description** | **Finalized ICD10 Codes** | **Source of Prevalence (or Incidence) Data** | **Number of health states** |
| --- | --- | --- | --- | --- |
| C09 | Lung | C33-C34 | NZ Cancer Registry and NMDS (MORT) | 4 |

1. **STEP 1: Data compilation and pre-processing of parameters**
   1. **Incidence in 2011**
      1. Using lung cancer incidence regression model outputs for the year 2011 previously generated by BODE^3^, we first weighted estimates by the NZ population within each area-level deprivation tertile for each age (5-year age groups from 25 years to 84 years), sex, and ethnic group (Māori and Non-Māori).
      2. Then, to estimate incidence rates for the older ages, we used a simple linear extrapolation of rates for 85-89, 90-94 and 95-99 by using the trends in cancer registry data for 2008, 2009 and 2010.

<http://www.health.govt.nz/publication/cancer-new-registrations-and-deaths-2010>

<http://www.health.govt.nz/publication/cancer-new-registrations-and-deaths-2009>

<http://www.health.govt.nz/publication/cancer-new-registrations-and-deaths-2008>

- - - 1. Specifically, we calculated the rate ratio (pooled by sex and ethnicity) of the 85+ group over the rate for 80-84 year olds.
      2. Using this rate ratio, we generated rates for 5-year age groups to 99+ years using a linear extrapolation (see example Fig. S5; Excel spreadsheets available on request).

**Fig. S5. Example of linear extrapolation of incidence of lung cancer for older age groups**


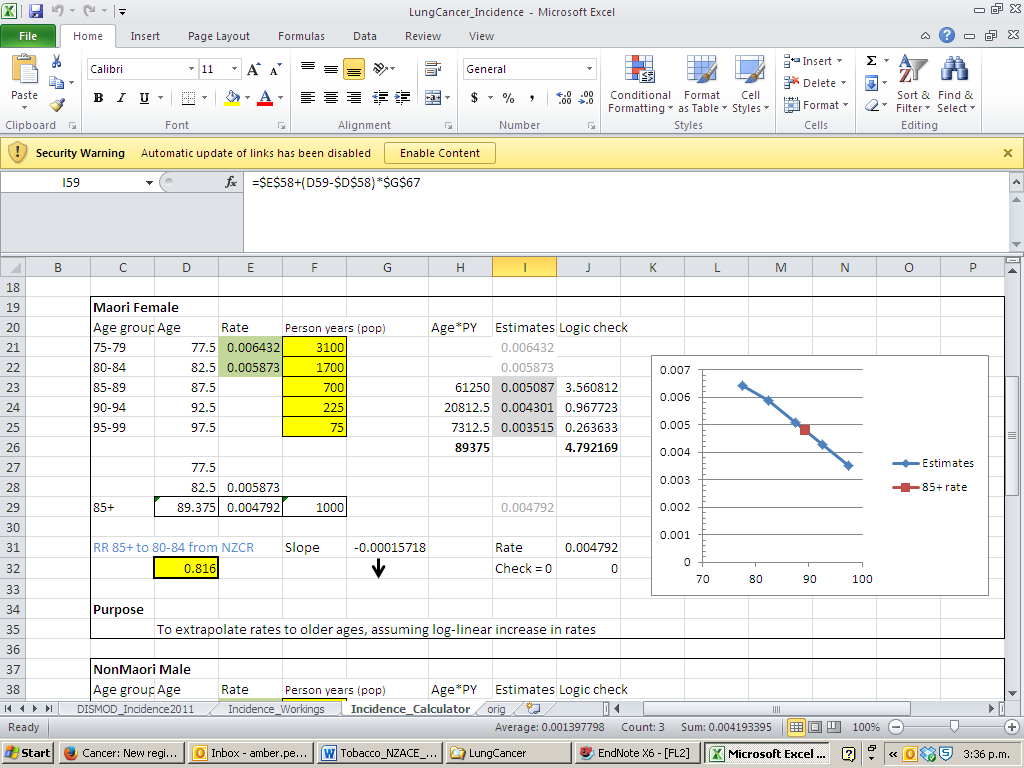


- 1. **Prevalence**
     1. We compiled NZBDS data on prevalence from the year 2006 for 5-year age groups to 85+ years, with a five-year look back period. We opted to keep prevalence rates the same as 2006 rates, as 2011 prevalence is a function of all of changing population counts, changing incidence and changing survival. (NB: It would be possible to estimate 2011 prevalence using a similar equation to that specified in the main Report for pYLDs, but prevalence itself is ‘just’ one input to DISMOD II whereas pYLDs are more directly inputted to the multistate life table model through the disability rates.) Due to this assumption, we down-weighted the importance of this parameter in DISMOD II (see example, Fig. S6).
     2. To calculate a *starting* estimate of 5-year prevalence rates for ages over 84 years, we assumed that [incidence] × [duration].
  2. **Mortality**
     1. We compiled death counts by age, sex and ethnicity from the NZBDS, for the years 2005-2007.
     2. In order to update these counts to the year 2011, we used Cancer Mortality Projections from the Ministry of Health (Table 1).
        1. This document includes 10-year projections, so we manipulated these to get an annual percentage change.
        2. Thus for males, the annual percentage change = -ln(1-0.26) = -2.74%
        3. The formula for the rate in 2011 (5 years of change) = 2006mortrate × exp(annualpercentchange×5 )
     3. Then, to generate estimates for 5-year age groups over 84 years, we used a similar linear extrapolation as seen in Fig. S7.
  3. **Case-fatality rate, Remission rate and Duration (time in years):**
     1. We compiled average background mortality rates (BMR) by age, sex and ethnicity using the Statistics New Zealand life tables for 2010-2012.
     2. We compiled Relative Survival Rates (RSRs) for lung cancer at month 60 (5-years) from internal BODE cancer excess mortality rate report.
     3. Together with our estimates for incidence, mortality and prevalence, these *starting* parameters were generated, using the following formulas:
        1. Crude duration =Prevalence/incidence
        2. Case-fatality rate + Remission = (1/Duration) – BMR
        3. Case-fatality rate = CFR+Remission *(1-RSR)
        4. Remission = CFR+Remission * RSR

*To emphasize, these are just starting parameters to input to DISMOD II, not the parameters used in the multistate life table model.

1. **STEP 2: Processing in DISMOD II software**

Below are examples of the weighting schemes used for lung cancer parameters (Fig. S6) and examples of the smoothed parameters by age, sex and ethnicity (Fig. S7a-d).

**Fig. S6. Example of parameter weighting in DISMOD II**


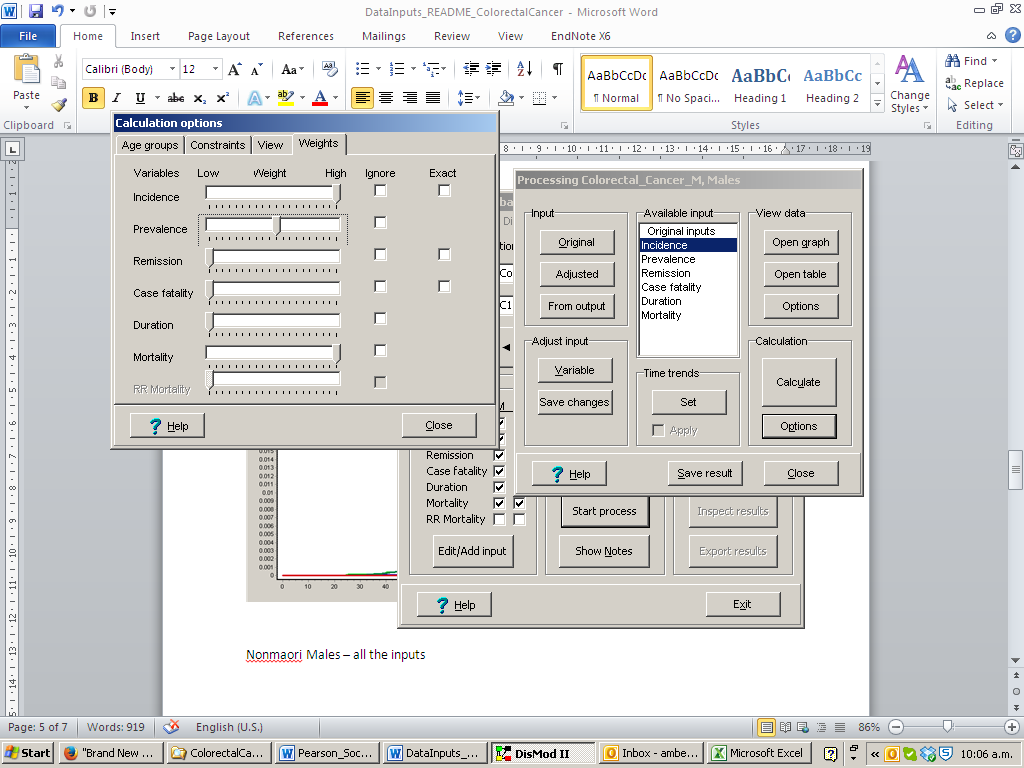


Note that there was sometimes considerable instability in the case-fatality rates at younger ages. This is a function of sparse data, and the case-fatality rate needing to ‘move’ to reconcile with the incidence and mortality inputs (and to a lesser extent prevalence). Once inputted to the BODE^3^ tobacco multistate life table model, it does however balance out to ensure a target mortality rate (which largely drives the health loss/gain).

**Fig. S7 a-d. Example of parameter smoothing in DISMOD II**


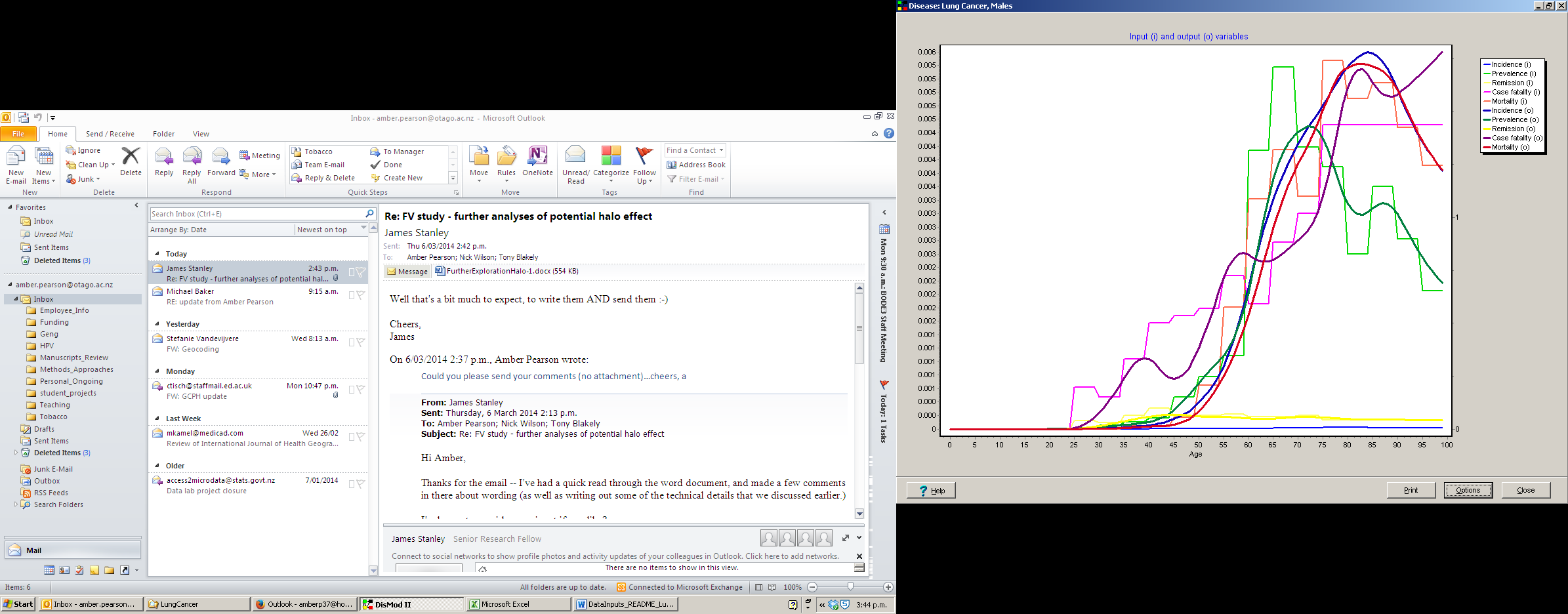

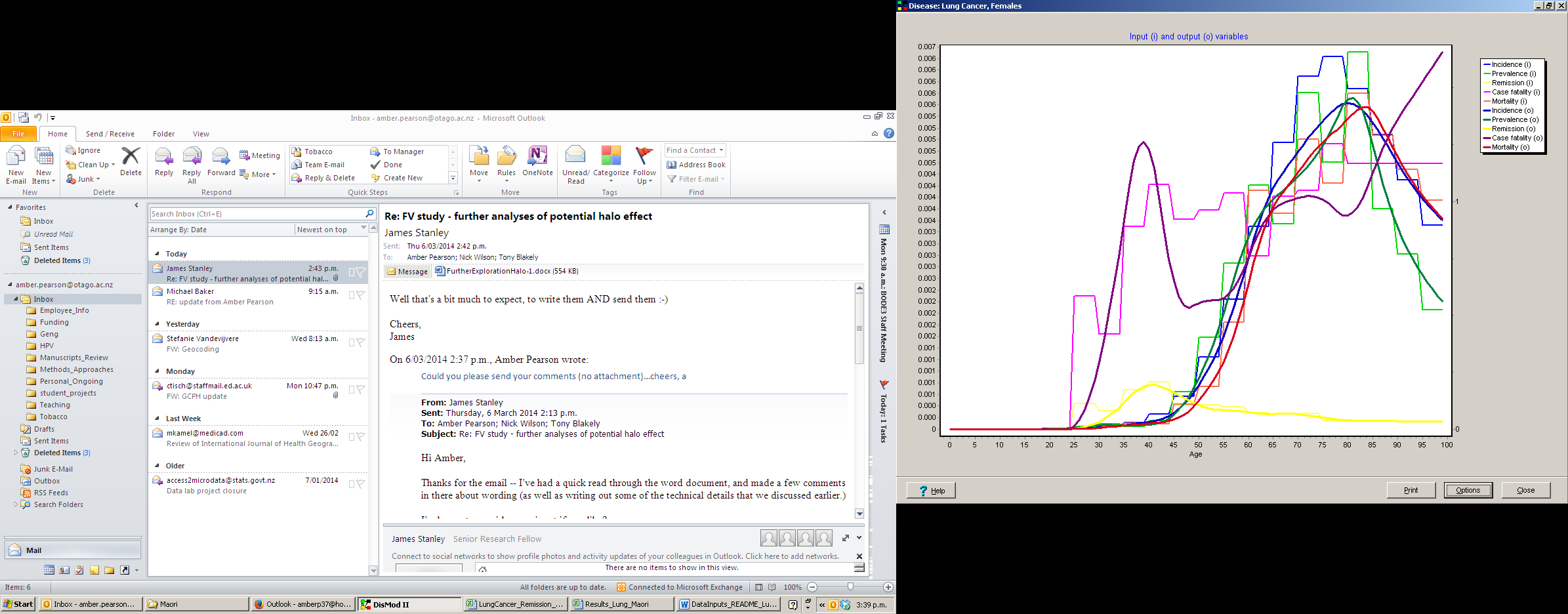

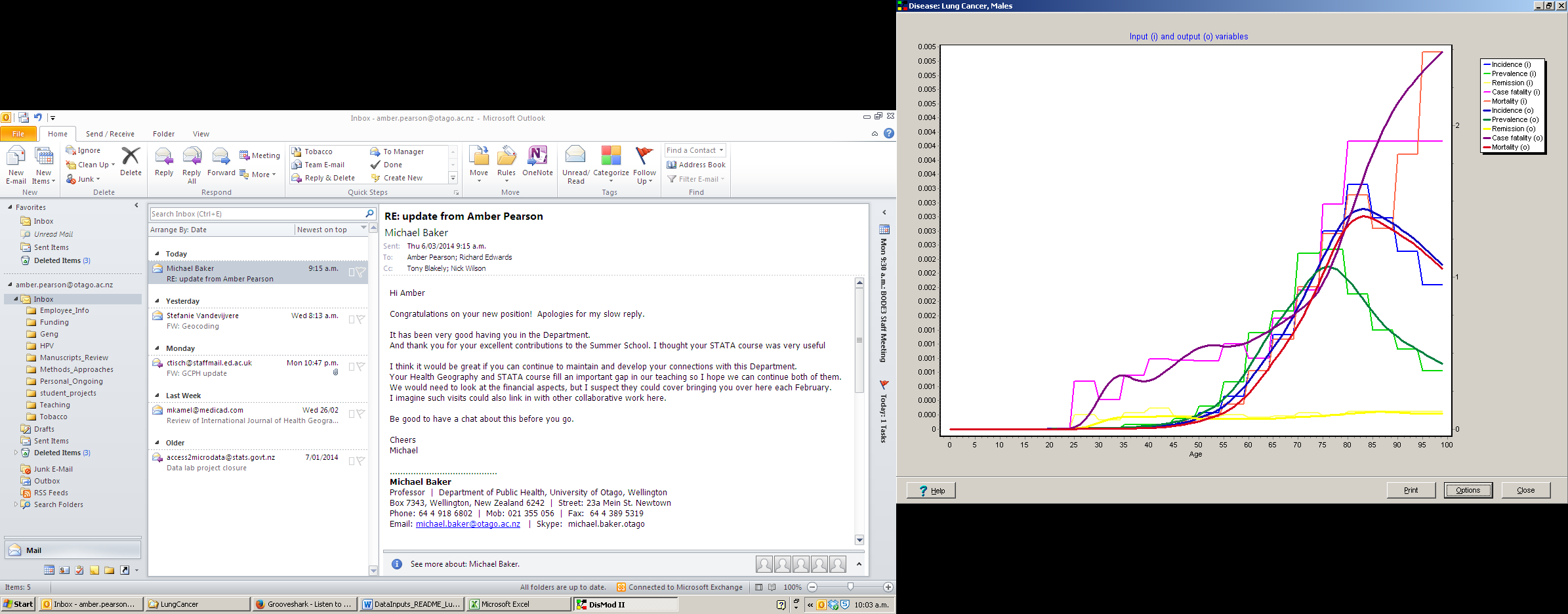

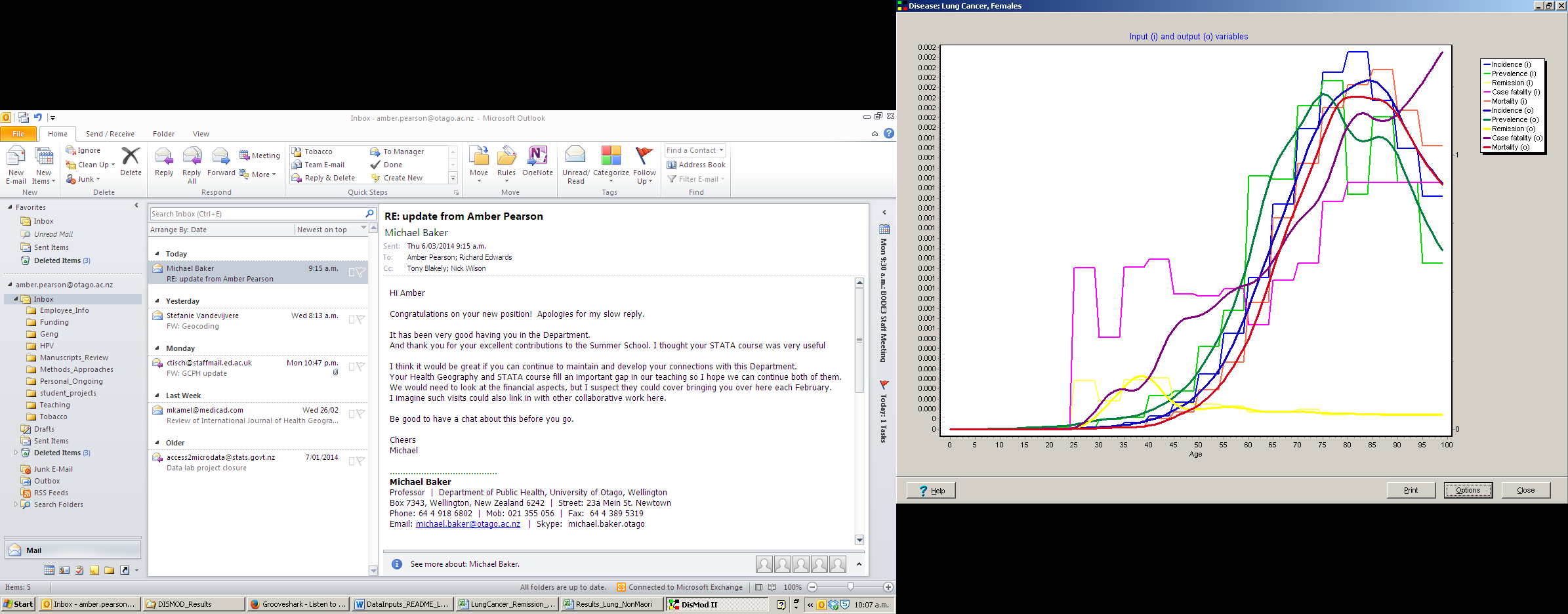

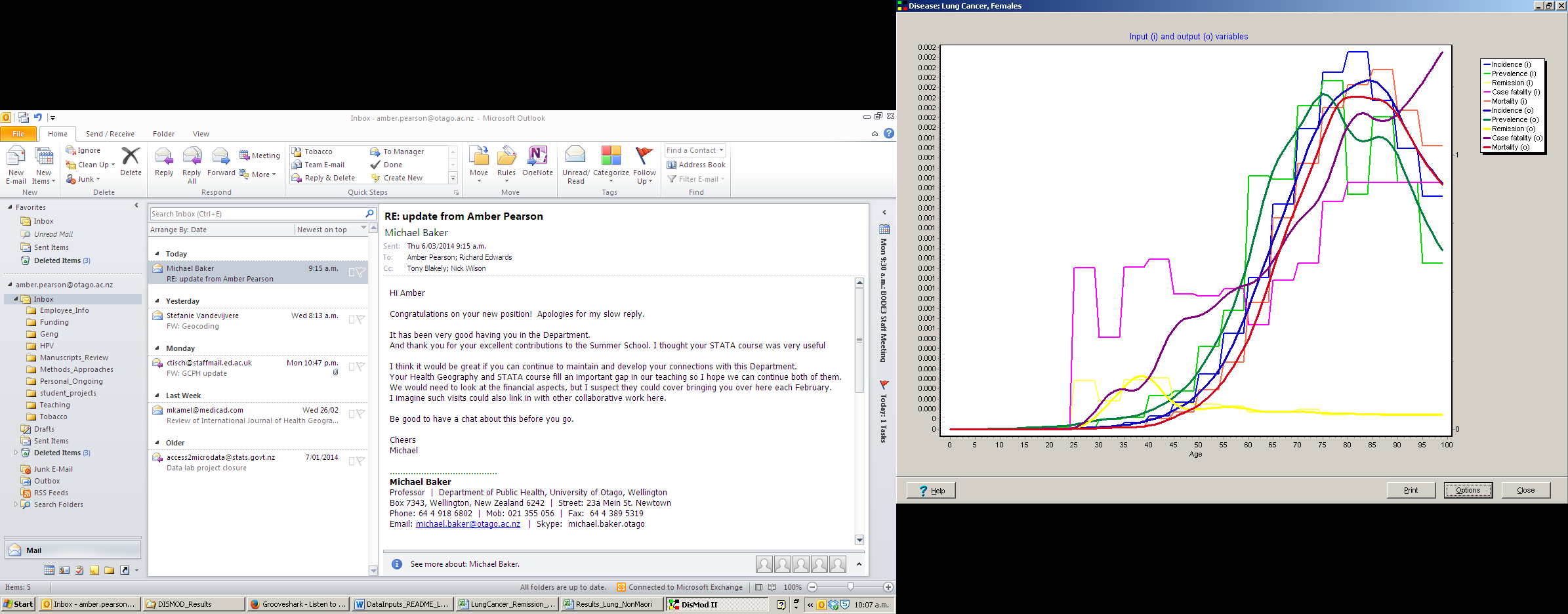


**a Māori males**

**b Māori females**

**c Non-Māori males**

**d Non-Māori females**

*NOTE: Case-Fatality rate scale on right, all others on left

*NOTE: Case-fatality rate on right axis, all others on the left.

# Appendix B: Validation

**Fig. S8. Non-Māori male and female CHD mortality rates in 2011 by age, comparisons between BODE^3^ tobacco multistate life table model and Ministry of health data**


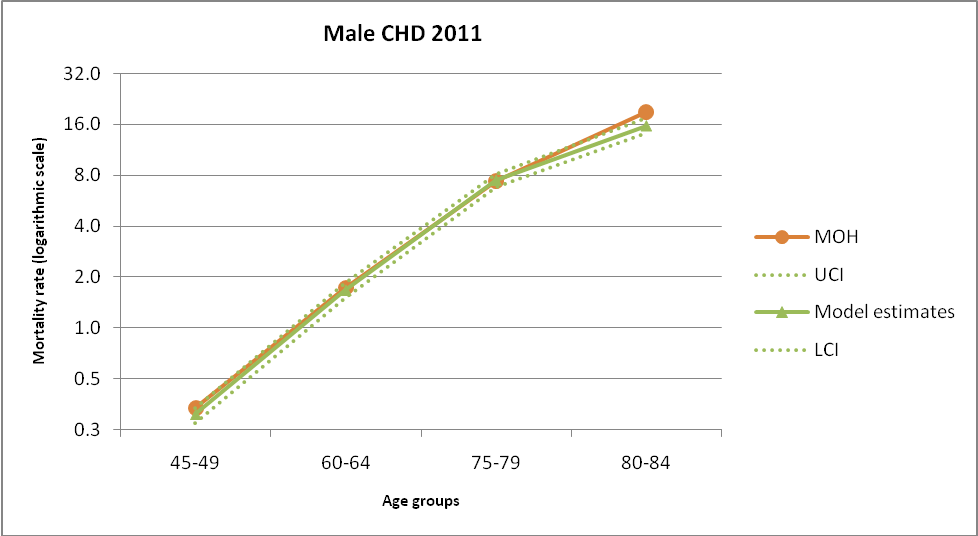


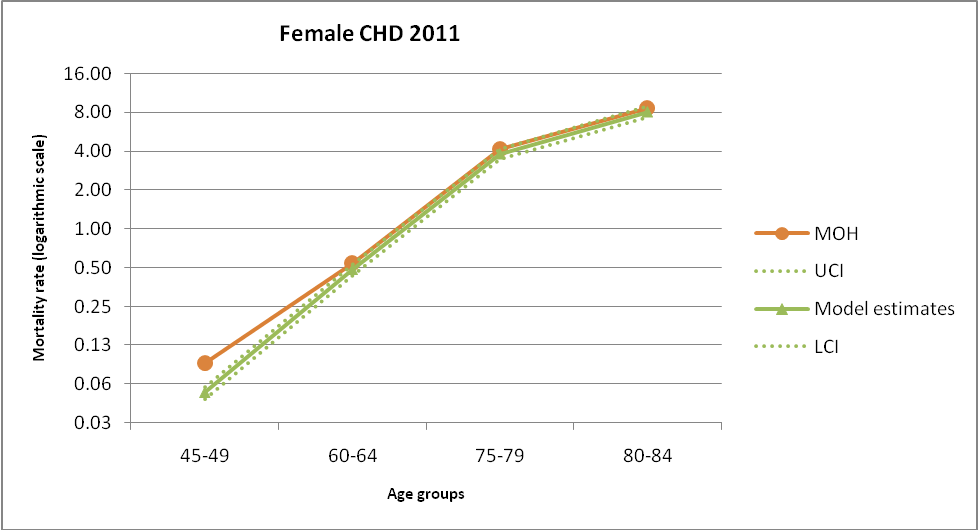


**Fig. S9. Non-Māori male and female stroke mortality rates in 2011 by age, comparisons between BODE^3^ tobacco multistate life table model and Ministry of Health data**


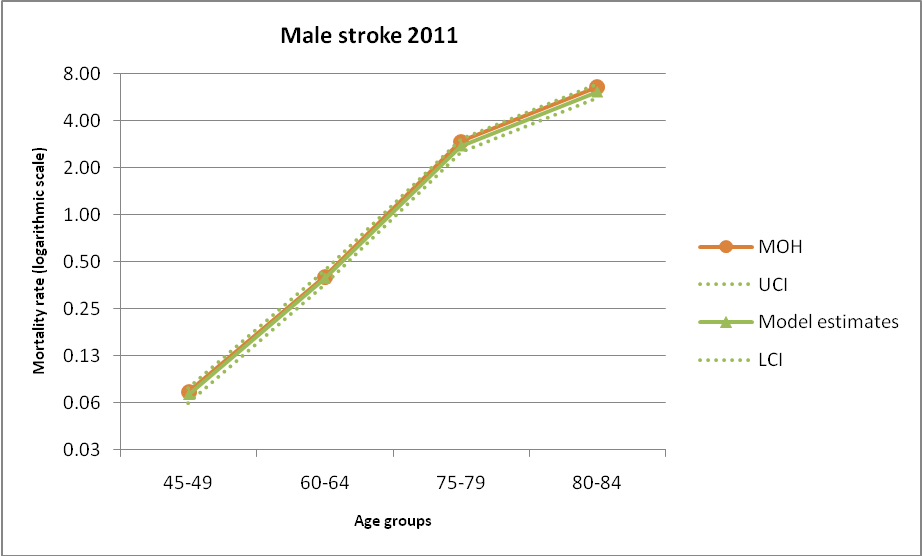


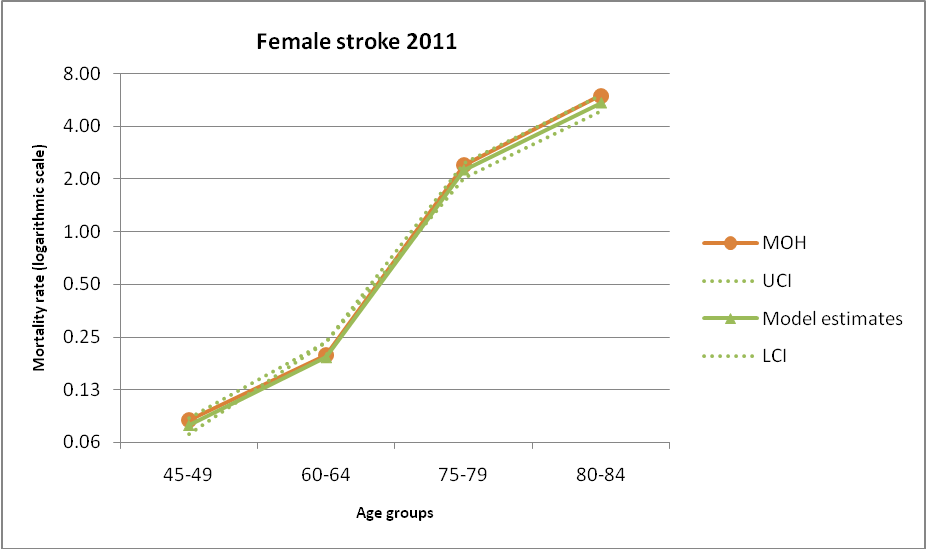


**Fig.S10. Non-Māori male and female lung cancer mortality rates in 2011 by age, comparisons between BODE^3^ tobacco multistate life table model and NZBDS**


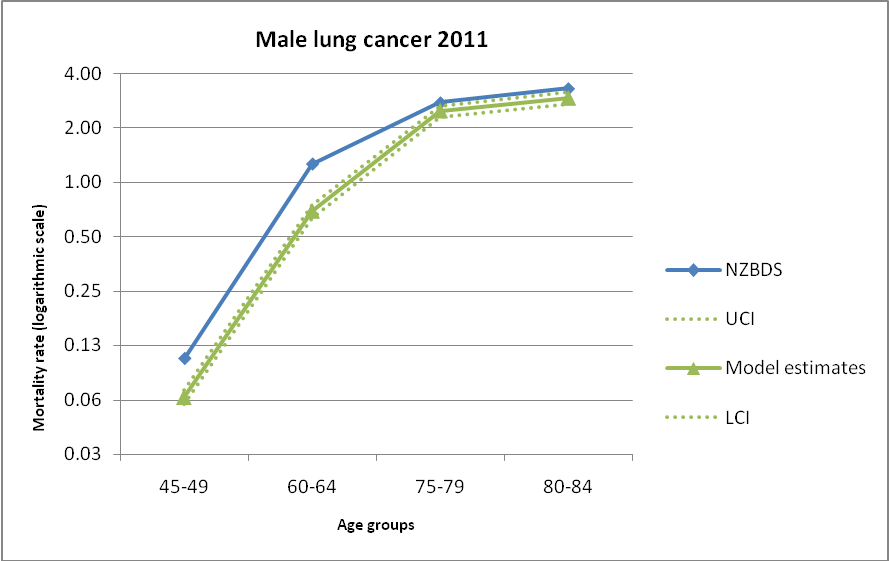


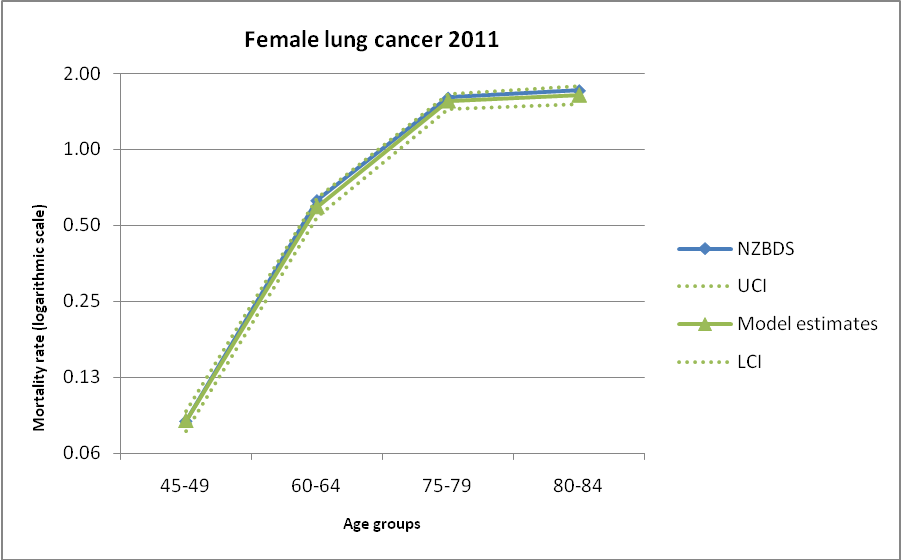


**Fig. S11. Percentage stroke deaths out of all deaths for Non-Māori males and females in 2010/11 by age, comparisons between BODE^3^ tobacco multistate life table model and Ministry of Health estimates**


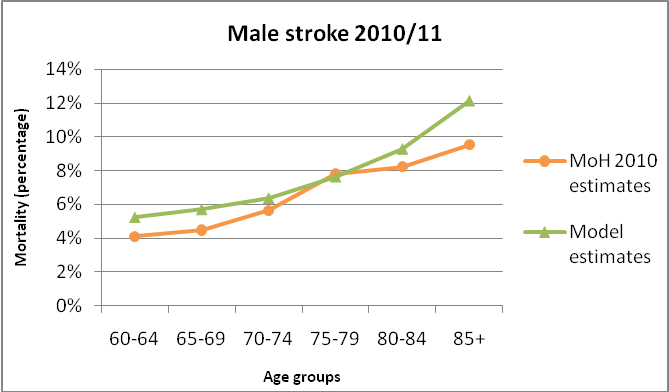

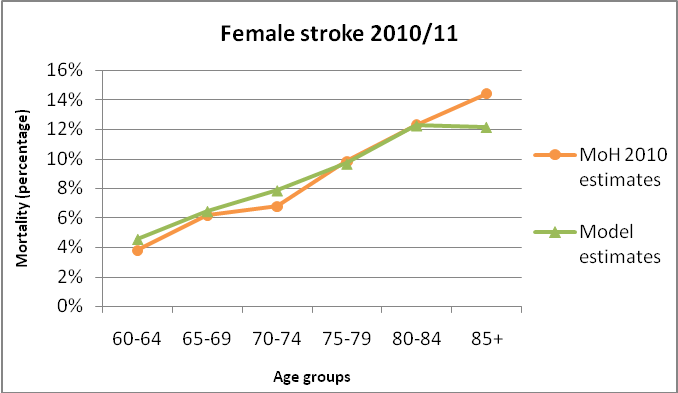


**Fig. S12. Percentage CHD deaths out of all deaths for Non-Māori males and females in 2010/11 by age, comparisons between BODE^3^ tobacco multistate life table model and Ministry of Health estimates**


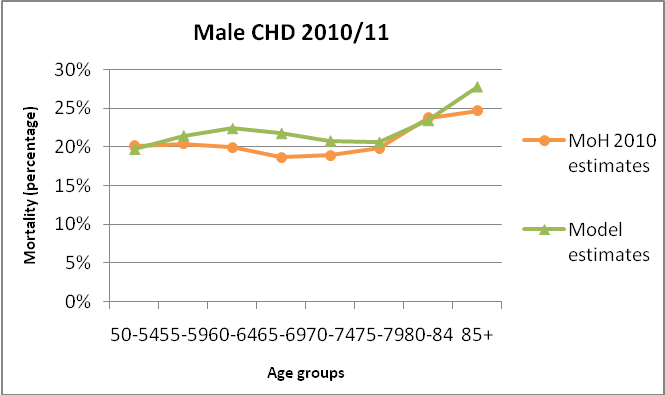

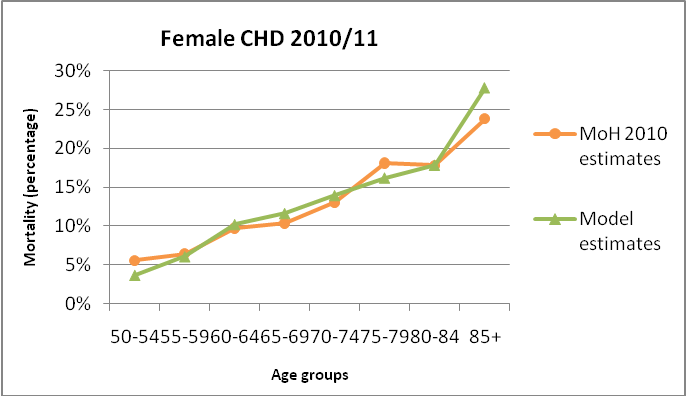


# Appendix C: Epidemiological inputs

This Appendix provides tables of the baseline epidemiological inputs into the BODE3 multistate life table models

Table S12: CHD input parameters

| **Sex** | **Ethnic group** | **Age group (years)** | **Incidence rate per 100,000** | **Case-fatality rate** | **Prevalence per 1000** | **Disability rate** |
| --- | --- | --- | --- | --- | --- | --- |
| Male | Māori | 0-4 | 0.0 | 0.000 | 0.0 | 0.105 |
|  |  | 5-9 | 0.0 | 0.000 | 0.0 | 0.105 |
|  |  | 10-14 | 0.0 | 0.000 | 0.0 | 0.105 |
|  |  | 15-19 | 0.2 | 0.000 | 0.0 | 0.105 |
|  |  | 20-24 | 8.8 | 0.000 | 0.2 | 0.105 |
|  |  | 25-29 | 27.5 | 0.006 | 1.1 | 0.105 |
|  |  | 30-34 | 44.7 | 0.026 | 2.6 | 0.105 |
|  |  | 35-39 | 125.4 | 0.029 | 5.9 | 0.105 |
|  |  | 40-44 | 250.4 | 0.030 | 13.8 | 0.105 |
|  |  | 45-49 | 487.0 | 0.038 | 27.6 | 0.108 |
|  |  | 50-54 | 846.3 | 0.040 | 52.0 | 0.108 |
|  |  | 55-59 | 1249.4 | 0.035 | 88.2 | 0.100 |
|  |  | 60-64 | 1796.2 | 0.034 | 138.1 | 0.100 |
|  |  | 65-69 | 2333.5 | 0.032 | 200.8 | 0.083 |
|  |  | 70-74 | 2769.0 | 0.034 | 269.6 | 0.083 |
|  |  | 75-79 | 3009.7 | 0.041 | 331.9 | 0.069 |
|  |  | 80-84 | 2793.6 | 0.050 | 375.6 | 0.069 |
|  |  | 85-89 | 2676.4 | 0.071 | 386.8 | 0.061 |
|  |  | 90-94 | 4368.9 | 0.111 | 385.1 | 0.061 |
|  |  | 95+ | 6671.4 | 0.168 | 392.6 | 0.061 |
| Female | Māori | 0-4 | 0.0 | 0.000 | 0.0 | 0.096 |
|  |  | 5-9 | 0.0 | 0.000 | 0.0 | 0.096 |
|  |  | 10-14 | 0.0 | 0.004 | 0.0 | 0.096 |
|  |  | 15-19 | 0.9 | 0.085 | 0.0 | 0.096 |
|  |  | 20-24 | 13.9 | 0.165 | 0.3 | 0.096 |
|  |  | 25-29 | 30.4 | 0.089 | 1.1 | 0.096 |
|  |  | 30-34 | 29.8 | 0.033 | 2.2 | 0.096 |
|  |  | 35-39 | 46.6 | 0.027 | 3.6 | 0.096 |
|  |  | 40-44 | 130.0 | 0.027 | 6.9 | 0.096 |
|  |  | 45-49 | 290.3 | 0.020 | 15.9 | 0.112 |
|  |  | 50-54 | 459.2 | 0.017 | 32.3 | 0.112 |
|  |  | 55-59 | 650.4 | 0.017 | 54.8 | 0.124 |
|  |  | 60-64 | 1053.5 | 0.023 | 87.2 | 0.124 |
|  |  | 65-69 | 1401.6 | 0.027 | 130.0 | 0.110 |
|  |  | 70-74 | 1690.4 | 0.031 | 176.7 | 0.110 |
|  |  | 75-79 | 2245.6 | 0.045 | 224.2 | 0.101 |
|  |  | 80-84 | 2870.6 | 0.069 | 268.4 | 0.101 |
|  |  | 85-89 | 2999.2 | 0.103 | 289.6 | 0.090 |
|  |  | 90-94 | 3085.3 | 0.176 | 262.8 | 0.090 |
|  |  | 95+ | 3996.0 | 0.339 | 171.9 | 0.090 |
| Male | non-Māori | 0-4 | 0.0 | 0.000 | 0.0 | 0.079 |
|  |  | 5-9 | 0.0 | 0.000 | 0.0 | 0.079 |
|  |  | 10-14 | 0.0 | 0.002 | 0.0 | 0.079 |
|  |  | 15-19 | 0.4 | 0.038 | 0.0 | 0.079 |
|  |  | 20-24 | 8.5 | 0.074 | 0.2 | 0.079 |
|  |  | 25-29 | 21.7 | 0.040 | 0.8 | 0.079 |
|  |  | 30-34 | 29.3 | 0.018 | 1.9 | 0.079 |
|  |  | 35-39 | 75.9 | 0.012 | 4.1 | 0.079 |
|  |  | 40-44 | 165.8 | 0.013 | 9.6 | 0.079 |
|  |  | 45-49 | 302.9 | 0.016 | 19.8 | 0.088 |
|  |  | 50-54 | 500.5 | 0.015 | 37.0 | 0.088 |
|  |  | 55-59 | 763.1 | 0.015 | 63.1 | 0.088 |
|  |  | 60-64 | 1109.7 | 0.017 | 99.7 | 0.088 |
|  |  | 65-69 | 1570.9 | 0.018 | 148.7 | 0.086 |
|  |  | 70-74 | 2071.5 | 0.020 | 209.4 | 0.086 |
|  |  | 75-79 | 2683.9 | 0.026 | 277.5 | 0.083 |
|  |  | 80-84 | 3562.0 | 0.042 | 348.3 | 0.083 |
|  |  | 85-89 | 4498.6 | 0.079 | 405.2 | 0.072 |
|  |  | 90-94 | 4944.0 | 0.151 | 410.1 | 0.072 |
|  |  | 95+ | 5201.0 | 0.301 | 296.2 | 0.072 |
| Female | non-Māori | 0-4 | 0.0 | 0.000 | 0.0 | 0.069 |
|  |  | 5-9 | 0.0 | 0.000 | 0.0 | 0.069 |
|  |  | 10-14 | 0.0 | 0.000 | 0.0 | 0.069 |
|  |  | 15-19 | 0.1 | 0.000 | 0.0 | 0.069 |
|  |  | 20-24 | 7.0 | 0.000 | 0.1 | 0.069 |
|  |  | 25-29 | 19.7 | 0.001 | 0.8 | 0.069 |
|  |  | 30-34 | 16.4 | 0.002 | 1.8 | 0.069 |
|  |  | 35-39 | 21.2 | 0.003 | 2.6 | 0.069 |
|  |  | 40-44 | 50.4 | 0.006 | 4.2 | 0.069 |
|  |  | 45-49 | 104.6 | 0.007 | 7.7 | 0.086 |
|  |  | 50-54 | 198.9 | 0.005 | 14.7 | 0.086 |
|  |  | 55-59 | 350.4 | 0.006 | 27.4 | 0.089 |
|  |  | 60-64 | 551.5 | 0.010 | 47.4 | 0.089 |
|  |  | 65-69 | 864.6 | 0.012 | 76.9 | 0.093 |
|  |  | 70-74 | 1258.7 | 0.015 | 118.7 | 0.093 |
|  |  | 75-79 | 1786.0 | 0.021 | 171.8 | 0.093 |
|  |  | 80-84 | 2495.9 | 0.032 | 234.9 | 0.093 |
|  |  | 85-89 | 3131.2 | 0.062 | 294.8 | 0.086 |
|  |  | 90-94 | 3297.2 | 0.144 | 303.7 | 0.086 |
|  |  | 95+ | 3388.0 | 0.344 | 187.0 | 0.086 |

Table S13: Stroke input parameters

| **Sex** | **Ethnic group** | **Age group (years)** | **Incidence rate per 100,000** | **Case-fatality rate** | **Prevalence per 1000** | **Disability rate** |
| --- | --- | --- | --- | --- | --- | --- |
| Male | Māori | 0-4 | 0.0 | 0.000 | 0.0 | 0.396 |
|  |  | 5-9 | 0.0 | 0.006 | 0.0 | 0.396 |
|  |  | 10-14 | 0.0 | 0.067 | 0.0 | 0.396 |
|  |  | 15-19 | 0.0 | 0.122 | 0.0 | 0.396 |
|  |  | 20-24 | 0.0 | 0.089 | 0.0 | 0.396 |
|  |  | 25-29 | 2.1 | 0.042 | 0.2 | 0.396 |
|  |  | 30-34 | 15.1 | 0.089 | 0.4 | 0.396 |
|  |  | 35-39 | 51.8 | 0.074 | 1.6 | 0.396 |
|  |  | 40-44 | 81.2 | 0.035 | 4.4 | 0.396 |
|  |  | 45-49 | 115.5 | 0.024 | 8.3 | 0.132 |
|  |  | 50-54 | 168.0 | 0.023 | 14.1 | 0.132 |
|  |  | 55-59 | 216.4 | 0.023 | 21.5 | 0.143 |
|  |  | 60-64 | 297.6 | 0.020 | 31.0 | 0.143 |
|  |  | 65-69 | 448.8 | 0.019 | 45.3 | 0.218 |
|  |  | 70-74 | 625.7 | 0.035 | 64.0 | 0.218 |
|  |  | 75-79 | 733.8 | 0.058 | 79.7 | 0.294 |
|  |  | 80-84 | 788.9 | 0.083 | 87.6 | 0.294 |
|  |  | 85-89 | 1013.0 | 0.168 | 82.2 | 0.534 |
|  |  | 90-94 | 1645.2 | 0.399 | 57.6 | 0.534 |
|  |  | 95+ | 2760.7 | 0.803 | 36.8 | 0.534 |
| Female | Māori | 0-4 | 0.0 | 0.000 | 0.0 | 0.431 |
|  |  | 5-9 | 0.0 | 0.000 | 0.0 | 0.431 |
|  |  | 10-14 | 0.0 | 0.000 | 0.0 | 0.431 |
|  |  | 15-19 | 0.0 | 0.000 | 0.0 | 0.431 |
|  |  | 20-24 | 0.0 | 0.000 | 0.0 | 0.431 |
|  |  | 25-29 | 0.8 | 0.333 | 0.0 | 0.431 |
|  |  | 30-34 | 4.2 | 1.476 | 0.0 | 0.431 |
|  |  | 35-39 | 15.1 | 1.897 | 0.1 | 0.431 |
|  |  | 40-44 | 75.9 | 1.017 | 0.6 | 0.431 |
|  |  | 45-49 | 163.9 | 0.126 | 4.2 | 0.137 |
|  |  | 50-54 | 226.0 | 0.029 | 12.5 | 0.137 |
|  |  | 55-59 | 274.1 | 0.027 | 22.4 | 0.130 |
|  |  | 60-64 | 353.9 | 0.030 | 33.7 | 0.130 |
|  |  | 65-69 | 463.0 | 0.034 | 47.0 | 0.164 |
|  |  | 70-74 | 586.2 | 0.042 | 61.9 | 0.164 |
|  |  | 75-79 | 721.5 | 0.055 | 76.6 | 0.258 |
|  |  | 80-84 | 1041.1 | 0.096 | 88.4 | 0.258 |
|  |  | 85-89 | 1650.4 | 0.229 | 87.5 | 0.559 |
|  |  | 90-94 | 2168.2 | 0.481 | 60.5 | 0.559 |
|  |  | 95+ | 2670.8 | 0.770 | 37.1 | 0.559 |
| Male | non-Māori | 0-4 | 0.0 | 0.004 | 0.0 | 0.373 |
|  |  | 5-9 | 0.0 | 0.028 | 0.0 | 0.373 |
|  |  | 10-14 | 0.0 | 0.043 | 0.0 | 0.373 |
|  |  | 15-19 | 0.0 | 0.044 | 0.0 | 0.373 |
|  |  | 20-24 | 0.0 | 0.050 | 0.0 | 0.373 |
|  |  | 25-29 | 5.1 | 0.051 | 0.3 | 0.373 |
|  |  | 30-34 | 5.1 | 0.028 | 0.5 | 0.373 |
|  |  | 35-39 | 19.9 | 0.012 | 1.0 | 0.373 |
|  |  | 40-44 | 44.5 | 0.012 | 2.5 | 0.373 |
|  |  | 45-49 | 72.9 | 0.014 | 5.1 | 0.132 |
|  |  | 50-54 | 116.6 | 0.012 | 9.3 | 0.132 |
|  |  | 55-59 | 188.1 | 0.013 | 15.9 | 0.122 |
|  |  | 60-64 | 288.9 | 0.015 | 26.0 | 0.122 |
|  |  | 65-69 | 447.7 | 0.017 | 41.0 | 0.150 |
|  |  | 70-74 | 656.4 | 0.020 | 62.5 | 0.150 |
|  |  | 75-79 | 921.0 | 0.029 | 90.1 | 0.158 |
|  |  | 80-84 | 1255.7 | 0.048 | 120.5 | 0.158 |
|  |  | 85-89 | 1589.6 | 0.097 | 142.8 | 0.166 |
|  |  | 90-94 | 1876.4 | 0.219 | 127.9 | 0.166 |
|  |  | 95+ | 2773.8 | 0.472 | 73.9 | 0.166 |
| Female | non-Māori | 0-4 | 0.0 | 0.000 | 0.0 | 0.415 |
|  |  | 5-9 | 0.0 | 0.000 | 0.0 | 0.415 |
|  |  | 10-14 | 0.0 | 0.000 | 0.0 | 0.415 |
|  |  | 15-19 | 0.0 | 0.002 | 0.0 | 0.415 |
|  |  | 20-24 | 0.0 | 0.136 | 0.0 | 0.415 |
|  |  | 25-29 | 3.4 | 0.354 | 0.1 | 0.415 |
|  |  | 30-34 | 10.9 | 0.172 | 0.2 | 0.415 |
|  |  | 35-39 | 23.4 | 0.039 | 0.9 | 0.415 |
|  |  | 40-44 | 39.1 | 0.024 | 2.3 | 0.415 |
|  |  | 45-49 | 57.6 | 0.019 | 4.3 | 0.180 |
|  |  | 50-54 | 78.8 | 0.015 | 7.2 | 0.180 |
|  |  | 55-59 | 111.4 | 0.009 | 11.3 | 0.166 |
|  |  | 60-64 | 182.0 | 0.011 | 17.7 | 0.166 |
|  |  | 65-69 | 299.4 | 0.017 | 27.6 | 0.164 |
|  |  | 70-74 | 483.1 | 0.024 | 42.7 | 0.164 |
|  |  | 75-79 | 775.5 | 0.033 | 64.8 | 0.180 |
|  |  | 80-84 | 1223.1 | 0.054 | 94.2 | 0.180 |
|  |  | 85-89 | 1739.5 | 0.080 | 116.5 | 0.242 |
|  |  | 90-94 | 2120.5 | 0.124 | 124.7 | 0.242 |
|  |  | 95+ | 2777.6 | 0.182 | 109.2 | 0.242 |

Table S14: Lung cancer input parameters

| **Sex** | **Ethnic group** | **Age group (years)** | **Incidence rate per 100,000** | **Case-fatality rate** | **Remission rate** | **Prevalence per 1000** | **Disability rate** |
| --- | --- | --- | --- | --- | --- | --- | --- |
| Male | Māori | 0-4 | 0.0 | 0.00 | 0.000 | 0.0 | 0.323 |
|  |  | 5-9 | 0.0 | 0.00 | 0.000 | 0.0 | 0.323 |
|  |  | 10-14 | 0.0 | 0.00 | 0.000 | 0.0 | 0.323 |
|  |  | 15-19 | 0.0 | 0.00 | 0.000 | 0.0 | 0.323 |
|  |  | 20-24 | 0.1 | 0.00 | 0.000 | 0.0 | 0.323 |
|  |  | 25-29 | 0.5 | 0.03 | 0.005 | 0.0 | 0.323 |
|  |  | 30-34 | 1.3 | 0.14 | 0.023 | 0.0 | 0.323 |
|  |  | 35-39 | 3.4 | 0.30 | 0.046 | 0.1 | 0.323 |
|  |  | 40-44 | 7.9 | 0.29 | 0.063 | 0.1 | 0.323 |
|  |  | 45-49 | 21.8 | 0.27 | 0.065 | 0.4 | 0.323 |
|  |  | 50-54 | 56.5 | 0.51 | 0.058 | 0.9 | 0.323 |
|  |  | 55-59 | 132.3 | 0.79 | 0.055 | 1.3 | 0.323 |
|  |  | 60-64 | 268.6 | 0.80 | 0.048 | 2.7 | 0.323 |
|  |  | 65-69 | 378.4 | 0.83 | 0.052 | 4.1 | 0.281 |
|  |  | 70-74 | 447.6 | 0.95 | 0.056 | 4.5 | 0.281 |
|  |  | 75-79 | 504.9 | 1.31 | 0.052 | 3.9 | 0.281 |
|  |  | 80-84 | 552.0 | 1.67 | 0.046 | 3.2 | 0.281 |
|  |  | 85-89 | 545.2 | 1.59 | 0.044 | 3.4 | 0.281 |
|  |  | 90-94 | 469.1 | 1.62 | 0.043 | 2.9 | 0.281 |
|  |  | 95+ | 404.5 | 1.77 | 0.043 | 2.3 | 0.281 |
| Female | Māori | 0-4 | 0.0 | 0.00 | 0.000 | 0.0 | 0.396 |
|  |  | 5-9 | 0.0 | 0.00 | 0.000 | 0.0 | 0.396 |
|  |  | 10-14 | 0.0 | 0.00 | 0.000 | 0.0 | 0.396 |
|  |  | 15-19 | 0.0 | 0.00 | 0.000 | 0.0 | 0.396 |
|  |  | 20-24 | 0.3 | 0.00 | 0.000 | 0.0 | 0.396 |
|  |  | 25-29 | 1.5 | 0.10 | 0.010 | 0.0 | 0.396 |
|  |  | 30-34 | 3.9 | 0.53 | 0.061 | 0.1 | 0.396 |
|  |  | 35-39 | 8.2 | 1.14 | 0.155 | 0.1 | 0.396 |
|  |  | 40-44 | 15.3 | 1.00 | 0.186 | 0.1 | 0.396 |
|  |  | 45-49 | 42.1 | 0.57 | 0.138 | 0.4 | 0.396 |
|  |  | 50-54 | 105.0 | 0.56 | 0.092 | 1.3 | 0.396 |
|  |  | 55-59 | 208.8 | 0.63 | 0.080 | 2.6 | 0.396 |
|  |  | 60-64 | 353.0 | 0.85 | 0.069 | 3.7 | 0.396 |
|  |  | 65-69 | 443.6 | 0.99 | 0.067 | 4.1 | 0.313 |
|  |  | 70-74 | 502.0 | 1.02 | 0.060 | 4.5 | 0.313 |
|  |  | 75-79 | 549.3 | 0.98 | 0.045 | 5.2 | 0.313 |
|  |  | 80-84 | 557.2 | 0.99 | 0.037 | 5.7 | 0.313 |
|  |  | 85-89 | 502.3 | 1.23 | 0.035 | 4.2 | 0.313 |
|  |  | 90-94 | 432.5 | 1.43 | 0.034 | 3.1 | 0.313 |
|  |  | 95+ | 378.7 | 1.64 | 0.034 | 2.4 | 0.313 |
| Male | non-Māori | 0-4 | 0.0 | 0.00 | 0.000 | 0.0 | 0.358 |
|  |  | 5-9 | 0.0 | 0.00 | 0.000 | 0.0 | 0.358 |
|  |  | 10-14 | 0.0 | 0.00 | 0.000 | 0.0 | 0.358 |
|  |  | 15-19 | 0.0 | 0.00 | 0.000 | 0.0 | 0.358 |
|  |  | 20-24 | 0.1 | 0.00 | 0.000 | 0.0 | 0.358 |
|  |  | 25-29 | 0.3 | 0.07 | 0.014 | 0.0 | 0.358 |
|  |  | 30-34 | 0.6 | 0.29 | 0.062 | 0.0 | 0.358 |
|  |  | 35-39 | 1.6 | 0.33 | 0.080 | 0.0 | 0.358 |
|  |  | 40-44 | 3.6 | 0.35 | 0.082 | 0.1 | 0.358 |
|  |  | 45-49 | 9.0 | 0.47 | 0.078 | 0.1 | 0.331 |
|  |  | 50-54 | 20.9 | 0.55 | 0.066 | 0.3 | 0.331 |
|  |  | 55-59 | 43.6 | 0.55 | 0.068 | 0.6 | 0.338 |
|  |  | 60-64 | 85.6 | 0.61 | 0.066 | 1.1 | 0.338 |
|  |  | 65-69 | 135.1 | 0.71 | 0.073 | 1.6 | 0.297 |
|  |  | 70-74 | 199.0 | 0.83 | 0.082 | 2.1 | 0.297 |
|  |  | 75-79 | 268.5 | 1.11 | 0.091 | 2.3 | 0.273 |
|  |  | 80-84 | 309.2 | 1.55 | 0.105 | 1.9 | 0.273 |
|  |  | 85-89 | 298.5 | 1.96 | 0.112 | 1.5 | 0.378 |
|  |  | 90-94 | 275.5 | 2.23 | 0.105 | 1.2 | 0.378 |
|  |  | 95+ | 245.2 | 2.46 | 0.100 | 1.0 | 0.378 |
| Female | non-Māori | 0-4 | 0.0 | 0.00 | 0.000 | 0.0 | 0.353 |
|  |  | 5-9 | 0.0 | 0.00 | 0.000 | 0.0 | 0.353 |
|  |  | 10-14 | 0.0 | 0.00 | 0.000 | 0.0 | 0.353 |
|  |  | 15-19 | 0.0 | 0.00 | 0.000 | 0.0 | 0.353 |
|  |  | 20-24 | 0.2 | 0.00 | 0.000 | 0.0 | 0.353 |
|  |  | 25-29 | 0.6 | 0.03 | 0.019 | 0.0 | 0.353 |
|  |  | 30-34 | 1.3 | 0.12 | 0.094 | 0.1 | 0.353 |
|  |  | 35-39 | 2.7 | 0.15 | 0.179 | 0.1 | 0.353 |
|  |  | 40-44 | 5.9 | 0.26 | 0.149 | 0.1 | 0.353 |
|  |  | 45-49 | 11.9 | 0.39 | 0.079 | 0.2 | 0.355 |
|  |  | 50-54 | 22.9 | 0.44 | 0.076 | 0.4 | 0.355 |
|  |  | 55-59 | 42.6 | 0.50 | 0.079 | 0.6 | 0.367 |
|  |  | 60-64 | 73.9 | 0.55 | 0.067 | 1.0 | 0.367 |
|  |  | 65-69 | 106.9 | 0.68 | 0.063 | 1.4 | 0.305 |
|  |  | 70-74 | 139.5 | 0.80 | 0.062 | 1.6 | 0.305 |
|  |  | 75-79 | 162.6 | 0.97 | 0.058 | 1.6 | 0.290 |
|  |  | 80-84 | 171.7 | 1.14 | 0.055 | 1.4 | 0.290 |
|  |  | 85-89 | 169.9 | 1.13 | 0.053 | 1.5 | 0.122 |
|  |  | 90-94 | 147.7 | 1.19 | 0.052 | 1.2 | 0.122 |
|  |  | 95+ | 128.4 | 1.36 | 0.052 | 1.0 | 0.122 |

Table S15: Head and neck cancer input parameters

| **Sex** | **Ethnic group** | **Age group (years)** | **Incidence rate per 100,000** | **Case-fatality rate** | **Remission rate** | **Prevalence per 1000** | **Disability rate** |
| --- | --- | --- | --- | --- | --- | --- | --- |
| Male | Māori | 0-4 | 0.0 | 0.000 | 0.000 | 0.0 | 0.319 |
|  |  | 5-9 | 0.0 | 0.000 | 0.000 | 0.0 | 0.319 |
|  |  | 10-14 | 0.0 | 0.000 | 0.000 | 0.0 | 0.319 |
|  |  | 15-19 | 0.3 | 0.000 | 0.000 | 0.0 | 0.319 |
|  |  | 20-24 | 0.9 | 0.001 | 0.000 | 0.0 | 0.319 |
|  |  | 25-29 | 1.8 | 0.010 | 0.031 | 0.1 | 0.319 |
|  |  | 30-34 | 3.1 | 0.033 | 0.132 | 0.1 | 0.319 |
|  |  | 35-39 | 5.1 | 0.033 | 0.145 | 0.2 | 0.319 |
|  |  | 40-44 | 8.4 | 0.035 | 0.084 | 0.3 | 0.319 |
|  |  | 45-49 | 13.4 | 0.054 | 0.061 | 0.6 | 0.319 |
|  |  | 50-54 | 20.1 | 0.075 | 0.097 | 0.9 | 0.319 |
|  |  | 55-59 | 28.9 | 0.097 | 0.111 | 1.1 | 0.319 |
|  |  | 60-64 | 37.9 | 0.144 | 0.086 | 1.4 | 0.319 |
|  |  | 65-69 | 42.8 | 0.159 | 0.064 | 1.7 | 0.270 |
|  |  | 70-74 | 44.7 | 0.181 | 0.052 | 1.9 | 0.270 |
|  |  | 75-79 | 44.1 | 0.192 | 0.055 | 1.8 | 0.270 |
|  |  | 80-84 | 45.4 | 0.177 | 0.065 | 1.8 | 0.270 |
|  |  | 85-89 | 56.9 | 0.208 | 0.059 | 2.0 | 0.270 |
|  |  | 90-94 | 73.4 | 0.282 | 0.056 | 2.1 | 0.270 |
|  |  | 95+ | 88.3 | 0.344 | 0.060 | 2.2 | 0.270 |
| Female | Māori | 0-4 | 0.0 | 0.000 | 0.000 | 0.0 | 0.388 |
|  |  | 5-9 | 0.0 | 0.000 | 0.000 | 0.0 | 0.388 |
|  |  | 10-14 | 0.0 | 0.000 | 0.000 | 0.0 | 0.388 |
|  |  | 15-19 | 0.1 | 0.000 | 0.000 | 0.0 | 0.388 |
|  |  | 20-24 | 0.3 | 0.001 | 0.000 | 0.0 | 0.388 |
|  |  | 25-29 | 0.9 | 0.013 | 0.092 | 0.0 | 0.388 |
|  |  | 30-34 | 2.5 | 0.047 | 0.395 | 0.1 | 0.388 |
|  |  | 35-39 | 3.8 | 0.047 | 0.452 | 0.1 | 0.388 |
|  |  | 40-44 | 5.1 | 0.046 | 0.263 | 0.1 | 0.388 |
|  |  | 45-49 | 6.9 | 0.073 | 0.142 | 0.2 | 0.388 |
|  |  | 50-54 | 9.1 | 0.105 | 0.164 | 0.3 | 0.388 |
|  |  | 55-59 | 12.2 | 0.142 | 0.151 | 0.4 | 0.388 |
|  |  | 60-64 | 16.1 | 0.209 | 0.104 | 0.5 | 0.388 |
|  |  | 65-69 | 19.0 | 0.235 | 0.086 | 0.5 | 0.196 |
|  |  | 70-74 | 21.9 | 0.283 | 0.085 | 0.6 | 0.196 |
|  |  | 75-79 | 24.1 | 0.312 | 0.123 | 0.6 | 0.196 |
|  |  | 80-84 | 27.3 | 0.283 | 0.178 | 0.6 | 0.196 |
|  |  | 85-89 | 35.7 | 0.322 | 0.169 | 0.7 | 0.196 |
|  |  | 90-94 | 46.5 | 0.473 | 0.152 | 0.7 | 0.196 |
|  |  | 95+ | 58.9 | 0.695 | 0.130 | 0.7 | 0.196 |
| Male | non-Māori | 0-4 | 0.0 | 0.000 | 0.000 | 0.0 | 0.280 |
|  |  | 5-9 | 0.0 | 0.000 | 0.000 | 0.0 | 0.280 |
|  |  | 10-14 | 0.0 | 0.000 | 0.000 | 0.0 | 0.280 |
|  |  | 15-19 | 0.0 | 0.000 | 0.000 | 0.0 | 0.280 |
|  |  | 20-24 | 0.7 | 0.001 | 0.000 | 0.0 | 0.280 |
|  |  | 25-29 | 1.1 | 0.009 | 0.012 | 0.1 | 0.280 |
|  |  | 30-34 | 1.8 | 0.026 | 0.051 | 0.1 | 0.280 |
|  |  | 35-39 | 2.9 | 0.028 | 0.063 | 0.2 | 0.280 |
|  |  | 40-44 | 4.9 | 0.030 | 0.037 | 0.3 | 0.280 |
|  |  | 45-49 | 8.1 | 0.048 | 0.019 | 0.5 | 0.256 |
|  |  | 50-54 | 12.9 | 0.067 | 0.040 | 0.7 | 0.256 |
|  |  | 55-59 | 19.6 | 0.083 | 0.065 | 1.0 | 0.283 |
|  |  | 60-64 | 27.8 | 0.115 | 0.054 | 1.3 | 0.283 |
|  |  | 65-69 | 33.3 | 0.129 | 0.052 | 1.6 | 0.225 |
|  |  | 70-74 | 37.4 | 0.153 | 0.057 | 1.7 | 0.225 |
|  |  | 75-79 | 40.2 | 0.165 | 0.080 | 1.7 | 0.182 |
|  |  | 80-84 | 44.4 | 0.152 | 0.108 | 1.7 | 0.182 |
|  |  | 85-89 | 56.5 | 0.178 | 0.098 | 1.9 | 0.112 |
|  |  | 90-94 | 71.2 | 0.249 | 0.101 | 2.0 | 0.112 |
|  |  | 95+ | 81.3 | 0.332 | 0.115 | 2.0 | 0.112 |
| Female | non-Māori | 0-4 | 0.0 | 0.000 | 0.000 | 0.0 | 0.494 |
|  |  | 5-9 | 0.0 | 0.000 | 0.000 | 0.0 | 0.494 |
|  |  | 10-14 | 0.0 | 0.000 | 0.000 | 0.0 | 0.494 |
|  |  | 15-19 | 0.0 | 0.000 | 0.000 | 0.0 | 0.494 |
|  |  | 20-24 | 0.3 | 0.000 | 0.000 | 0.0 | 0.494 |
|  |  | 25-29 | 0.6 | 0.010 | 0.051 | 0.0 | 0.494 |
|  |  | 30-34 | 1.4 | 0.040 | 0.216 | 0.0 | 0.494 |
|  |  | 35-39 | 2.1 | 0.043 | 0.245 | 0.1 | 0.494 |
|  |  | 40-44 | 3.2 | 0.038 | 0.174 | 0.1 | 0.494 |
|  |  | 45-49 | 4.6 | 0.050 | 0.136 | 0.2 | 0.386 |
|  |  | 50-54 | 6.3 | 0.070 | 0.140 | 0.2 | 0.386 |
|  |  | 55-59 | 9.1 | 0.089 | 0.138 | 0.3 | 0.312 |
|  |  | 60-64 | 12.6 | 0.107 | 0.136 | 0.4 | 0.312 |
|  |  | 65-69 | 15.7 | 0.137 | 0.137 | 0.5 | 0.263 |
|  |  | 70-74 | 19.1 | 0.177 | 0.150 | 0.6 | 0.263 |
|  |  | 75-79 | 21.8 | 0.221 | 0.189 | 0.6 | 0.297 |
|  |  | 80-84 | 25.4 | 0.258 | 0.237 | 0.5 | 0.297 |
|  |  | 85-89 | 34.8 | 0.291 | 0.256 | 0.6 | 0.165 |
|  |  | 90-94 | 44.4 | 0.341 | 0.259 | 0.7 | 0.165 |
|  |  | 95+ | 49.3 | 0.389 | 0.264 | 0.8 | 0.165 |

Table S16: Esophageal cancer input parameters

| **Sex** | **Ethnic group** | **Age group (years)** | **Incidence rate per 100,000** | **Case-fatality rate** | **Remission rate** | **Prevalence per 1000** | **Disability rate** |
| --- | --- | --- | --- | --- | --- | --- | --- |
| Male | Māori | 0-4 | 0.0 | 0.00 | 0.000 | 0.000 | 0.283 |
|  |  | 5-9 | 0.0 | 0.00 | 0.000 | 0.000 | 0.283 |
|  |  | 10-14 | 0.0 | 0.00 | 0.000 | 0.000 | 0.283 |
|  |  | 15-19 | 0.0 | 0.00 | 0.000 | 0.000 | 0.283 |
|  |  | 20-24 | 0.0 | 0.00 | 0.000 | 0.000 | 0.283 |
|  |  | 25-29 | 0.0 | 0.08 | 0.009 | 0.000 | 0.283 |
|  |  | 30-34 | 0.0 | 0.33 | 0.032 | 0.001 | 0.283 |
|  |  | 35-39 | 0.5 | 0.38 | 0.023 | 0.007 | 0.283 |
|  |  | 40-44 | 1.3 | 0.43 | 0.048 | 0.022 | 0.283 |
|  |  | 45-49 | 2.9 | 0.60 | 0.129 | 0.036 | 0.283 |
|  |  | 50-54 | 6.9 | 0.63 | 0.166 | 0.072 | 0.283 |
|  |  | 55-59 | 15.7 | 0.53 | 0.114 | 0.195 | 0.283 |
|  |  | 60-64 | 27.9 | 0.76 | 0.096 | 0.304 | 0.283 |
|  |  | 65-69 | 39.4 | 0.73 | 0.075 | 0.438 | 0.283 |
|  |  | 70-74 | 50.9 | 0.69 | 0.042 | 0.652 | 0.283 |
|  |  | 75-79 | 62.3 | 0.82 | 0.021 | 0.736 | 0.283 |
|  |  | 80-84 | 70.9 | 0.97 | 0.017 | 0.728 | 0.283 |
|  |  | 85-89 | 72.6 | 1.22 | 0.016 | 0.619 | 0.283 |
|  |  | 90-94 | 64.1 | 1.58 | 0.016 | 0.430 | 0.283 |
|  |  | 95+ | 51.9 | 1.78 | 0.016 | 0.282 | 0.283 |
| Female | Māori | 0-4 | 0.0 | 0.00 | 0.000 | 0.000 | 0.271 |
|  |  | 5-9 | 0.0 | 0.00 | 0.000 | 0.000 | 0.271 |
|  |  | 10-14 | 0.0 | 0.00 | 0.000 | 0.000 | 0.271 |
|  |  | 15-19 | 0.0 | 0.00 | 0.000 | 0.000 | 0.271 |
|  |  | 20-24 | 0.0 | 0.00 | 0.000 | 0.000 | 0.271 |
|  |  | 25-29 | 0.0 | 0.06 | 0.006 | 0.000 | 0.271 |
|  |  | 30-34 | 0.0 | 0.24 | 0.021 | 0.000 | 0.271 |
|  |  | 35-39 | 0.2 | 0.26 | 0.020 | 0.003 | 0.271 |
|  |  | 40-44 | 0.4 | 0.28 | 0.017 | 0.010 | 0.271 |
|  |  | 45-49 | 0.9 | 0.40 | 0.026 | 0.017 | 0.271 |
|  |  | 50-54 | 1.8 | 0.45 | 0.030 | 0.029 | 0.271 |
|  |  | 55-59 | 4.3 | 0.41 | 0.020 | 0.072 | 0.271 |
|  |  | 60-64 | 8.7 | 0.51 | 0.019 | 0.138 | 0.271 |
|  |  | 65-69 | 13.0 | 0.50 | 0.017 | 0.215 | 0.271 |
|  |  | 70-74 | 17.8 | 0.50 | 0.012 | 0.314 | 0.271 |
|  |  | 75-79 | 23.7 | 0.58 | 0.008 | 0.383 | 0.271 |
|  |  | 80-84 | 28.0 | 0.66 | 0.008 | 0.416 | 0.271 |
|  |  | 85-89 | 27.0 | 0.77 | 0.008 | 0.371 | 0.271 |
|  |  | 90-94 | 22.1 | 0.93 | 0.008 | 0.262 | 0.271 |
|  |  | 95+ | 17.4 | 1.07 | 0.008 | 0.162 | 0.271 |
| Male | non-Māori | 0-4 | 0.0 | 0.00 | 0.000 | 0.000 | 0.530 |
|  |  | 5-9 | 0.0 | 0.00 | 0.000 | 0.000 | 0.530 |
|  |  | 10-14 | 0.0 | 0.00 | 0.000 | 0.000 | 0.530 |
|  |  | 15-19 | 0.0 | 0.00 | 0.000 | 0.000 | 0.530 |
|  |  | 20-24 | 0.0 | 0.00 | 0.000 | 0.000 | 0.530 |
|  |  | 25-29 | 0.0 | 0.11 | 0.024 | 0.000 | 0.530 |
|  |  | 30-34 | 0.1 | 0.46 | 0.094 | 0.000 | 0.530 |
|  |  | 35-39 | 0.5 | 0.50 | 0.084 | 0.006 | 0.530 |
|  |  | 40-44 | 1.3 | 0.55 | 0.140 | 0.017 | 0.530 |
|  |  | 45-49 | 2.7 | 0.84 | 0.250 | 0.023 | 0.530 |
|  |  | 50-54 | 5.6 | 0.94 | 0.237 | 0.042 | 0.530 |
|  |  | 55-59 | 11.8 | 0.76 | 0.128 | 0.113 | 0.530 |
|  |  | 60-64 | 21.4 | 0.97 | 0.106 | 0.187 | 0.530 |
|  |  | 65-69 | 31.8 | 0.96 | 0.107 | 0.275 | 0.295 |
|  |  | 70-74 | 43.5 | 0.97 | 0.092 | 0.392 | 0.295 |
|  |  | 75-79 | 56.2 | 1.15 | 0.079 | 0.450 | 0.295 |
|  |  | 80-84 | 65.8 | 1.31 | 0.075 | 0.473 | 0.295 |
|  |  | 85-89 | 65.9 | 1.55 | 0.072 | 0.420 | 0.295 |
|  |  | 90-94 | 55.6 | 1.82 | 0.069 | 0.310 | 0.295 |
|  |  | 95+ | 44.3 | 1.98 | 0.071 | 0.210 | 0.295 |
| Female | non-Māori | 0-4 | 0.0 | 0.00 | 0.000 | 0.000 | 0.367 |
|  |  | 5-9 | 0.0 | 0.00 | 0.000 | 0.000 | 0.367 |
|  |  | 10-14 | 0.0 | 0.00 | 0.000 | 0.000 | 0.367 |
|  |  | 15-19 | 0.0 | 0.00 | 0.000 | 0.000 | 0.367 |
|  |  | 20-24 | 0.0 | 0.00 | 0.000 | 0.000 | 0.367 |
|  |  | 25-29 | 0.0 | 0.09 | 0.025 | 0.000 | 0.367 |
|  |  | 30-34 | 0.0 | 0.39 | 0.099 | 0.000 | 0.367 |
|  |  | 35-39 | 0.3 | 0.42 | 0.082 | 0.003 | 0.367 |
|  |  | 40-44 | 0.6 | 0.42 | 0.068 | 0.010 | 0.367 |
|  |  | 45-49 | 1.0 | 0.57 | 0.082 | 0.014 | 0.367 |
|  |  | 50-54 | 1.9 | 0.61 | 0.063 | 0.023 | 0.367 |
|  |  | 55-59 | 4.0 | 0.50 | 0.039 | 0.057 | 0.367 |
|  |  | 60-64 | 7.8 | 0.62 | 0.040 | 0.103 | 0.367 |
|  |  | 65-69 | 11.9 | 0.64 | 0.043 | 0.155 | 0.279 |
|  |  | 70-74 | 17.1 | 0.68 | 0.045 | 0.221 | 0.279 |
|  |  | 75-79 | 24.7 | 0.85 | 0.052 | 0.264 | 0.279 |
|  |  | 80-84 | 31.8 | 1.04 | 0.058 | 0.289 | 0.279 |
|  |  | 85-89 | 33.0 | 1.25 | 0.061 | 0.261 | 0.279 |
|  |  | 90-94 | 29.1 | 1.53 | 0.058 | 0.194 | 0.279 |
|  |  | 95+ | 24.1 | 1.73 | 0.058 | 0.133 | 0.279 |

Table S17: Stomach cancer input parameters

| **Sex** | **Ethnic group** | **Age group (years)** | **Incidence rate per 100,000** | **Case-fatality rate** | **Remission rate** | **Prevalence per 1000** | **Disability rate** |
| --- | --- | --- | --- | --- | --- | --- | --- |
| Male | Māori | 0-4 | 0.0 | 0.000 | 0.000 | 0.0 | 0.262 |
|  |  | 5-9 | 0.0 | 0.000 | 0.000 | 0.0 | 0.262 |
|  |  | 10-14 | 0.0 | 0.000 | 0.000 | 0.0 | 0.262 |
|  |  | 15-19 | 0.0 | 0.000 | 0.000 | 0.1 | 0.262 |
|  |  | 20-24 | 0.7 | 0.000 | 0.000 | 0.1 | 0.262 |
|  |  | 25-29 | 2.0 | 0.025 | 0.086 | 0.2 | 0.262 |
|  |  | 30-34 | 3.4 | 0.126 | 0.347 | 0.1 | 0.262 |
|  |  | 35-39 | 6.4 | 0.267 | 0.305 | 0.1 | 0.262 |
|  |  | 40-44 | 11.5 | 0.477 | 0.176 | 0.2 | 0.262 |
|  |  | 45-49 | 17.6 | 0.529 | 0.146 | 0.2 | 0.262 |
|  |  | 50-54 | 24.6 | 0.410 | 0.153 | 0.4 | 0.262 |
|  |  | 55-59 | 37.3 | 0.528 | 0.189 | 0.5 | 0.262 |
|  |  | 60-64 | 60.1 | 0.828 | 0.162 | 0.6 | 0.262 |
|  |  | 65-69 | 83.9 | 0.802 | 0.119 | 0.8 | 0.326 |
|  |  | 70-74 | 103.7 | 0.665 | 0.099 | 1.3 | 0.326 |
|  |  | 75-79 | 139.4 | 0.553 | 0.082 | 1.9 | 0.326 |
|  |  | 80-84 | 185.0 | 0.464 | 0.064 | 3.1 | 0.326 |
|  |  | 85-89 | 209.0 | 0.573 | 0.066 | 3.4 | 0.326 |
|  |  | 90-94 | 250.4 | 0.777 | 0.060 | 3.1 | 0.326 |
|  |  | 95+ | 280.6 | 0.949 | 0.057 | 2.9 | 0.326 |
| Female | Māori | 0-4 | 0.0 | 0.000 | 0.000 | 0.0 | 0.296 |
|  |  | 5-9 | 0.0 | 0.000 | 0.000 | 0.0 | 0.296 |
|  |  | 10-14 | 0.0 | 0.000 | 0.000 | 0.0 | 0.296 |
|  |  | 15-19 | 0.0 | 0.000 | 0.000 | 0.1 | 0.296 |
|  |  | 20-24 | 0.2 | 0.000 | 0.000 | 0.1 | 0.296 |
|  |  | 25-29 | 0.9 | 0.111 | 0.011 | 0.1 | 0.296 |
|  |  | 30-34 | 4.8 | 0.484 | 0.047 | 0.1 | 0.296 |
|  |  | 35-39 | 8.2 | 0.605 | 0.058 | 0.1 | 0.296 |
|  |  | 40-44 | 11.3 | 0.500 | 0.075 | 0.2 | 0.296 |
|  |  | 45-49 | 15.7 | 0.406 | 0.079 | 0.3 | 0.296 |
|  |  | 50-54 | 20.0 | 0.424 | 0.084 | 0.4 | 0.296 |
|  |  | 55-59 | 24.1 | 0.496 | 0.142 | 0.4 | 0.296 |
|  |  | 60-64 | 35.8 | 0.416 | 0.123 | 0.5 | 0.296 |
|  |  | 65-69 | 48.6 | 0.307 | 0.100 | 1.0 | 0.281 |
|  |  | 70-74 | 61.3 | 0.403 | 0.097 | 1.3 | 0.281 |
|  |  | 75-79 | 61.2 | 0.518 | 0.090 | 1.1 | 0.281 |
|  |  | 80-84 | 69.5 | 0.448 | 0.069 | 1.1 | 0.281 |
|  |  | 85-89 | 123.1 | 0.404 | 0.050 | 2.2 | 0.281 |
|  |  | 90-94 | 148.8 | 0.523 | 0.046 | 2.7 | 0.281 |
|  |  | 95+ | 163.8 | 0.720 | 0.044 | 2.4 | 0.281 |
| Male | non-Māori | 0-4 | 0.0 | 0.000 | 0.000 | 0.0 | 0.304 |
|  |  | 5-9 | 0.0 | 0.000 | 0.000 | 0.0 | 0.304 |
|  |  | 10-14 | 0.0 | 0.000 | 0.000 | 0.0 | 0.304 |
|  |  | 15-19 | 0.0 | 0.000 | 0.000 | 0.0 | 0.304 |
|  |  | 20-24 | 0.2 | 0.000 | 0.000 | 0.0 | 0.304 |
|  |  | 25-29 | 0.3 | 0.030 | 0.014 | 0.0 | 0.304 |
|  |  | 30-34 | 0.7 | 0.136 | 0.065 | 0.0 | 0.304 |
|  |  | 35-39 | 1.4 | 0.213 | 0.102 | 0.0 | 0.304 |
|  |  | 40-44 | 2.5 | 0.266 | 0.138 | 0.1 | 0.304 |
|  |  | 45-49 | 4.6 | 0.286 | 0.156 | 0.1 | 0.300 |
|  |  | 50-54 | 8.5 | 0.298 | 0.163 | 0.2 | 0.300 |
|  |  | 55-59 | 15.6 | 0.391 | 0.210 | 0.2 | 0.359 |
|  |  | 60-64 | 26.4 | 0.500 | 0.254 | 0.3 | 0.359 |
|  |  | 65-69 | 36.5 | 0.491 | 0.243 | 0.5 | 0.342 |
|  |  | 70-74 | 52.4 | 0.561 | 0.223 | 0.6 | 0.342 |
|  |  | 75-79 | 71.3 | 0.665 | 0.192 | 0.8 | 0.249 |
|  |  | 80-84 | 87.4 | 0.660 | 0.147 | 1.0 | 0.249 |
|  |  | 85-89 | 102.4 | 0.652 | 0.133 | 1.3 | 0.143 |
|  |  | 90-94 | 114.0 | 0.683 | 0.131 | 1.4 | 0.143 |
|  |  | 95+ | 115.8 | 0.724 | 0.132 | 1.4 | 0.143 |
| Female | non-Māori | 0-4 | 0.0 | 0.000 | 0.000 | 0.0 | 0.153 |
|  |  | 5-9 | 0.0 | 0.000 | 0.000 | 0.0 | 0.153 |
|  |  | 10-14 | 0.0 | 0.000 | 0.000 | 0.0 | 0.153 |
|  |  | 15-19 | 0.0 | 0.000 | 0.000 | 0.0 | 0.153 |
|  |  | 20-24 | 0.5 | 0.000 | 0.000 | 0.0 | 0.153 |
|  |  | 25-29 | 0.3 | 0.032 | 0.019 | 0.0 | 0.153 |
|  |  | 30-34 | 0.4 | 0.148 | 0.086 | 0.0 | 0.153 |
|  |  | 35-39 | 0.8 | 0.249 | 0.118 | 0.0 | 0.153 |
|  |  | 40-44 | 1.7 | 0.302 | 0.134 | 0.0 | 0.153 |
|  |  | 45-49 | 2.9 | 0.289 | 0.133 | 0.1 | 0.312 |
|  |  | 50-54 | 4.5 | 0.265 | 0.121 | 0.1 | 0.312 |
|  |  | 55-59 | 6.6 | 0.345 | 0.147 | 0.1 | 0.239 |
|  |  | 60-64 | 9.4 | 0.457 | 0.172 | 0.1 | 0.239 |
|  |  | 65-69 | 14.6 | 0.425 | 0.167 | 0.2 | 0.317 |
|  |  | 70-74 | 23.1 | 0.446 | 0.156 | 0.3 | 0.317 |
|  |  | 75-79 | 33.0 | 0.522 | 0.137 | 0.5 | 0.231 |
|  |  | 80-84 | 41.5 | 0.512 | 0.110 | 0.6 | 0.231 |
|  |  | 85-89 | 47.4 | 0.491 | 0.101 | 0.8 | 0.107 |
|  |  | 90-94 | 51.3 | 0.516 | 0.100 | 0.8 | 0.107 |
|  |  | 95+ | 51.4 | 0.561 | 0.101 | 0.8 | 0.107 |

Table S18: Liver cancer input parameters

| **Sex** | **Ethnic group** | **Age group (years)** | **Incidence rate per 100,000** | **Case-fatality rate** | **Remission rate** | **Prevalence per 1000** | **Disability rate** |
| --- | --- | --- | --- | --- | --- | --- | --- |
| Male | Māori | 0-4 | 0.0 | 0.00 | 0.000 | 0.000 | 0.148 |
|  |  | 5-9 | 0.0 | 0.00 | 0.000 | 0.000 | 0.148 |
|  |  | 10-14 | 0.0 | 0.00 | 0.000 | 0.000 | 0.148 |
|  |  | 15-19 | 0.0 | 0.00 | 0.000 | 0.005 | 0.148 |
|  |  | 20-24 | 0.7 | 0.00 | 0.000 | 0.027 | 0.148 |
|  |  | 25-29 | 1.4 | 0.03 | 0.031 | 0.072 | 0.148 |
|  |  | 30-34 | 2.9 | 0.14 | 0.133 | 0.100 | 0.148 |
|  |  | 35-39 | 5.3 | 0.17 | 0.150 | 0.131 | 0.148 |
|  |  | 40-44 | 9.8 | 0.26 | 0.109 | 0.216 | 0.148 |
|  |  | 45-49 | 18.3 | 0.47 | 0.087 | 0.298 | 0.148 |
|  |  | 50-54 | 32.4 | 0.63 | 0.086 | 0.409 | 0.148 |
|  |  | 55-59 | 49.8 | 0.62 | 0.086 | 0.628 | 0.148 |
|  |  | 60-64 | 59.4 | 0.55 | 0.083 | 0.867 | 0.148 |
|  |  | 65-69 | 57.2 | 0.54 | 0.086 | 0.931 | 0.138 |
|  |  | 70-74 | 52.6 | 0.56 | 0.103 | 0.832 | 0.138 |
|  |  | 75-79 | 53.1 | 0.67 | 0.097 | 0.727 | 0.138 |
|  |  | 80-84 | 54.2 | 1.16 | 0.072 | 0.512 | 0.138 |
|  |  | 85-89 | 44.5 | 1.72 | 0.087 | 0.263 | 0.138 |
|  |  | 90-94 | 23.7 | 1.18 | 0.117 | 0.192 | 0.138 |
|  |  | 95+ | 13.3 | 0.48 | 0.141 | 0.187 | 0.138 |
| Female | Māori | 0-4 | 0.0 | 0.00 | 0.000 | 0.000 | 0.183 |
|  |  | 5-9 | 0.0 | 0.00 | 0.000 | 0.000 | 0.183 |
|  |  | 10-14 | 0.0 | 0.00 | 0.000 | 0.000 | 0.183 |
|  |  | 15-19 | 0.0 | 0.00 | 0.000 | 0.000 | 0.183 |
|  |  | 20-24 | 0.1 | 0.00 | 0.000 | 0.003 | 0.183 |
|  |  | 25-29 | 0.6 | 0.13 | 0.041 | 0.017 | 0.183 |
|  |  | 30-34 | 1.1 | 0.54 | 0.174 | 0.018 | 0.183 |
|  |  | 35-39 | 1.8 | 0.62 | 0.200 | 0.020 | 0.183 |
|  |  | 40-44 | 3.3 | 0.51 | 0.150 | 0.041 | 0.183 |
|  |  | 45-49 | 4.2 | 0.48 | 0.122 | 0.065 | 0.183 |
|  |  | 50-54 | 5.8 | 0.49 | 0.122 | 0.085 | 0.183 |
|  |  | 55-59 | 10.4 | 0.50 | 0.123 | 0.144 | 0.183 |
|  |  | 60-64 | 13.7 | 0.67 | 0.129 | 0.172 | 0.183 |
|  |  | 65-69 | 18.3 | 0.92 | 0.115 | 0.175 | 0.183 |
|  |  | 70-74 | 20.4 | 1.28 | 0.118 | 0.152 | 0.183 |
|  |  | 75-79 | 19.5 | 1.31 | 0.102 | 0.136 | 0.183 |
|  |  | 80-84 | 18.3 | 0.98 | 0.050 | 0.171 | 0.183 |
|  |  | 85-89 | 16.1 | 0.89 | 0.039 | 0.177 | 0.183 |
|  |  | 90-94 | 10.3 | 0.77 | 0.038 | 0.140 | 0.183 |
|  |  | 95+ | 6.4 | 0.55 | 0.039 | 0.102 | 0.183 |
| Male | non-Māori | 0-4 | 0.0 | 0.00 | 0.000 | 0.000 | 0.147 |
|  |  | 5-9 | 0.0 | 0.00 | 0.000 | 0.000 | 0.147 |
|  |  | 10-14 | 0.0 | 0.00 | 0.000 | 0.000 | 0.147 |
|  |  | 15-19 | 0.0 | 0.00 | 0.000 | 0.000 | 0.147 |
|  |  | 20-24 | 0.1 | 0.00 | 0.000 | 0.002 | 0.147 |
|  |  | 25-29 | 0.5 | 0.09 | 0.029 | 0.013 | 0.147 |
|  |  | 30-34 | 0.9 | 0.42 | 0.126 | 0.017 | 0.147 |
|  |  | 35-39 | 1.4 | 0.53 | 0.146 | 0.019 | 0.147 |
|  |  | 40-44 | 2.4 | 0.42 | 0.103 | 0.036 | 0.147 |
|  |  | 45-49 | 4.2 | 0.44 | 0.079 | 0.069 | 0.178 |
|  |  | 50-54 | 7.4 | 0.59 | 0.092 | 0.098 | 0.178 |
|  |  | 55-59 | 11.8 | 0.59 | 0.121 | 0.149 | 0.178 |
|  |  | 60-64 | 15.8 | 0.65 | 0.157 | 0.190 | 0.178 |
|  |  | 65-69 | 21.3 | 0.89 | 0.151 | 0.205 | 0.217 |
|  |  | 70-74 | 28.8 | 1.35 | 0.159 | 0.196 | 0.217 |
|  |  | 75-79 | 33.2 | 2.12 | 0.167 | 0.152 | 0.190 |
|  |  | 80-84 | 34.3 | 2.56 | 0.136 | 0.126 | 0.190 |
|  |  | 85-89 | 37.2 | 1.94 | 0.114 | 0.173 | 0.190 |
|  |  | 90-94 | 33.0 | 1.43 | 0.123 | 0.211 | 0.190 |
|  |  | 95+ | 27.8 | 1.17 | 0.145 | 0.206 | 0.190 |
| Female | non-Māori | 0-4 | 0.0 | 0.00 | 0.000 | 0.000 | 0.005 |
|  |  | 5-9 | 0.0 | 0.00 | 0.000 | 0.000 | 0.005 |
|  |  | 10-14 | 0.0 | 0.00 | 0.000 | 0.000 | 0.005 |
|  |  | 15-19 | 0.0 | 0.00 | 0.000 | 0.000 | 0.005 |
|  |  | 20-24 | 9.4 | 0.00 | 0.000 | 0.431 | 0.005 |
|  |  | 25-29 | 2.2 | 0.77 | 0.246 | 0.341 | 0.005 |
|  |  | 30-34 | 0.4 | 3.02 | 0.982 | 0.002 | 0.005 |
|  |  | 35-39 | 0.5 | 2.04 | 0.752 | 0.002 | 0.005 |
|  |  | 40-44 | 0.9 | 0.78 | 0.259 | 0.008 | 0.005 |
|  |  | 45-49 | 1.5 | 0.76 | 0.109 | 0.015 | 0.262 |
|  |  | 50-54 | 2.3 | 0.56 | 0.059 | 0.031 | 0.262 |
|  |  | 55-59 | 3.9 | 0.58 | 0.105 | 0.052 | 0.262 |
|  |  | 60-64 | 6.9 | 0.87 | 0.221 | 0.062 | 0.262 |
|  |  | 65-69 | 9.6 | 1.27 | 0.257 | 0.063 | 0.174 |
|  |  | 70-74 | 13.3 | 1.50 | 0.215 | 0.075 | 0.174 |
|  |  | 75-79 | 17.1 | 1.71 | 0.241 | 0.087 | 0.183 |
|  |  | 80-84 | 18.0 | 1.98 | 0.367 | 0.078 | 0.183 |
|  |  | 85-89 | 14.8 | 1.82 | 0.491 | 0.065 | 0.183 |
|  |  | 90-94 | 9.1 | 0.94 | 0.634 | 0.059 | 0.183 |
|  |  | 95+ | 6.5 | 0.35 | 0.731 | 0.058 | 0.183 |

Table S19: Pancreatic cancer input parameters

| **Sex** | **Ethnic group** | **Age group (years)** | **Incidence rate per 100,000** | **Case-fatality rate** | **Remission rate** | **Prevalence per 1000** | **Disability rate** |
| --- | --- | --- | --- | --- | --- | --- | --- |
| Male | Māori | 0-4 | 0.0 | 0.00 | 0.000 | 0.000 | 0.160 |
|  |  | 5-9 | 0.0 | 0.00 | 0.000 | 0.000 | 0.160 |
|  |  | 10-14 | 0.0 | 0.00 | 0.000 | 0.000 | 0.160 |
|  |  | 15-19 | 0.0 | 0.00 | 0.006 | 0.000 | 0.160 |
|  |  | 20-24 | 0.1 | 0.01 | 0.338 | 0.002 | 0.160 |
|  |  | 25-29 | 0.1 | 0.08 | 0.865 | 0.001 | 0.160 |
|  |  | 30-34 | 0.6 | 0.25 | 0.365 | 0.007 | 0.160 |
|  |  | 35-39 | 1.3 | 0.41 | 0.049 | 0.021 | 0.160 |
|  |  | 40-44 | 2.5 | 0.53 | 0.049 | 0.038 | 0.160 |
|  |  | 45-49 | 4.8 | 0.61 | 0.075 | 0.062 | 0.160 |
|  |  | 50-54 | 8.9 | 0.63 | 0.096 | 0.105 | 0.160 |
|  |  | 55-59 | 16.3 | 0.63 | 0.065 | 0.199 | 0.160 |
|  |  | 60-64 | 27.5 | 0.62 | 0.044 | 0.361 | 0.160 |
|  |  | 65-69 | 41.0 | 0.69 | 0.033 | 0.524 | 0.160 |
|  |  | 70-74 | 58.0 | 0.79 | 0.022 | 0.670 | 0.160 |
|  |  | 75-79 | 76.5 | 0.83 | 0.014 | 0.853 | 0.160 |
|  |  | 80-84 | 94.9 | 0.82 | 0.012 | 1.079 | 0.160 |
|  |  | 85-89 | 114.3 | 0.79 | 0.012 | 1.357 | 0.160 |
|  |  | 90-94 | 126.8 | 0.87 | 0.012 | 1.447 | 0.160 |
|  |  | 95+ | 132.5 | 0.97 | 0.012 | 1.370 | 0.160 |
| Female | Māori | 0-4 | 0.0 | 0.00 | 0.000 | 0.000 | 0.183 |
|  |  | 5-9 | 0.0 | 0.00 | 0.000 | 0.000 | 0.183 |
|  |  | 10-14 | 0.0 | 0.00 | 0.000 | 0.000 | 0.183 |
|  |  | 15-19 | 0.0 | 0.00 | 0.006 | 0.000 | 0.183 |
|  |  | 20-24 | 0.1 | 0.03 | 0.335 | 0.001 | 0.183 |
|  |  | 25-29 | 0.1 | 0.12 | 0.857 | 0.001 | 0.183 |
|  |  | 30-34 | 0.4 | 0.25 | 0.355 | 0.005 | 0.183 |
|  |  | 35-39 | 0.9 | 0.38 | 0.037 | 0.016 | 0.183 |
|  |  | 40-44 | 2.2 | 0.53 | 0.039 | 0.031 | 0.183 |
|  |  | 45-49 | 4.2 | 0.70 | 0.047 | 0.051 | 0.183 |
|  |  | 50-54 | 7.2 | 0.79 | 0.050 | 0.077 | 0.183 |
|  |  | 55-59 | 13.3 | 0.75 | 0.040 | 0.145 | 0.183 |
|  |  | 60-64 | 23.1 | 0.70 | 0.031 | 0.274 | 0.183 |
|  |  | 65-69 | 35.4 | 0.77 | 0.026 | 0.413 | 0.183 |
|  |  | 70-74 | 50.3 | 0.87 | 0.020 | 0.538 | 0.183 |
|  |  | 75-79 | 71.2 | 0.96 | 0.016 | 0.693 | 0.183 |
|  |  | 80-84 | 94.7 | 1.13 | 0.018 | 0.812 | 0.183 |
|  |  | 85-89 | 111.3 | 1.33 | 0.022 | 0.828 | 0.183 |
|  |  | 90-94 | 120.8 | 1.48 | 0.023 | 0.808 | 0.183 |
|  |  | 95+ | 124.4 | 1.65 | 0.023 | 0.753 | 0.183 |
| Male | non-Māori | 0-4 | 0.0 | 0.00 | 0.000 | 0.000 | 0.314 |
|  |  | 5-9 | 0.0 | 0.00 | 0.000 | 0.000 | 0.314 |
|  |  | 10-14 | 0.0 | 0.00 | 0.000 | 0.000 | 0.314 |
|  |  | 15-19 | 0.0 | 0.00 | 0.006 | 0.000 | 0.314 |
|  |  | 20-24 | 0.0 | 0.04 | 0.338 | 0.001 | 0.314 |
|  |  | 25-29 | 0.0 | 0.15 | 0.868 | 0.000 | 0.314 |
|  |  | 30-34 | 0.2 | 0.35 | 0.368 | 0.002 | 0.314 |
|  |  | 35-39 | 0.5 | 0.57 | 0.054 | 0.007 | 0.314 |
|  |  | 40-44 | 1.2 | 0.79 | 0.065 | 0.013 | 0.314 |
|  |  | 45-49 | 2.5 | 0.98 | 0.170 | 0.020 | 0.314 |
|  |  | 50-54 | 4.9 | 1.10 | 0.281 | 0.033 | 0.314 |
|  |  | 55-59 | 9.4 | 1.10 | 0.220 | 0.065 | 0.314 |
|  |  | 60-64 | 16.6 | 1.11 | 0.173 | 0.119 | 0.314 |
|  |  | 65-69 | 25.4 | 1.32 | 0.138 | 0.168 | 0.192 |
|  |  | 70-74 | 36.5 | 1.61 | 0.093 | 0.207 | 0.192 |
|  |  | 75-79 | 47.9 | 1.77 | 0.065 | 0.254 | 0.192 |
|  |  | 80-84 | 58.5 | 1.85 | 0.068 | 0.300 | 0.192 |
|  |  | 85-89 | 70.3 | 2.06 | 0.076 | 0.327 | 0.192 |
|  |  | 90-94 | 81.8 | 2.39 | 0.075 | 0.331 | 0.192 |
|  |  | 95+ | 87.1 | 2.63 | 0.074 | 0.326 | 0.192 |
| Female | non-Māori | 0-4 | 0.0 | 0.00 | 0.000 | 0.000 | 0.369 |
|  |  | 5-9 | 0.0 | 0.00 | 0.000 | 0.000 | 0.369 |
|  |  | 10-14 | 0.0 | 0.00 | 0.000 | 0.000 | 0.369 |
|  |  | 15-19 | 0.0 | 0.00 | 0.006 | 0.000 | 0.369 |
|  |  | 20-24 | 0.0 | 0.07 | 0.327 | 0.001 | 0.369 |
|  |  | 25-29 | 0.0 | 0.22 | 0.837 | 0.000 | 0.369 |
|  |  | 30-34 | 0.2 | 0.30 | 0.352 | 0.002 | 0.369 |
|  |  | 35-39 | 0.4 | 0.45 | 0.045 | 0.006 | 0.369 |
|  |  | 40-44 | 1.0 | 0.76 | 0.054 | 0.011 | 0.369 |
|  |  | 45-49 | 2.2 | 1.15 | 0.087 | 0.017 | 0.369 |
|  |  | 50-54 | 4.2 | 1.40 | 0.116 | 0.025 | 0.369 |
|  |  | 55-59 | 8.3 | 1.40 | 0.105 | 0.050 | 0.369 |
|  |  | 60-64 | 15.2 | 1.38 | 0.095 | 0.095 | 0.369 |
|  |  | 65-69 | 24.0 | 1.60 | 0.088 | 0.138 | 0.230 |
|  |  | 70-74 | 34.6 | 1.87 | 0.071 | 0.173 | 0.230 |
|  |  | 75-79 | 47.3 | 2.00 | 0.060 | 0.223 | 0.230 |
|  |  | 80-84 | 60.4 | 2.13 | 0.067 | 0.270 | 0.230 |
|  |  | 85-89 | 70.5 | 2.41 | 0.079 | 0.282 | 0.230 |
|  |  | 90-94 | 78.0 | 2.65 | 0.081 | 0.285 | 0.230 |
|  |  | 95+ | 81.8 | 2.84 | 0.082 | 0.283 | 0.230 |

Table S20: Cervical cancer input parameters

| **Sex** | **Ethnic group** | **Age group (years)** | **Incidence rate per 100,000** | **Case-fatality rate** | **Remission rate** | **Prevalence per 1000** | **Disability rate** |
| --- | --- | --- | --- | --- | --- | --- | --- |
| Female | Māori | 0-4 | 0.0 | 0.000 | 0.0 | 0.00 | 0.127 |
|  |  | 5-9 | 0.0 | 0.000 | 0.000 | 0.00 | 0.127 |
|  |  | 10-14 | 0.0 | 0.000 | 0.000 | 0.00 | 0.127 |
|  |  | 15-19 | 0.0 | 0.000 | 0.000 | 0.00 | 0.127 |
|  |  | 20-24 | 2.3 | 0.000 | 0.000 | 0.05 | 0.127 |
|  |  | 25-29 | 7.7 | 0.004 | 0.000 | 0.29 | 0.127 |
|  |  | 30-34 | 13.1 | 0.017 | 0.028 | 0.59 | 0.127 |
|  |  | 35-39 | 18.9 | 0.024 | 0.131 | 0.71 | 0.127 |
|  |  | 40-44 | 21.7 | 0.037 | 0.221 | 0.76 | 0.127 |
|  |  | 45-49 | 21.1 | 0.050 | 0.236 | 0.79 | 0.127 |
|  |  | 50-54 | 20.8 | 0.062 | 0.214 | 0.81 | 0.127 |
|  |  | 55-59 | 22.3 | 0.090 | 0.183 | 0.91 | 0.127 |
|  |  | 60-64 | 20.9 | 0.117 | 0.128 | 0.97 | 0.127 |
|  |  | 65-69 | 17.3 | 0.135 | 0.098 | 0.92 | 0.093 |
|  |  | 70-74 | 13.0 | 0.138 | 0.073 | 0.82 | 0.093 |
|  |  | 75-79 | 9.6 | 0.099 | 0.045 | 0.77 | 0.093 |
|  |  | 80-84 | 9.3 | 0.043 | 0.020 | 0.91 | 0.093 |
|  |  | 85-89 | 11.4 | 0.033 | 0.006 | 1.23 | 0.093 |
|  |  | 90-94 | 6.7 | 0.034 | 0.004 | 1.44 | 0.093 |
|  |  | 95+ | 2.1 | 0.035 | 0.004 | 1.33 | 0.093 |
| Female | non-Māori | 0-4 | 0.0 | 0.000 | 0.000 | 0.00 | 0.209 |
|  |  | 5-9 | 0.0 | 0.000 | 0.000 | 0.00 | 0.209 |
|  |  | 10-14 | 0.0 | 0.000 | 0.000 | 0.00 | 0.209 |
|  |  | 15-19 | 0.0 | 0.000 | 0.000 | 0.00 | 0.209 |
|  |  | 20-24 | 1.7 | 0.001 | 0.000 | 0.04 | 0.209 |
|  |  | 25-29 | 6.2 | 0.006 | 0.050 | 0.21 | 0.209 |
|  |  | 30-34 | 10.1 | 0.018 | 0.230 | 0.37 | 0.209 |
|  |  | 35-39 | 12.7 | 0.040 | 0.347 | 0.35 | 0.209 |
|  |  | 40-44 | 13.2 | 0.034 | 0.352 | 0.33 | 0.209 |
|  |  | 45-49 | 12.1 | 0.015 | 0.304 | 0.36 | 0.130 |
|  |  | 50-54 | 10.8 | 0.025 | 0.242 | 0.39 | 0.130 |
|  |  | 55-59 | 10.1 | 0.057 | 0.183 | 0.41 | 0.107 |
|  |  | 60-64 | 9.8 | 0.085 | 0.157 | 0.41 | 0.107 |
|  |  | 65-69 | 9.2 | 0.104 | 0.147 | 0.39 | 0.119 |
|  |  | 70-74 | 8.5 | 0.149 | 0.126 | 0.35 | 0.119 |
|  |  | 75-79 | 8.0 | 0.202 | 0.084 | 0.30 | 0.109 |
|  |  | 80-84 | 8.0 | 0.212 | 0.049 | 0.29 | 0.109 |
|  |  | 85-89 | 8.7 | 0.175 | 0.057 | 0.33 | 0.056 |
|  |  | 90-94 | 8.7 | 0.123 | 0.088 | 0.38 | 0.056 |
|  |  | 95+ | 8.1 | 0.082 | 0.117 | 0.40 | 0.056 |

Table S21: Bladder cancer input parameters

| **Sex** | **Ethnic group** | **Age group (years)** | **Incidence rate per 100,000** | **Case-fatality rate** | **Remission rate** | **Prevalence per 1000** | **Disability rate** |
| --- | --- | --- | --- | --- | --- | --- | --- |
| Male | Māori | 0-4 | 0.0 | 0.000 | 0.000 | 0.000 | 0.232 |
|  |  | 5-9 | 0.0 | 0.000 | 0.000 | 0.000 | 0.232 |
|  |  | 10-14 | 0.0 | 0.000 | 0.000 | 0.000 | 0.232 |
|  |  | 15-19 | 0.0 | 0.000 | 0.000 | 0.000 | 0.232 |
|  |  | 20-24 | 0.3 | 0.000 | 0.000 | 0.005 | 0.232 |
|  |  | 25-29 | 1.0 | 0.063 | 0.083 | 0.030 | 0.232 |
|  |  | 30-34 | 1.6 | 0.262 | 0.349 | 0.031 | 0.232 |
|  |  | 35-39 | 2.6 | 0.270 | 0.366 | 0.035 | 0.232 |
|  |  | 40-44 | 4.2 | 0.192 | 0.273 | 0.071 | 0.232 |
|  |  | 45-49 | 6.4 | 0.166 | 0.275 | 0.122 | 0.232 |
|  |  | 50-54 | 9.6 | 0.182 | 0.314 | 0.173 | 0.232 |
|  |  | 55-59 | 13.4 | 0.240 | 0.314 | 0.222 | 0.232 |
|  |  | 60-64 | 18.9 | 0.267 | 0.274 | 0.305 | 0.232 |
|  |  | 65-69 | 31.1 | 0.184 | 0.210 | 0.580 | 0.208 |
|  |  | 70-74 | 45.4 | 0.175 | 0.160 | 1.077 | 0.208 |
|  |  | 75-79 | 58.8 | 0.281 | 0.135 | 1.353 | 0.208 |
|  |  | 80-84 | 67.7 | 0.324 | 0.123 | 1.444 | 0.208 |
|  |  | 85-89 | 73.4 | 0.279 | 0.122 | 1.692 | 0.208 |
|  |  | 90-94 | 98.9 | 0.377 | 0.121 | 1.932 | 0.208 |
|  |  | 95+ | 132.3 | 0.554 | 0.117 | 2.079 | 0.208 |
| Female | Māori | 0-4 | 0.0 | 0.000 | 0.000 | 0.000 | 0.163 |
|  |  | 5-9 | 0.0 | 0.000 | 0.000 | 0.000 | 0.163 |
|  |  | 10-14 | 0.0 | 0.000 | 0.000 | 0.000 | 0.163 |
|  |  | 15-19 | 0.0 | 0.000 | 0.000 | 0.000 | 0.163 |
|  |  | 20-24 | 0.2 | 0.000 | 0.000 | 0.003 | 0.163 |
|  |  | 25-29 | 0.6 | 0.054 | 0.000 | 0.020 | 0.163 |
|  |  | 30-34 | 1.0 | 0.245 | 0.000 | 0.036 | 0.163 |
|  |  | 35-39 | 1.5 | 0.347 | 0.007 | 0.038 | 0.163 |
|  |  | 40-44 | 2.2 | 0.326 | 0.066 | 0.048 | 0.163 |
|  |  | 45-49 | 3.2 | 0.291 | 0.168 | 0.064 | 0.163 |
|  |  | 50-54 | 4.9 | 0.305 | 0.206 | 0.084 | 0.163 |
|  |  | 55-59 | 7.7 | 0.343 | 0.163 | 0.131 | 0.163 |
|  |  | 60-64 | 10.6 | 0.353 | 0.232 | 0.169 | 0.163 |
|  |  | 65-69 | 14.6 | 0.265 | 0.200 | 0.256 | 0.163 |
|  |  | 70-74 | 19.8 | 0.208 | 0.178 | 0.431 | 0.163 |
|  |  | 75-79 | 26.2 | 0.262 | 0.251 | 0.511 | 0.163 |
|  |  | 80-84 | 36.0 | 0.545 | 0.307 | 0.455 | 0.163 |
|  |  | 85-89 | 50.1 | 1.130 | 0.264 | 0.369 | 0.163 |
|  |  | 90-94 | 60.8 | 1.624 | 0.175 | 0.339 | 0.163 |
|  |  | 95+ | 63.4 | 1.856 | 0.174 | 0.315 | 0.163 |
| Male | non-Māori | 0-4 | 0.0 | 0.000 | 0.000 | 0.000 | 0.145 |
|  |  | 5-9 | 0.0 | 0.000 | 0.000 | 0.000 | 0.145 |
|  |  | 10-14 | 0.0 | 0.000 | 0.000 | 0.000 | 0.145 |
|  |  | 15-19 | 0.0 | 0.000 | 0.000 | 0.000 | 0.145 |
|  |  | 20-24 | 0.2 | 0.000 | 0.000 | 0.008 | 0.145 |
|  |  | 25-29 | 0.5 | 0.010 | 0.039 | 0.025 | 0.145 |
|  |  | 30-34 | 1.1 | 0.044 | 0.176 | 0.041 | 0.145 |
|  |  | 35-39 | 2.1 | 0.052 | 0.258 | 0.054 | 0.145 |
|  |  | 40-44 | 4.0 | 0.043 | 0.225 | 0.098 | 0.145 |
|  |  | 45-49 | 8.1 | 0.037 | 0.183 | 0.219 | 0.128 |
|  |  | 50-54 | 15.7 | 0.040 | 0.197 | 0.438 | 0.128 |
|  |  | 55-59 | 29.2 | 0.055 | 0.217 | 0.777 | 0.144 |
|  |  | 60-64 | 51.9 | 0.071 | 0.249 | 1.263 | 0.144 |
|  |  | 65-69 | 85.4 | 0.084 | 0.239 | 2.037 | 0.140 |
|  |  | 70-74 | 127.5 | 0.089 | 0.208 | 3.306 | 0.140 |
|  |  | 75-79 | 175.3 | 0.105 | 0.219 | 4.716 | 0.110 |
|  |  | 80-84 | 220.5 | 0.158 | 0.269 | 5.213 | 0.110 |
|  |  | 85-89 | 255.6 | 0.303 | 0.275 | 4.727 | 0.093 |
|  |  | 90-94 | 291.7 | 0.591 | 0.184 | 3.959 | 0.093 |
|  |  | 95+ | 309.9 | 0.812 | 0.145 | 3.351 | 0.093 |
| Female | non-Māori | 0-4 | 0.0 | 0.000 | 0.000 | 0.000 | 0.114 |
|  |  | 5-9 | 0.0 | 0.000 | 0.000 | 0.000 | 0.114 |
|  |  | 10-14 | 0.0 | 0.000 | 0.000 | 0.000 | 0.114 |
|  |  | 15-19 | 0.0 | 0.000 | 0.000 | 0.000 | 0.114 |
|  |  | 20-24 | 0.1 | 0.000 | 0.000 | 0.019 | 0.114 |
|  |  | 25-29 | 0.3 | 0.033 | 0.065 | 0.029 | 0.114 |
|  |  | 30-34 | 0.7 | 0.141 | 0.279 | 0.022 | 0.114 |
|  |  | 35-39 | 1.2 | 0.154 | 0.333 | 0.021 | 0.114 |
|  |  | 40-44 | 2.2 | 0.103 | 0.298 | 0.042 | 0.114 |
|  |  | 45-49 | 3.8 | 0.067 | 0.281 | 0.082 | 0.155 |
|  |  | 50-54 | 6.6 | 0.045 | 0.350 | 0.139 | 0.155 |
|  |  | 55-59 | 11.2 | 0.022 | 0.504 | 0.191 | 0.134 |
|  |  | 60-64 | 16.8 | 0.019 | 0.445 | 0.296 | 0.134 |
|  |  | 65-69 | 23.8 | 0.050 | 0.287 | 0.531 | 0.150 |
|  |  | 70-74 | 33.2 | 0.078 | 0.196 | 0.910 | 0.150 |
|  |  | 75-79 | 44.9 | 0.121 | 0.216 | 1.234 | 0.107 |
|  |  | 80-84 | 58.9 | 0.237 | 0.255 | 1.235 | 0.107 |
|  |  | 85-89 | 72.6 | 0.434 | 0.215 | 1.152 | 0.068 |
|  |  | 90-94 | 79.4 | 0.648 | 0.183 | 1.000 | 0.068 |
|  |  | 95+ | 79.0 | 0.711 | 0.249 | 0.836 | 0.068 |

Table S22: Kidney cancer input parameters

| **Sex** | **Ethnic group** | **Age group (years)** | **Incidence rate per 100,000** | **Case-fatality rate** | **Remission rate** | **Prevalence per 1000** | **Disability rate** |
| --- | --- | --- | --- | --- | --- | --- | --- |
| Male | Māori | 0-4 | 0.0 | 0.000 | 0.000 | 0.000 | 0.265 |
|  |  | 5-9 | 0.0 | 0.000 | 0.000 | 0.000 | 0.265 |
|  |  | 10-14 | 0.0 | 0.000 | 0.000 | 0.000 | 0.265 |
|  |  | 15-19 | 0.1 | 0.000 | 0.000 | 0.002 | 0.265 |
|  |  | 20-24 | 0.3 | 0.000 | 0.000 | 0.013 | 0.265 |
|  |  | 25-29 | 0.7 | 0.003 | 0.039 | 0.033 | 0.265 |
|  |  | 30-34 | 2.1 | 0.016 | 0.169 | 0.068 | 0.265 |
|  |  | 35-39 | 4.1 | 0.044 | 0.210 | 0.115 | 0.265 |
|  |  | 40-44 | 7.3 | 0.058 | 0.222 | 0.192 | 0.265 |
|  |  | 45-49 | 12.7 | 0.093 | 0.240 | 0.304 | 0.265 |
|  |  | 50-54 | 19.6 | 0.147 | 0.225 | 0.444 | 0.265 |
|  |  | 55-59 | 27.3 | 0.153 | 0.204 | 0.634 | 0.265 |
|  |  | 60-64 | 37.3 | 0.170 | 0.193 | 0.892 | 0.265 |
|  |  | 65-69 | 45.1 | 0.230 | 0.216 | 1.009 | 0.111 |
|  |  | 70-74 | 51.2 | 0.256 | 0.186 | 1.078 | 0.111 |
|  |  | 75-79 | 48.6 | 0.257 | 0.130 | 1.240 | 0.111 |
|  |  | 80-84 | 41.6 | 0.272 | 0.115 | 1.138 | 0.111 |
|  |  | 85-89 | 40.1 | 0.274 | 0.111 | 1.080 | 0.111 |
|  |  | 90-94 | 24.8 | 0.284 | 0.115 | 0.825 | 0.111 |
|  |  | 95+ | 14.1 | 0.288 | 0.116 | 0.433 | 0.111 |
| Female | Māori | 0-4 | 0.0 | 0.000 | 0.000 | 0.000 | 0.199 |
|  |  | 5-9 | 0.0 | 0.000 | 0.000 | 0.000 | 0.199 |
|  |  | 10-14 | 0.0 | 0.000 | 0.000 | 0.000 | 0.199 |
|  |  | 15-19 | 0.1 | 0.000 | 0.000 | 0.001 | 0.199 |
|  |  | 20-24 | 0.2 | 0.000 | 0.000 | 0.008 | 0.199 |
|  |  | 25-29 | 0.6 | 0.002 | 0.063 | 0.023 | 0.199 |
|  |  | 30-34 | 1.9 | 0.012 | 0.266 | 0.047 | 0.199 |
|  |  | 35-39 | 3.3 | 0.031 | 0.284 | 0.078 | 0.199 |
|  |  | 40-44 | 5.2 | 0.040 | 0.243 | 0.134 | 0.199 |
|  |  | 45-49 | 8.2 | 0.082 | 0.234 | 0.209 | 0.199 |
|  |  | 50-54 | 12.3 | 0.140 | 0.211 | 0.294 | 0.199 |
|  |  | 55-59 | 16.6 | 0.138 | 0.202 | 0.410 | 0.199 |
|  |  | 60-64 | 20.6 | 0.165 | 0.163 | 0.549 | 0.199 |
|  |  | 65-69 | 23.0 | 0.216 | 0.149 | 0.620 | 0.118 |
|  |  | 70-74 | 24.8 | 0.235 | 0.115 | 0.661 | 0.118 |
|  |  | 75-79 | 25.0 | 0.263 | 0.087 | 0.726 | 0.118 |
|  |  | 80-84 | 23.0 | 0.379 | 0.093 | 0.587 | 0.118 |
|  |  | 85-89 | 19.9 | 0.540 | 0.085 | 0.380 | 0.118 |
|  |  | 90-94 | 14.4 | 0.608 | 0.113 | 0.234 | 0.118 |
|  |  | 95+ | 9.4 | 0.658 | 0.145 | 0.131 | 0.118 |
| Male | non-Māori | 0-4 | 0.0 | 0.000 | 0.000 | 0.000 | 0.131 |
|  |  | 5-9 | 0.0 | 0.000 | 0.000 | 0.000 | 0.131 |
|  |  | 10-14 | 0.0 | 0.000 | 0.000 | 0.000 | 0.131 |
|  |  | 15-19 | 0.0 | 0.000 | 0.000 | 0.000 | 0.131 |
|  |  | 20-24 | 0.4 | 0.000 | 0.000 | 0.014 | 0.131 |
|  |  | 25-29 | 0.7 | 0.002 | 0.023 | 0.039 | 0.131 |
|  |  | 30-34 | 1.5 | 0.012 | 0.101 | 0.070 | 0.131 |
|  |  | 35-39 | 3.2 | 0.027 | 0.129 | 0.119 | 0.131 |
|  |  | 40-44 | 6.0 | 0.037 | 0.156 | 0.208 | 0.131 |
|  |  | 45-49 | 11.1 | 0.055 | 0.189 | 0.333 | 0.146 |
|  |  | 50-54 | 18.6 | 0.083 | 0.196 | 0.516 | 0.146 |
|  |  | 55-59 | 28.4 | 0.093 | 0.208 | 0.758 | 0.164 |
|  |  | 60-64 | 42.3 | 0.112 | 0.229 | 1.066 | 0.164 |
|  |  | 65-69 | 54.5 | 0.162 | 0.302 | 1.186 | 0.207 |
|  |  | 70-74 | 66.3 | 0.193 | 0.316 | 1.230 | 0.207 |
|  |  | 75-79 | 72.5 | 0.233 | 0.308 | 1.352 | 0.170 |
|  |  | 80-84 | 74.6 | 0.380 | 0.382 | 1.102 | 0.170 |
|  |  | 85-89 | 78.2 | 0.602 | 0.475 | 0.775 | 0.172 |
|  |  | 90-94 | 57.7 | 0.679 | 0.500 | 0.534 | 0.172 |
|  |  | 95+ | 39.8 | 0.687 | 0.500 | 0.327 | 0.172 |
| Female | non-Māori | 0-4 | 0.0 | 0.000 | 0.000 | 0.000 | 0.144 |
|  |  | 5-9 | 0.0 | 0.000 | 0.000 | 0.000 | 0.144 |
|  |  | 10-14 | 0.0 | 0.000 | 0.000 | 0.000 | 0.144 |
|  |  | 15-19 | 0.0 | 0.000 | 0.000 | 0.000 | 0.144 |
|  |  | 20-24 | 0.2 | 0.000 | 0.000 | 0.009 | 0.144 |
|  |  | 25-29 | 0.5 | 0.002 | 0.030 | 0.026 | 0.144 |
|  |  | 30-34 | 1.2 | 0.010 | 0.128 | 0.048 | 0.144 |
|  |  | 35-39 | 2.2 | 0.026 | 0.154 | 0.079 | 0.144 |
|  |  | 40-44 | 3.8 | 0.036 | 0.170 | 0.130 | 0.144 |
|  |  | 45-49 | 7.1 | 0.055 | 0.200 | 0.206 | 0.179 |
|  |  | 50-54 | 12.1 | 0.083 | 0.206 | 0.323 | 0.179 |
|  |  | 55-59 | 17.3 | 0.090 | 0.208 | 0.469 | 0.180 |
|  |  | 60-64 | 23.9 | 0.101 | 0.203 | 0.657 | 0.180 |
|  |  | 65-69 | 28.2 | 0.136 | 0.233 | 0.757 | 0.132 |
|  |  | 70-74 | 32.7 | 0.156 | 0.222 | 0.802 | 0.132 |
|  |  | 75-79 | 34.0 | 0.181 | 0.201 | 0.891 | 0.166 |
|  |  | 80-84 | 31.5 | 0.262 | 0.249 | 0.730 | 0.166 |
|  |  | 85-89 | 30.0 | 0.364 | 0.316 | 0.497 | 0.124 |
|  |  | 90-94 | 21.3 | 0.390 | 0.345 | 0.333 | 0.124 |
|  |  | 95+ | 14.8 | 0.395 | 0.356 | 0.201 | 0.124 |

Table S23: Endometrial cancer input parameters

| **Sex** | **Ethnic group** | **Age group (years)** | **Incidence rate per 100,000** | **Case-fatality rate** | **Remission rate** | **Prevalence per 1000** | **Disability rate** |
| --- | --- | --- | --- | --- | --- | --- | --- |
| Female | Māori | 0-4 | 0.0 | 0.000 | 0.000 | 0.00 | 0.231 |
|  |  | 5-9 | 0.0 | 0.000 | 0.000 | 0.00 | 0.231 |
|  |  | 10-14 | 0.0 | 0.000 | 0.000 | 0.00 | 0.231 |
|  |  | 15-19 | 0.0 | 0.000 | 0.000 | 0.00 | 0.231 |
|  |  | 20-24 | 0.3 | 0.000 | 0.000 | 0.01 | 0.231 |
|  |  | 25-29 | 1.6 | 0.007 | 0.031 | 0.05 | 0.231 |
|  |  | 30-34 | 4.5 | 0.034 | 0.149 | 0.14 | 0.231 |
|  |  | 35-39 | 8.2 | 0.058 | 0.260 | 0.22 | 0.231 |
|  |  | 40-44 | 15.2 | 0.061 | 0.284 | 0.34 | 0.231 |
|  |  | 45-49 | 29.1 | 0.058 | 0.237 | 0.67 | 0.212 |
|  |  | 50-54 | 49.6 | 0.062 | 0.189 | 1.33 | 0.212 |
|  |  | 55-59 | 69.4 | 0.062 | 0.186 | 2.17 | 0.212 |
|  |  | 60-64 | 75.8 | 0.066 | 0.210 | 2.63 | 0.212 |
|  |  | 65-69 | 72.3 | 0.096 | 0.216 | 2.53 | 0.161 |
|  |  | 70-74 | 75.1 | 0.160 | 0.223 | 2.16 | 0.161 |
|  |  | 75-79 | 79.7 | 0.210 | 0.206 | 1.94 | 0.102 |
|  |  | 80-84 | 76.7 | 0.216 | 0.162 | 1.98 | 0.102 |
|  |  | 85-89 | 69.8 | 0.210 | 0.149 | 2.00 | 0.102 |
|  |  | 90-94 | 54.4 | 0.217 | 0.153 | 1.71 | 0.102 |
|  |  | 95+ | 42.6 | 0.225 | 0.158 | 1.21 | 0.102 |
| Female | non-Māori | 0-4 | 0.0 | 0.000 | 0.000 | 0.00 | 0.176 |
|  |  | 5-9 | 0.0 | 0.000 | 0.000 | 0.00 | 0.176 |
|  |  | 10-14 | 0.0 | 0.000 | 0.000 | 0.00 | 0.176 |
|  |  | 15-19 | 0.0 | 0.000 | 0.000 | 0.00 | 0.176 |
|  |  | 20-24 | 0.1 | 0.000 | 0.000 | 0.01 | 0.176 |
|  |  | 25-29 | 0.3 | 0.005 | 0.009 | 0.02 | 0.176 |
|  |  | 30-34 | 1.8 | 0.019 | 0.050 | 0.06 | 0.176 |
|  |  | 35-39 | 3.5 | 0.022 | 0.116 | 0.14 | 0.176 |
|  |  | 40-44 | 6.7 | 0.024 | 0.128 | 0.25 | 0.176 |
|  |  | 45-49 | 12.8 | 0.031 | 0.097 | 0.49 | 0.187 |
|  |  | 50-54 | 22.5 | 0.035 | 0.093 | 0.92 | 0.187 |
|  |  | 55-59 | 35.6 | 0.034 | 0.121 | 1.51 | 0.179 |
|  |  | 60-64 | 46.8 | 0.041 | 0.159 | 1.99 | 0.179 |
|  |  | 65-69 | 54.8 | 0.066 | 0.171 | 2.21 | 0.153 |
|  |  | 70-74 | 59.0 | 0.096 | 0.203 | 2.14 | 0.153 |
|  |  | 75-79 | 59.9 | 0.120 | 0.207 | 1.90 | 0.144 |
|  |  | 80-84 | 58.0 | 0.139 | 0.180 | 1.87 | 0.144 |
|  |  | 85-89 | 53.1 | 0.162 | 0.220 | 1.61 | 0.070 |
|  |  | 90-94 | 47.8 | 0.201 | 0.253 | 1.21 | 0.070 |
|  |  | 95+ | 43.6 | 0.130 | 0.168 | 0.56 | 0.070 |

Table S24: Melanoma input parameters

| **Sex** | **Ethnic group** | **Age group (years)** | **Incidence rate per 100,000** | **Case-fatality rate** | **Remission rate** | **Prevalence per 1000** | **Disability rate** |
| --- | --- | --- | --- | --- | --- | --- | --- |
| Male | Māori | 0-4 | 0.0 | 0.000 | 0.000 | 0.00 | 0.080 |
|  |  | 5-9 | 0.0 | 0.000 | 0.000 | 0.00 | 0.080 |
|  |  | 10-14 | 0.0 | 0.000 | 0.000 | 0.00 | 0.080 |
|  |  | 15-19 | 0.0 | 0.000 | 0.000 | 0.00 | 0.080 |
|  |  | 20-24 | 1.7 | 0.001 | 0.000 | 0.06 | 0.080 |
|  |  | 25-29 | 3.0 | 0.008 | 0.044 | 0.17 | 0.080 |
|  |  | 30-34 | 4.7 | 0.022 | 0.190 | 0.22 | 0.080 |
|  |  | 35-39 | 6.1 | 0.035 | 0.229 | 0.22 | 0.080 |
|  |  | 40-44 | 7.8 | 0.038 | 0.222 | 0.26 | 0.080 |
|  |  | 45-49 | 10.6 | 0.036 | 0.216 | 0.34 | 0.080 |
|  |  | 50-54 | 14.5 | 0.038 | 0.213 | 0.47 | 0.080 |
|  |  | 55-59 | 19.8 | 0.047 | 0.277 | 0.57 | 0.080 |
|  |  | 60-64 | 28.1 | 0.063 | 0.351 | 0.64 | 0.080 |
|  |  | 65-69 | 37.0 | 0.089 | 0.368 | 0.75 | 0.061 |
|  |  | 70-74 | 46.5 | 0.101 | 0.358 | 0.92 | 0.061 |
|  |  | 75-79 | 57.6 | 0.107 | 0.363 | 1.14 | 0.061 |
|  |  | 80-84 | 66.9 | 0.116 | 0.393 | 1.28 | 0.061 |
|  |  | 85-89 | 67.0 | 0.102 | 0.444 | 1.26 | 0.061 |
|  |  | 90-94 | 59.0 | 0.076 | 0.491 | 1.11 | 0.061 |
|  |  | 95+ | 52.9 | 0.054 | 0.519 | 0.94 | 0.061 |
| Female | Māori | 0-4 | 0.0 | 0.000 | 0.000 | 0.00 | 0.181 |
|  |  | 5-9 | 0.0 | 0.000 | 0.000 | 0.00 | 0.181 |
|  |  | 10-14 | 0.0 | 0.000 | 0.000 | 0.00 | 0.181 |
|  |  | 15-19 | 0.0 | 0.000 | 0.000 | 0.00 | 0.181 |
|  |  | 20-24 | 1.6 | 0.001 | 0.000 | 0.06 | 0.181 |
|  |  | 25-29 | 3.4 | 0.007 | 0.078 | 0.16 | 0.181 |
|  |  | 30-34 | 7.0 | 0.020 | 0.335 | 0.20 | 0.181 |
|  |  | 35-39 | 9.1 | 0.031 | 0.409 | 0.20 | 0.181 |
|  |  | 40-44 | 10.7 | 0.033 | 0.368 | 0.24 | 0.181 |
|  |  | 45-49 | 12.7 | 0.031 | 0.313 | 0.31 | 0.181 |
|  |  | 50-54 | 14.9 | 0.033 | 0.258 | 0.43 | 0.181 |
|  |  | 55-59 | 17.6 | 0.042 | 0.273 | 0.52 | 0.181 |
|  |  | 60-64 | 21.3 | 0.056 | 0.284 | 0.58 | 0.181 |
|  |  | 65-69 | 25.3 | 0.079 | 0.256 | 0.68 | 0.199 |
|  |  | 70-74 | 29.5 | 0.089 | 0.221 | 0.84 | 0.199 |
|  |  | 75-79 | 34.3 | 0.095 | 0.205 | 1.03 | 0.199 |
|  |  | 80-84 | 37.3 | 0.110 | 0.227 | 1.12 | 0.199 |
|  |  | 85-89 | 35.0 | 0.112 | 0.291 | 0.98 | 0.199 |
|  |  | 90-94 | 29.8 | 0.096 | 0.346 | 0.77 | 0.199 |
|  |  | 95+ | 25.6 | 0.080 | 0.388 | 0.58 | 0.199 |
| Male | non-Māori | 0-4 | 0.0 | 0.000 | 0.000 | 0.00 | 0.111 |
|  |  | 5-9 | 0.0 | 0.000 | 0.000 | 0.00 | 0.111 |
|  |  | 10-14 | 0.0 | 0.000 | 0.000 | 0.00 | 0.111 |
|  |  | 15-19 | 0.0 | 0.000 | 0.000 | 0.00 | 0.111 |
|  |  | 20-24 | 11.0 | 0.001 | 0.000 | 0.42 | 0.111 |
|  |  | 25-29 | 14.8 | 0.006 | 0.021 | 1.07 | 0.111 |
|  |  | 30-34 | 20.4 | 0.012 | 0.092 | 1.49 | 0.111 |
|  |  | 35-39 | 27.8 | 0.017 | 0.119 | 1.69 | 0.111 |
|  |  | 40-44 | 38.4 | 0.018 | 0.133 | 2.02 | 0.111 |
|  |  | 45-49 | 54.9 | 0.018 | 0.139 | 2.56 | 0.150 |
|  |  | 50-54 | 78.2 | 0.019 | 0.145 | 3.52 | 0.150 |
|  |  | 55-59 | 109.9 | 0.024 | 0.207 | 4.30 | 0.161 |
|  |  | 60-64 | 157.1 | 0.032 | 0.270 | 4.82 | 0.161 |
|  |  | 65-69 | 205.2 | 0.045 | 0.285 | 5.60 | 0.170 |
|  |  | 70-74 | 252.0 | 0.051 | 0.278 | 6.83 | 0.170 |
|  |  | 75-79 | 301.5 | 0.055 | 0.280 | 8.22 | 0.129 |
|  |  | 80-84 | 339.6 | 0.077 | 0.302 | 8.96 | 0.129 |
|  |  | 85-89 | 340.2 | 0.126 | 0.335 | 8.08 | 0.137 |
|  |  | 90-94 | 315.2 | 0.202 | 0.345 | 6.41 | 0.137 |
|  |  | 95+ | 292.1 | 0.278 | 0.354 | 4.84 | 0.137 |
| Female | non-Māori | 0-4 | 0.0 | 0.000 | 0.000 | 0.00 | 0.166 |
|  |  | 5-9 | 0.0 | 0.000 | 0.000 | 0.00 | 0.166 |
|  |  | 10-14 | 0.0 | 0.000 | 0.000 | 0.00 | 0.166 |
|  |  | 15-19 | 0.0 | 0.000 | 0.000 | 0.00 | 0.166 |
|  |  | 20-24 | 11.6 | 0.001 | 0.000 | 0.41 | 0.166 |
|  |  | 25-29 | 20.3 | 0.005 | 0.046 | 1.16 | 0.166 |
|  |  | 30-34 | 32.0 | 0.011 | 0.197 | 1.52 | 0.166 |
|  |  | 35-39 | 42.3 | 0.015 | 0.246 | 1.52 | 0.166 |
|  |  | 40-44 | 53.2 | 0.017 | 0.238 | 1.80 | 0.166 |
|  |  | 45-49 | 66.3 | 0.016 | 0.211 | 2.31 | 0.167 |
|  |  | 50-54 | 80.5 | 0.017 | 0.176 | 3.20 | 0.167 |
|  |  | 55-59 | 96.9 | 0.021 | 0.202 | 3.91 | 0.128 |
|  |  | 60-64 | 117.5 | 0.028 | 0.215 | 4.38 | 0.128 |
|  |  | 65-69 | 137.5 | 0.039 | 0.193 | 5.12 | 0.126 |
|  |  | 70-74 | 155.8 | 0.044 | 0.166 | 6.24 | 0.126 |
|  |  | 75-79 | 172.2 | 0.048 | 0.151 | 7.50 | 0.098 |
|  |  | 80-84 | 179.5 | 0.056 | 0.169 | 8.14 | 0.098 |
|  |  | 85-89 | 167.7 | 0.059 | 0.236 | 7.00 | 0.096 |
|  |  | 90-94 | 145.2 | 0.055 | 0.300 | 5.10 | 0.096 |
|  |  | 95+ | 127.1 | 0.051 | 0.347 | 3.51 | 0.096 |

Table S25: Thyroid cancer input parameters

| **Sex** | **Ethnic group** | **Age group (years)** | **Incidence rate per 100,000** | **Case-fatality rate** | **Remission rate** | **Prevalence per 1000** | **Disability rate** |
| --- | --- | --- | --- | --- | --- | --- | --- |
| Male | Māori | 0-4 | 0.0 | 0.0000 | 0.00 | 0.000 | 0.135 |
|  |  | 5-9 | 0.0 | 0.0000 | 0.00 | 0.000 | 0.135 |
|  |  | 10-14 | 0.0 | 0.0000 | 0.00 | 0.000 | 0.135 |
|  |  | 15-19 | 0.6 | 0.0000 | 0.00 | 0.014 | 0.135 |
|  |  | 20-24 | 1.5 | 0.0001 | 0.00 | 0.070 | 0.135 |
|  |  | 25-29 | 1.4 | 0.0000 | 0.02 | 0.138 | 0.135 |
|  |  | 30-34 | 2.2 | 0.0000 | 0.10 | 0.176 | 0.135 |
|  |  | 35-39 | 3.3 | 0.0002 | 0.14 | 0.196 | 0.135 |
|  |  | 40-44 | 4.3 | 0.0018 | 0.14 | 0.236 | 0.135 |
|  |  | 45-49 | 5.4 | 0.0100 | 0.15 | 0.284 | 0.135 |
|  |  | 50-54 | 6.9 | 0.0190 | 0.18 | 0.318 | 0.135 |
|  |  | 55-59 | 8.8 | 0.0160 | 0.23 | 0.342 | 0.135 |
|  |  | 60-64 | 10.7 | 0.0110 | 0.29 | 0.361 | 0.135 |
|  |  | 65-69 | 12.4 | 0.0122 | 0.44 | 0.312 | 0.135 |
|  |  | 70-74 | 13.4 | 0.0129 | 0.53 | 0.254 | 0.135 |
|  |  | 75-79 | 12.5 | 0.0105 | 0.64 | 0.215 | 0.135 |
|  |  | 80-84 | 11.6 | 0.0099 | 0.72 | 0.161 | 0.135 |
|  |  | 85-89 | 13.5 | 0.0127 | 0.49 | 0.227 | 0.135 |
|  |  | 90-94 | 14.3 | 0.0151 | 0.48 | 0.286 | 0.135 |
|  |  | 95+ | 14.6 | 0.0137 | 0.57 | 0.259 | 0.135 |
| Female | Māori | 0-4 | 0.0 | 0.0000 | 0.00 | 0.000 | 0.169 |
|  |  | 5-9 | 0.0 | 0.0000 | 0.00 | 0.000 | 0.169 |
|  |  | 10-14 | 0.0 | 0.0000 | 0.00 | 0.001 | 0.169 |
|  |  | 15-19 | 1.5 | 0.0000 | 0.00 | 0.033 | 0.169 |
|  |  | 20-24 | 3.8 | 0.0000 | 0.00 | 0.172 | 0.169 |
|  |  | 25-29 | 4.9 | 0.0000 | 0.05 | 0.354 | 0.169 |
|  |  | 30-34 | 10.6 | 0.0000 | 0.22 | 0.444 | 0.169 |
|  |  | 35-39 | 14.7 | 0.0001 | 0.28 | 0.479 | 0.169 |
|  |  | 40-44 | 17.1 | 0.0011 | 0.25 | 0.584 | 0.169 |
|  |  | 45-49 | 19.4 | 0.0063 | 0.24 | 0.702 | 0.169 |
|  |  | 50-54 | 22.3 | 0.0138 | 0.25 | 0.783 | 0.169 |
|  |  | 55-59 | 24.5 | 0.0150 | 0.26 | 0.846 | 0.169 |
|  |  | 60-64 | 24.5 | 0.0094 | 0.28 | 0.874 | 0.169 |
|  |  | 65-69 | 25.7 | 0.0100 | 0.38 | 0.752 | 0.145 |
|  |  | 70-74 | 26.3 | 0.0105 | 0.43 | 0.625 | 0.145 |
|  |  | 75-79 | 24.5 | 0.0089 | 0.55 | 0.521 | 0.145 |
|  |  | 80-84 | 23.1 | 0.0188 | 0.80 | 0.321 | 0.145 |
|  |  | 85-89 | 25.6 | 0.0399 | 0.91 | 0.271 | 0.145 |
|  |  | 90-94 | 27.0 | 0.0429 | 1.05 | 0.252 | 0.145 |
|  |  | 95+ | 27.6 | 0.0342 | 1.27 | 0.217 | 0.145 |
| Male | non-Māori | 0-4 | 0.0 | 0.0000 | 0.00 | 0.000 | 0.137 |
|  |  | 5-9 | 0.0 | 0.0000 | 0.00 | 0.000 | 0.137 |
|  |  | 10-14 | 0.0 | 0.0001 | 0.00 | 0.000 | 0.137 |
|  |  | 15-19 | 0.4 | 0.0000 | 0.00 | 0.009 | 0.137 |
|  |  | 20-24 | 1.0 | 0.0000 | 0.00 | 0.044 | 0.137 |
|  |  | 25-29 | 1.1 | 0.0000 | 0.03 | 0.090 | 0.137 |
|  |  | 30-34 | 1.7 | 0.0000 | 0.13 | 0.113 | 0.137 |
|  |  | 35-39 | 2.4 | 0.0001 | 0.17 | 0.122 | 0.137 |
|  |  | 40-44 | 3.0 | 0.0009 | 0.16 | 0.147 | 0.137 |
|  |  | 45-49 | 3.6 | 0.0053 | 0.16 | 0.177 | 0.121 |
|  |  | 50-54 | 4.3 | 0.0113 | 0.19 | 0.200 | 0.121 |
|  |  | 55-59 | 5.2 | 0.0119 | 0.21 | 0.218 | 0.121 |
|  |  | 60-64 | 6.0 | 0.0079 | 0.27 | 0.224 | 0.121 |
|  |  | 65-69 | 6.7 | 0.0098 | 0.38 | 0.193 | 0.207 |
|  |  | 70-74 | 6.9 | 0.0109 | 0.43 | 0.161 | 0.207 |
|  |  | 75-79 | 6.4 | 0.0095 | 0.57 | 0.132 | 0.186 |
|  |  | 80-84 | 5.9 | 0.0072 | 0.86 | 0.078 | 0.186 |
|  |  | 85-89 | 6.2 | 0.0017 | 1.06 | 0.059 | 0.186 |
|  |  | 90-94 | 6.5 | 0.0001 | 1.16 | 0.056 | 0.186 |
|  |  | 95+ | 6.6 | 0.0001 | 1.29 | 0.052 | 0.186 |
| Female | non-Māori | 0-4 | 0.0 | 0.0000 | 0.00 | 0.000 | 0.229 |
|  |  | 5-9 | 0.0 | 0.0000 | 0.00 | 0.000 | 0.229 |
|  |  | 10-14 | 0.0 | 0.0000 | 0.00 | 0.000 | 0.229 |
|  |  | 15-19 | 0.9 | 0.0000 | 0.00 | 0.021 | 0.229 |
|  |  | 20-24 | 2.1 | 0.0000 | 0.00 | 0.102 | 0.229 |
|  |  | 25-29 | 2.5 | 0.0000 | 0.06 | 0.191 | 0.229 |
|  |  | 30-34 | 7.9 | 0.0000 | 0.26 | 0.255 | 0.229 |
|  |  | 35-39 | 11.0 | 0.0001 | 0.33 | 0.299 | 0.229 |
|  |  | 40-44 | 11.9 | 0.0007 | 0.30 | 0.353 | 0.229 |
|  |  | 45-49 | 12.5 | 0.0040 | 0.28 | 0.411 | 0.155 |
|  |  | 50-54 | 13.6 | 0.0078 | 0.28 | 0.449 | 0.155 |
|  |  | 55-59 | 14.7 | 0.0069 | 0.30 | 0.468 | 0.155 |
|  |  | 60-64 | 13.6 | 0.0047 | 0.27 | 0.493 | 0.155 |
|  |  | 65-69 | 12.8 | 0.0049 | 0.31 | 0.462 | 0.122 |
|  |  | 70-74 | 12.5 | 0.0051 | 0.32 | 0.402 | 0.122 |
|  |  | 75-79 | 10.0 | 0.0047 | 0.33 | 0.358 | 0.145 |
|  |  | 80-84 | 7.4 | 0.0075 | 0.46 | 0.222 | 0.145 |
|  |  | 85-89 | 9.7 | 0.0134 | 0.60 | 0.162 | 0.145 |
|  |  | 90-94 | 10.4 | 0.0156 | 0.65 | 0.157 | 0.145 |
|  |  | 95+ | 9.9 | 0.0156 | 0.69 | 0.143 | 0.145 |

Table S26: COPD input parameters

| **Sex** | **Ethnic group** | **Age group (years)** | **Incidence rate per 100,000** | **Case-fatality rate** | **Prevalence per 1000** | **Disability rate** |
| --- | --- | --- | --- | --- | --- | --- |
| Male | Māori | 0-4 | 0.0 | 0.0000 | 0.0 | 0.125 |
|  |  | 5-9 | 0.0 | 0.0000 | 0.0 | 0.125 |
|  |  | 10-14 | 0.0 | 0.0000 | 0.0 | 0.125 |
|  |  | 15-19 | 45.2 | 0.0000 | 2.9 | 0.125 |
|  |  | 20-24 | 74.1 | 0.0000 | 5.9 | 0.125 |
|  |  | 25-29 | 99.3 | 0.0002 | 10.3 | 0.125 |
|  |  | 30-34 | 107.1 | 0.0012 | 15.4 | 0.125 |
|  |  | 35-39 | 160.1 | 0.0020 | 21.7 | 0.125 |
|  |  | 40-44 | 206.7 | 0.0019 | 30.8 | 0.125 |
|  |  | 45-49 | 320.1 | 0.0015 | 43.1 | 0.083 |
|  |  | 50-54 | 528.7 | 0.0019 | 64.0 | 0.083 |
|  |  | 55-59 | 696.8 | 0.0038 | 93.8 | 0.091 |
|  |  | 60-64 | 832.2 | 0.0074 | 129.3 | 0.091 |
|  |  | 65-69 | 1008.7 | 0.0110 | 169.3 | 0.100 |
|  |  | 70-74 | 1157.4 | 0.0176 | 213.0 | 0.100 |
|  |  | 75-79 | 1196.9 | 0.0259 | 252.6 | 0.097 |
|  |  | 80-84 | 1164.2 | 0.0322 | 282.7 | 0.097 |
|  |  | 85-89 | 1136.9 | 0.0394 | 303.8 | 0.087 |
|  |  | 90-94 | 1054.3 | 0.0540 | 309.9 | 0.087 |
|  |  | 95+ | 960.7 | 0.0791 | 293.4 | 0.087 |
| Female | Māori | 0-4 | 0.0 | 0.0000 | 0.0 | 0.224 |
|  |  | 5-9 | 0.0 | 0.0000 | 0.0 | 0.224 |
|  |  | 10-14 | 0.0 | 0.0000 | 0.0 | 0.224 |
|  |  | 15-19 | 30.1 | 0.0000 | 2.2 | 0.224 |
|  |  | 20-24 | 51.3 | 0.0005 | 4.2 | 0.224 |
|  |  | 25-29 | 82.0 | 0.0015 | 7.5 | 0.224 |
|  |  | 30-34 | 123.4 | 0.0018 | 12.5 | 0.224 |
|  |  | 35-39 | 181.6 | 0.0016 | 19.9 | 0.224 |
|  |  | 40-44 | 249.7 | 0.0018 | 30.5 | 0.224 |
|  |  | 45-49 | 344.9 | 0.0022 | 44.8 | 0.160 |
|  |  | 50-54 | 458.4 | 0.0027 | 64.4 | 0.160 |
|  |  | 55-59 | 553.5 | 0.0053 | 88.4 | 0.167 |
|  |  | 60-64 | 664.4 | 0.0111 | 115.0 | 0.167 |
|  |  | 65-69 | 766.5 | 0.0159 | 143.2 | 0.170 |
|  |  | 70-74 | 839.4 | 0.0225 | 171.0 | 0.170 |
|  |  | 75-79 | 873.6 | 0.0290 | 194.2 | 0.149 |
|  |  | 80-84 | 868.7 | 0.0306 | 213.3 | 0.149 |
|  |  | 85-89 | 830.5 | 0.0289 | 230.1 | 0.113 |
|  |  | 90-94 | 765.5 | 0.0267 | 244.9 | 0.113 |
|  |  | 95+ | 690.0 | 0.0246 | 257.1 | 0.113 |
| Male | non-Māori | 0-4 | 0.0 | 0.0000 | 0.0 | 0.117 |
|  |  | 5-9 | 0.0 | 0.0000 | 0.0 | 0.117 |
|  |  | 10-14 | 0.0 | 0.0000 | 0.0 | 0.117 |
|  |  | 15-19 | 35.2 | 0.0000 | 1.7 | 0.117 |
|  |  | 20-24 | 66.1 | 0.0000 | 4.4 | 0.117 |
|  |  | 25-29 | 61.6 | 0.0000 | 7.7 | 0.117 |
|  |  | 30-34 | 49.2 | 0.0001 | 10.4 | 0.117 |
|  |  | 35-39 | 67.1 | 0.0002 | 13.2 | 0.117 |
|  |  | 40-44 | 84.2 | 0.0003 | 17.0 | 0.117 |
|  |  | 45-49 | 136.4 | 0.0005 | 22.2 | 0.083 |
|  |  | 50-54 | 235.5 | 0.0008 | 31.5 | 0.083 |
|  |  | 55-59 | 304.8 | 0.0018 | 44.9 | 0.084 |
|  |  | 60-64 | 380.3 | 0.0039 | 60.9 | 0.084 |
|  |  | 65-69 | 658.7 | 0.0063 | 84.3 | 0.096 |
|  |  | 70-74 | 906.8 | 0.0101 | 120.4 | 0.096 |
|  |  | 75-79 | 1030.2 | 0.0156 | 161.3 | 0.095 |
|  |  | 80-84 | 1054.2 | 0.0228 | 200.0 | 0.095 |
|  |  | 85-89 | 954.5 | 0.0333 | 227.1 | 0.095 |
|  |  | 90-94 | 847.6 | 0.0480 | 236.3 | 0.095 |
|  |  | 95+ | 755.9 | 0.0720 | 225.6 | 0.095 |
| Female | non-Māori | 0-4 | 0.0 | 0.0000 | 0.0 | 0.217 |
|  |  | 5-9 | 0.0 | 0.0000 | 0.0 | 0.217 |
|  |  | 10-14 | 0.0 | 0.0000 | 0.0 | 0.217 |
|  |  | 15-19 | 17.6 | 0.0000 | 1.2 | 0.217 |
|  |  | 20-24 | 33.2 | 0.0000 | 2.5 | 0.217 |
|  |  | 25-29 | 53.8 | 0.0000 | 4.6 | 0.217 |
|  |  | 30-34 | 81.6 | 0.0001 | 8.0 | 0.217 |
|  |  | 35-39 | 116.8 | 0.0002 | 12.9 | 0.217 |
|  |  | 40-44 | 159.9 | 0.0003 | 19.8 | 0.217 |
|  |  | 45-49 | 214.6 | 0.0005 | 29.0 | 0.119 |
|  |  | 50-54 | 277.7 | 0.0008 | 41.2 | 0.119 |
|  |  | 55-59 | 340.9 | 0.0018 | 56.4 | 0.116 |
|  |  | 60-64 | 408.8 | 0.0035 | 74.3 | 0.116 |
|  |  | 65-69 | 474.3 | 0.0054 | 94.7 | 0.122 |
|  |  | 70-74 | 526.3 | 0.0087 | 116.5 | 0.122 |
|  |  | 75-79 | 555.2 | 0.0132 | 137.6 | 0.127 |
|  |  | 80-84 | 558.3 | 0.0178 | 155.8 | 0.127 |
|  |  | 85-89 | 536.7 | 0.0238 | 169.3 | 0.117 |
|  |  | 90-94 | 496.1 | 0.0333 | 175.0 | 0.117 |
|  |  | 95+ | 450.2 | 0.0484 | 170.7 | 0.117 |

Table S27: LRTI input parameters

| **Sex** | **Ethnic group** | **Age group (years)** | **Mortality per 100,000** | **Disability rate** |
| --- | --- | --- | --- | --- |
| Male | Māori | 0-4 | 11.86 | 0.00064 |
|  |  | 5-9 | 0.00 | 0.00018 |
|  |  | 10-14 | 0.00 | 0.00019 |
|  |  | 15-19 | 0.00 | 0.00018 |
|  |  | 20-24 | 0.00 | 0.00024 |
|  |  | 25-29 | 0.00 | 0.00031 |
|  |  | 30-34 | 0.00 | 0.00023 |
|  |  | 35-39 | 0.00 | 0.00030 |
|  |  | 40-44 | 1.99 | 0.00028 |
|  |  | 45-49 | 0.00 | 0.00025 |
|  |  | 50-54 | 15.47 | 0.00035 |
|  |  | 55-59 | 6.63 | 0.00046 |
|  |  | 60-64 | 0.00 | 0.00045 |
|  |  | 65-69 | 0.00 | 0.00064 |
|  |  | 70-74 | 19.97 | 0.00077 |
|  |  | 75-79 | 74.44 | 0.00092 |
|  |  | 80-84 | 174.66 | 0.00116 |
|  |  | 85-89 | 312.50 | 0.00146 |
|  |  | 90-94 | 312.50 | 0.00146 |
|  |  | 95+ | 312.50 | 0.00146 |
| Female | Māori | 0-4 | 12.35 | 0.00059 |
|  |  | 5-9 | 0.00 | 0.00022 |
|  |  | 10-14 | 0.00 | 0.00008 |
|  |  | 15-19 | 0.00 | 0.00027 |
|  |  | 20-24 | 0.00 | 0.00030 |
|  |  | 25-29 | 0.00 | 0.00032 |
|  |  | 30-34 | 0.00 | 0.00029 |
|  |  | 35-39 | 0.00 | 0.00030 |
|  |  | 40-44 | 1.58 | 0.00031 |
|  |  | 45-49 | 1.80 | 0.00033 |
|  |  | 50-54 | 2.41 | 0.00044 |
|  |  | 55-59 | 0.00 | 0.00053 |
|  |  | 60-64 | 4.56 | 0.00060 |
|  |  | 65-69 | 0.00 | 0.00078 |
|  |  | 70-74 | 8.80 | 0.00063 |
|  |  | 75-79 | 83.68 | 0.00077 |
|  |  | 80-84 | 108.75 | 0.00083 |
|  |  | 85-89 | 542.45 | 0.00095 |
|  |  | 90-94 | 542.45 | 0.00095 |
|  |  | 95+ | 542.45 | 0.00095 |
| Male | non-Māori | 0-4 | 3.69 | 0.00032 |
|  |  | 5-9 | 0.30 | 0.00009 |
|  |  | 10-14 | 0.54 | 0.00009 |
|  |  | 15-19 | 0.00 | 0.00009 |
|  |  | 20-24 | 0.00 | 0.00012 |
|  |  | 25-29 | 0.00 | 0.00016 |
|  |  | 30-34 | 0.00 | 0.00012 |
|  |  | 35-39 | 0.29 | 0.00015 |
|  |  | 40-44 | 0.00 | 0.00014 |
|  |  | 45-49 | 0.25 | 0.00013 |
|  |  | 50-54 | 1.72 | 0.00018 |
|  |  | 55-59 | 2.02 | 0.00024 |
|  |  | 60-64 | 2.06 | 0.00024 |
|  |  | 65-69 | 4.28 | 0.00035 |
|  |  | 70-74 | 11.13 | 0.00042 |
|  |  | 75-79 | 26.31 | 0.00052 |
|  |  | 80-84 | 105.42 | 0.00066 |
|  |  | 85-89 | 474.57 | 0.00088 |
|  |  | 90-94 | 474.57 | 0.00088 |
|  |  | 95+ | 474.57 | 0.00088 |
| Female | non-Māori | 0-4 | 1.31 | 0.00026 |
|  |  | 5-9 | 0.00 | 0.00010 |
|  |  | 10-14 | 0.00 | 0.00004 |
|  |  | 15-19 | 0.00 | 0.00012 |
|  |  | 20-24 | 0.00 | 0.00013 |
|  |  | 25-29 | 0.00 | 0.00014 |
|  |  | 30-34 | 0.00 | 0.00013 |
|  |  | 35-39 | 0.00 | 0.00014 |
|  |  | 40-44 | 0.46 | 0.00014 |
|  |  | 45-49 | 0.24 | 0.00015 |
|  |  | 50-54 | 1.67 | 0.00020 |
|  |  | 55-59 | 2.09 | 0.00025 |
|  |  | 60-64 | 1.17 | 0.00028 |
|  |  | 65-69 | 3.68 | 0.00037 |
|  |  | 70-74 | 8.72 | 0.00030 |
|  |  | 75-79 | 20.07 | 0.00038 |
|  |  | 80-84 | 92.26 | 0.00040 |
|  |  | 85-89 | 558.75 | 0.00046 |
|  |  | 90-94 | 558.75 | 0.00046 |
|  |  | 95+ | 558.75 | 0.00046 |

Table S28: Future APC trends in incidence, CFR and remission

|  | **Incidence Trends** | | | | **Case-Fatality Trends** | | | | **Remission Trends** | | | |
| --- | --- | --- | --- | --- | --- | --- | --- | --- | --- | --- | --- | --- |
|  | **Non-Māori** | | **Māori** | | **Non-Māori** | | **Māori** | | **Non-Māori** | | **Māori** | |
|  | **Men** | **Woman** | **Men** | **Woman** | **Men** | **Woman** | **Men** | **Woman** | **Men** | **Woman** | **Men** | **Woman** |
| CHD | -2.00% | -2.00% | -2.00% | -2.00% | -2.00% | -2.00% | -2.00% | -2.00% |  |  |  |  |
| Stroke | -2.00% | -2.00% | -2.00% | -2.00% | -2.00% | -2.00% | -2.00% | -2.00% |  |  |  |  |
| COPD | -1.00% | -1.00% | -1.00% | -1.00% | -1.00% | -1.00% | -1.00% | -1.00% |  |  |  |  |
| LRTI |  |  |  |  |  |  |  |  |  |  |  |  |
| Bladder Cancer | 0.10% | 1.00% | 0.10% | 0.90% | 4.20% | 4.49% | 3.00% | 2.47% | -2.40% | -1.77% | -5.38% | -7.04% |
| Head & Neck Cancer | -1.80% | -0.30% | 0.80% | 2.40% | -1.26% | -1.55% | -1.28% | -1.33% | 1.93% | 1.13% | 1.87% | 1.72% |
| Esophageal Cancer | 0.10% | -0.60% | 0.10% | -0.70% | -0.32% | -0.23% | -0.29% | -0.14% | 1.98% | 2.47% | 2.12% | 3.18% |
| Liver Cancer | 1.90% | 1.60% | -0.10% | -0.30% | -0.71% | -0.86% | -0.77% | -0.76% | 4.30% | 3.64% | 4.04% | 4.06% |
| Cervical Cancer |  | -2.40% |  | -3.50% |  | -0.31% |  | -0.28% |  | 0.20% |  | 0.26% |
| Endometrial Cancer |  | 0.50% |  | 0.40% |  | -1.91% |  | -1.85% |  | 0.73% |  | 0.88% |
| Kidney Cancer | 1.30% | 1.50% | 1.30% | 1.50% | -1.83% | -1.83% | -1.64% | -1.56% | 0.93% | 0.93% | 1.39% | 1.61% |
| Leukemia | 1.90% | 1.50% | 1.80% | 1.40% | -3.50% | -2.51% | -2.90% | -1.76% | 1.37% | 4.04% | 2.91% | 6.75% |
| Lung Cancer | -3.80% | -0.40% | -2.90% | 0.50% | 0.02% | 0.02% | 0.02% | 0.02% | -0.24% | -0.24% | -0.29% | -0.25% |
| Melanoma | 1.90% | 1.60% | 1.90% | 1.60% |  |  |  |  |  |  |  |  |
| Pancreas Cancer | -1.80% | -1.40% | -1.80% | -1.40% | -0.03% | -0.02% | -0.02% | -0.01% | 0.23% | 0.30% | 0.28% | 0.33% |
| Stomach Cancer | -1.50% | -1.40% | -0.50% | -0.50% | -0.48% | -0.45% | -0.36% | -0.37% | 1.04% | 1.14% | 1.47% | 1.42% |
| Thyroid Cancer | 1.90% | 3.70% | 1.90% | 3.70% | -2.60% | -2.61% | -2.60% | -2.60% | 0.05% | 0.04% | 0.07% | 0.06% |

# Appendix D: Health system costs

This Appendix provides tables of the health system cost inputs into the BODE^3^ multistate life table models. This means that: all costs have been scaled up by 1.20 for assumed missing private expenditure; costs in the last year of life have been scaled up by 1.1 (for the 70-79 year old age group), 1.2 (for the80-89 year old age group) and 1.3 (for 90+ year old age group) to allow for estimated missing private hospital care that is likely consistent with a ‘health system perspective’ to costing; and (for disease-specific costs) they have been scaled down to allow for individuals with two or more diseases.

Table S29: Population health system costs

|  |  | **Population wide †** | | **Population without tobacco-related disease** | |
| --- | --- | --- | --- | --- | --- |
| **Sex** | **Age group** | **Not the last six months of life** | **Last six months of life** | **A** | **B** |
| Male | <1 | $5625 | $88115 | $5228 | $86376 |
|  | 1-4 | $1377 | $29742 | $1215 | $24727 |
|  | 5-9 | $695 | $27424 | $621 | $17653 |
|  | 10-14 | $665 | $27365 | $618 | $19944 |
|  | 15-19 | $714 | $15006 | $676 | $10959 |
|  | 20-24 | $750 | $9972 | $698 | $7851 |
|  | 25-29 | $771 | $14415 | $704 | $11225 |
|  | 30-34 | $848 | $16059 | $748 | $8926 |
|  | 35-39 | $920 | $15134 | $798 | $9052 |
|  | 40-44 | $1120 | $20921 | $886 | $10216 |
|  | 45-49 | $1370 | $22305 | $1015 | $11673 |
|  | 50-54 | $1754 | $23576 | $1175 | $11556 |
|  | 55-59 | $2206 | $25153 | $1336 | $12851 |
|  | 60-64 | $2798 | $23830 | $1612 | $12582 |
|  | 65-69 | $3803 | $24158 | $2145 | $14204 |
|  | 70-74 | $4752 | $23306 | $2685 | $13860 |
|  | 75-79 | $5483 | $20621 | $3164 | $12377 |
|  | 80-84 | $5927 | $18313 | $3583 | $11596 |
|  | 85-89 | $6275 | $15536 | $3978 | $10347 |
|  | 90-94 | $6059 | $13575 | $4247 | $8847 |
|  | 95-99 | $5576 | $11058 | $3874 | $6861 |

†Not actually used in the model, but used in calibration

**Table S30: Bladder cancer disease costs**

| **Sex** | **Age group** | **C** | **D** | **E** |
| --- | --- | --- | --- | --- |
| Male | <1 | $26795 | $1105 | $37346 |
|  | 1-4 | $26795 | $1105 | $37346 |
|  | 5-9 | $26795 | $1105 | $37346 |
|  | 10-14 | $26795 | $1105 | $37346 |
|  | 15-19 | $26795 | $1105 | $37346 |
|  | 20-24 | $26795 | $1105 | $37346 |
|  | 25-29 | $26795 | $1105 | $37346 |
|  | 30-34 | $26795 | $1105 | $37346 |
|  | 35-39 | $26795 | $1105 | $37346 |
|  | 40-44 | $26795 | $1105 | $37346 |
|  | 45-49 | $20169 | $2125 | $37346 |
|  | 50-54 | $20169 | $2125 | $37346 |
|  | 55-59 | $20169 | $2125 | $37346 |
|  | 60-64 | $20169 | $2125 | $37346 |
|  | 65-69 | $19638 | $2121 | $25864 |
|  | 70-74 | $19638 | $2121 | $28451 |
|  | 75-79 | $15088 | $2704 | $28451 |
|  | 80-84 | $15088 | $2704 | $31037 |
|  | 85-89 | $9052 | $3021 | $31037 |
|  | 90-94 | $9052 | $3021 | $33624 |
|  | 95-99 | $9052 | $3021 | $33624 |
| Females | <1 | $24673 | $4235 | $63441 |
|  | 1-4 | $24673 | $4235 | $63441 |
|  | 5-9 | $24673 | $4235 | $63441 |
|  | 10-14 | $24673 | $4235 | $63441 |
|  | 15-19 | $24673 | $4235 | $63441 |
|  | 20-24 | $24673 | $4235 | $63441 |
|  | 25-29 | $24673 | $4235 | $63441 |
|  | 30-34 | $24673 | $4235 | $63441 |
|  | 35-39 | $24673 | $4235 | $63441 |
|  | 40-44 | $24673 | $4235 | $63441 |
|  | 45-49 | $24673 | $1598 | $63441 |
|  | 50-54 | $24673 | $1598 | $63441 |
|  | 55-59 | $24673 | $1598 | $63441 |
|  | 60-64 | $24673 | $1598 | $63441 |
|  | 65-69 | $12832 | $1818 | $22437 |
|  | 70-74 | $12832 | $1818 | $24680 |
|  | 75-79 | $12832 | $2543 | $24680 |
|  | 80-84 | $12832 | $2543 | $26923 |
|  | 85-89 | $12832 | $1710 | $26923 |
|  | 90-94 | $12832 | $1710 | $29168 |
|  | 95-99 | $12832 | $1710 | $29168 |

**Table S31: COPD d**isease costs

| **Sex** | **Age group** | **C** | **D** | **E** |
| --- | --- | --- | --- | --- |
| Male | <1 | $11883 | $4278 | $25602 |
|  | 1-4 | $11883 | $4278 | $25602 |
|  | 5-9 | $11883 | $4278 | $25602 |
|  | 10-14 | $11883 | $4278 | $25602 |
|  | 15-19 | $11883 | $4278 | $25602 |
|  | 20-24 | $11883 | $4278 | $25602 |
|  | 25-29 | $11883 | $4278 | $25602 |
|  | 30-34 | $11883 | $4278 | $25602 |
|  | 35-39 | $11883 | $4278 | $25602 |
|  | 40-44 | $11883 | $4278 | $25602 |
|  | 45-49 | $11444 | $7485 | $25602 |
|  | 50-54 | $11444 | $7485 | $25602 |
|  | 55-59 | $11444 | $7485 | $25602 |
|  | 60-64 | $11444 | $7485 | $25602 |
|  | 65-69 | $8987 | $6311 | $14996 |
|  | 70-74 | $8987 | $6311 | $16497 |
|  | 75-79 | $8131 | $5008 | $16497 |
|  | 80-84 | $8131 | $5008 | $17996 |
|  | 85-89 | $6536 | $4666 | $17996 |
|  | 90-94 | $6536 | $4666 | $19496 |
|  | 95-99 | $6536 | $4666 | $19496 |
| Females | <1 | $9196 | $4387 | $23164 |
|  | 1-4 | $9196 | $4387 | $23164 |
|  | 5-9 | $9196 | $4387 | $23164 |
|  | 10-14 | $9196 | $4387 | $23164 |
|  | 15-19 | $9196 | $4387 | $23164 |
|  | 20-24 | $9196 | $4387 | $23164 |
|  | 25-29 | $9196 | $4387 | $23164 |
|  | 30-34 | $9196 | $4387 | $23164 |
|  | 35-39 | $9196 | $4387 | $23164 |
|  | 40-44 | $9196 | $4387 | $23164 |
|  | 45-49 | $9211 | $6278 | $23164 |
|  | 50-54 | $9211 | $6278 | $23164 |
|  | 55-59 | $9211 | $6278 | $23164 |
|  | 60-64 | $9211 | $6278 | $23164 |
|  | 65-69 | $8736 | $5906 | $13539 |
|  | 70-74 | $8736 | $5906 | $14893 |
|  | 75-79 | $7495 | $4727 | $14893 |
|  | 80-84 | $7495 | $4727 | $16247 |
|  | 85-89 | $5795 | $3292 | $16247 |
|  | 90-94 | $5795 | $3292 | $17601 |
|  | 95-99 | $5795 | $3292 | $17601 |

**Table S32: Cervical cancer disease costs**

| **Sex** | **Age group** | **C** | **D** | **E** |
| --- | --- | --- | --- | --- |
| Females | <1 | $19483 | $3034 | $35848 |
|  | 1-4 | $19483 | $3034 | $35848 |
|  | 5-9 | $19483 | $3034 | $35848 |
|  | 10-14 | $19483 | $3034 | $35848 |
|  | 15-19 | $19483 | $3034 | $35848 |
|  | 20-24 | $19483 | $3034 | $35848 |
|  | 25-29 | $19483 | $3034 | $35848 |
|  | 30-34 | $19483 | $3034 | $35848 |
|  | 35-39 | $19483 | $3034 | $35848 |
|  | 40-44 | $19483 | $3034 | $35848 |
|  | 45-49 | $18440 | $1795 | $28646 |
|  | 50-54 | $18440 | $1795 | $28646 |
|  | 55-59 | $18440 | $1795 | $28646 |
|  | 60-64 | $18440 | $1795 | $28646 |
|  | 65-69 | $20463 | $2919 | $32691 |
|  | 70-74 | $20463 | $2919 | $35961 |
|  | 75-79 | $25423 | $4646 | $15852 |
|  | 80-84 | $25423 | $4646 | $17293 |
|  | 85-89 | $19217 | $3329 | $17293 |
|  | 90-94 | $19217 | $3329 | $18734 |
|  | 95-99 | $19217 | $3329 | $18734 |

**Table S33: CHD d**isease costs

| **Sex** | **Age group** | **C** | **D** | **E** |
| --- | --- | --- | --- | --- |
| Male | <1 | $15451 | $4382 | $18902 |
|  | 1-4 | $15451 | $4382 | $18902 |
|  | 5-9 | $15451 | $4382 | $18902 |
|  | 10-14 | $15451 | $4382 | $18902 |
|  | 15-19 | $15451 | $4382 | $18902 |
|  | 20-24 | $15451 | $4382 | $18902 |
|  | 25-29 | $15451 | $4382 | $18902 |
|  | 30-34 | $15451 | $4382 | $18902 |
|  | 35-39 | $15451 | $4382 | $18902 |
|  | 40-44 | $15451 | $4382 | $18902 |
|  | 45-49 | $12805 | $2897 | $19287 |
|  | 50-54 | $12805 | $2897 | $19287 |
|  | 55-59 | $12805 | $2897 | $19287 |
|  | 60-64 | $12805 | $2897 | $19287 |
|  | 65-69 | $12580 | $2491 | $20778 |
|  | 70-74 | $12580 | $2491 | $22856 |
|  | 75-79 | $9959 | $2463 | $17525 |
|  | 80-84 | $9959 | $2463 | $19119 |
|  | 85-89 | $7476 | $3158 | $14954 |
|  | 90-94 | $7476 | $3158 | $16200 |
|  | 95-99 | $7476 | $3158 | $16200 |
| Females | <1 | $19291 | $6682 | $38349 |
|  | 1-4 | $19291 | $6682 | $38349 |
|  | 5-9 | $19291 | $6682 | $38349 |
|  | 10-14 | $19291 | $6682 | $38349 |
|  | 15-19 | $19291 | $6682 | $38349 |
|  | 20-24 | $19291 | $6682 | $38349 |
|  | 25-29 | $19291 | $6682 | $38349 |
|  | 30-34 | $19291 | $6682 | $38349 |
|  | 35-39 | $19291 | $6682 | $38349 |
|  | 40-44 | $19291 | $6682 | $38349 |
|  | 45-49 | $11484 | $4002 | $38349 |
|  | 50-54 | $11484 | $4002 | $38349 |
|  | 55-59 | $11484 | $4002 | $38349 |
|  | 60-64 | $11484 | $4002 | $38349 |
|  | 65-69 | $10923 | $3391 | $12326 |
|  | 70-74 | $10923 | $3391 | $13559 |
|  | 75-79 | $8494 | $2910 | $13559 |
|  | 80-84 | $8494 | $2910 | $14792 |
|  | 85-89 | $5297 | $2161 | $14792 |
|  | 90-94 | $5297 | $2161 | $16025 |
|  | 95-99 | $5297 | $2161 | $16025 |

**Table S34: Endometrial cancer d**isease costs

| **Sex** | **Age group** | **C** | **D** | **E** |
| --- | --- | --- | --- | --- |
| Females | <1 | $17559 | $2450 | $36119 |
|  | 1-4 | $17559 | $2450 | $36119 |
|  | 5-9 | $17559 | $2450 | $36119 |
|  | 10-14 | $17559 | $2450 | $36119 |
|  | 15-19 | $17559 | $2450 | $36119 |
|  | 20-24 | $17559 | $2450 | $36119 |
|  | 25-29 | $17559 | $2450 | $36119 |
|  | 30-34 | $17559 | $2450 | $36119 |
|  | 35-39 | $17559 | $2450 | $36119 |
|  | 40-44 | $17559 | $2450 | $36119 |
|  | 45-49 | $14017 | $1713 | $36119 |
|  | 50-54 | $14017 | $1713 | $36119 |
|  | 55-59 | $14017 | $1713 | $36119 |
|  | 60-64 | $14017 | $1713 | $36119 |
|  | 65-69 | $15079 | $1963 | $20133 |
|  | 70-74 | $15079 | $1963 | $22146 |
|  | 75-79 | $14459 | $1182 | $22146 |
|  | 80-84 | $14459 | $1182 | $24160 |
|  | 85-89 | $10507 | $1288 | $24160 |
|  | 90-94 | $10507 | $1288 | $26173 |
|  | 95-99 | $10507 | $1288 | $26173 |

**Table S35: Head and neck cancer d**isease costs

| **Sex** | **Age group** | **C** | **D** | **E** |
| --- | --- | --- | --- | --- |
| Male | <1 | $41022 | $4130 | $37526 |
|  | 1-4 | $41022 | $4130 | $37526 |
|  | 5-9 | $41022 | $4130 | $37526 |
|  | 10-14 | $41022 | $4130 | $37526 |
|  | 15-19 | $41022 | $4130 | $37526 |
|  | 20-24 | $41022 | $4130 | $37526 |
|  | 25-29 | $41022 | $4130 | $37526 |
|  | 30-34 | $41022 | $4130 | $37526 |
|  | 35-39 | $41022 | $4130 | $37526 |
|  | 40-44 | $41022 | $4130 | $37526 |
|  | 45-49 | $36416 | $2873 | $37526 |
|  | 50-54 | $36416 | $2873 | $37526 |
|  | 55-59 | $36416 | $2873 | $37526 |
|  | 60-64 | $36416 | $2873 | $37526 |
|  | 65-69 | $29772 | $3245 | $30910 |
|  | 70-74 | $29772 | $3245 | $34002 |
|  | 75-79 | $24181 | $2985 | $34002 |
|  | 80-84 | $24181 | $2985 | $37092 |
|  | 85-89 | $15746 | $6848 | $37092 |
|  | 90-94 | $15746 | $6848 | $40184 |
|  | 95-99 | $15746 | $6848 | $40184 |
| Females | <1 | $33369 | $2599 | $33354 |
|  | 1-4 | $33369 | $2599 | $33354 |
|  | 5-9 | $33369 | $2599 | $33354 |
|  | 10-14 | $33369 | $2599 | $33354 |
|  | 15-19 | $33369 | $2599 | $33354 |
|  | 20-24 | $33369 | $2599 | $33354 |
|  | 25-29 | $33369 | $2599 | $33354 |
|  | 30-34 | $33369 | $2599 | $33354 |
|  | 35-39 | $33369 | $2599 | $33354 |
|  | 40-44 | $33369 | $2599 | $33354 |
|  | 45-49 | $31061 | $2789 | $33354 |
|  | 50-54 | $31061 | $2789 | $33354 |
|  | 55-59 | $31061 | $2789 | $33354 |
|  | 60-64 | $31061 | $2789 | $33354 |
|  | 65-69 | $31058 | $7664 | $22461 |
|  | 70-74 | $31058 | $7664 | $24706 |
|  | 75-79 | $20507 | $3095 | $24706 |
|  | 80-84 | $20507 | $3095 | $26952 |
|  | 85-89 | $16145 | $2017 | $26952 |
|  | 90-94 | $16145 | $2017 | $29199 |
|  | 95-99 | $16145 | $2017 | $29199 |

**Table S36: Kidney cancer disease costs**

| **Sex** | **Age group** | **C** | **D** | **E** |
| --- | --- | --- | --- | --- |
| Male | <1 | $28784 | $3384 | $33065 |
|  | 1-4 | $28784 | $3384 | $33065 |
|  | 5-9 | $28784 | $3384 | $33065 |
|  | 10-14 | $28784 | $3384 | $33065 |
|  | 15-19 | $28784 | $3384 | $33065 |
|  | 20-24 | $28784 | $3384 | $33065 |
|  | 25-29 | $28784 | $3384 | $33065 |
|  | 30-34 | $28784 | $3384 | $33065 |
|  | 35-39 | $28784 | $3384 | $33065 |
|  | 40-44 | $28784 | $3384 | $33065 |
|  | 45-49 | $18289 | $4293 | $33065 |
|  | 50-54 | $18289 | $4293 | $33065 |
|  | 55-59 | $18289 | $4293 | $33065 |
|  | 60-64 | $18289 | $4293 | $33065 |
|  | 65-69 | $14527 | $4058 | $21217 |
|  | 70-74 | $14527 | $4058 | $23338 |
|  | 75-79 | $15362 | $3714 | $23338 |
|  | 80-84 | $15362 | $3714 | $25460 |
|  | 85-89 | $11455 | $2594 | $25460 |
|  | 90-94 | $11455 | $2594 | $27581 |
|  | 95-99 | $11455 | $2594 | $27581 |
| Females | <1 | $30663 | $3547 | $45180 |
|  | 1-4 | $30663 | $3547 | $45180 |
|  | 5-9 | $30663 | $3547 | $45180 |
|  | 10-14 | $30663 | $3547 | $45180 |
|  | 15-19 | $30663 | $3547 | $45180 |
|  | 20-24 | $30663 | $3547 | $45180 |
|  | 25-29 | $30663 | $3547 | $45180 |
|  | 30-34 | $30663 | $3547 | $45180 |
|  | 35-39 | $30663 | $3547 | $45180 |
|  | 40-44 | $30663 | $3547 | $45180 |
|  | 45-49 | $15265 | $3446 | $45180 |
|  | 50-54 | $15265 | $3446 | $45180 |
|  | 55-59 | $15265 | $3446 | $45180 |
|  | 60-64 | $15265 | $3446 | $45180 |
|  | 65-69 | $15749 | $3461 | $18862 |
|  | 70-74 | $15749 | $3461 | $20749 |
|  | 75-79 | $14221 | $2769 | $20749 |
|  | 80-84 | $14221 | $2769 | $22635 |
|  | 85-89 | $12749 | $2876 | $22635 |
|  | 90-94 | $12749 | $2876 | $24521 |
|  | 95-99 | $12749 | $2876 | $24521 |

**Table S37: Liver cancer d**isease costs

| **Sex** | **Age group** | **C** | **D** | **E** |
| --- | --- | --- | --- | --- |
| Male | <1 | $56890 | $15750 | $20256 |
|  | 1-4 | $56890 | $15750 | $20256 |
|  | 5-9 | $56890 | $15750 | $20256 |
|  | 10-14 | $56890 | $15750 | $20256 |
|  | 15-19 | $56890 | $15750 | $20256 |
|  | 20-24 | $56890 | $15750 | $20256 |
|  | 25-29 | $56890 | $15750 | $20256 |
|  | 30-34 | $56890 | $15750 | $20256 |
|  | 35-39 | $56890 | $15750 | $20256 |
|  | 40-44 | $56890 | $15750 | $20256 |
|  | 45-49 | $30471 | $10317 | $20256 |
|  | 50-54 | $30471 | $10317 | $20256 |
|  | 55-59 | $30471 | $10317 | $20256 |
|  | 60-64 | $30471 | $10317 | $20256 |
|  | 65-69 | $22112 | $7241 | $13136 |
|  | 70-74 | $22112 | $7241 | $14451 |
|  | 75-79 | $11893 | $3054 | $14451 |
|  | 80-84 | $11893 | $3054 | $15765 |
|  | 85-89 | $11893 | $3054 | $15765 |
|  | 90-94 | $11893 | $3054 | $17077 |
|  | 95-99 | $11893 | $3054 | $17077 |
| Females | <1 | $62918 | $3495 | $22974 |
|  | 1-4 | $62918 | $3495 | $22974 |
|  | 5-9 | $62918 | $3495 | $22974 |
|  | 10-14 | $62918 | $3495 | $22974 |
|  | 15-19 | $62918 | $3495 | $22974 |
|  | 20-24 | $62918 | $3495 | $22974 |
|  | 25-29 | $62918 | $3495 | $22974 |
|  | 30-34 | $62918 | $3495 | $22974 |
|  | 35-39 | $62918 | $3495 | $22974 |
|  | 40-44 | $62918 | $3495 | $22974 |
|  | 45-49 | $27522 | $6786 | $22974 |
|  | 50-54 | $27522 | $6786 | $22974 |
|  | 55-59 | $27522 | $6786 | $22974 |
|  | 60-64 | $27522 | $6786 | $22974 |
|  | 65-69 | $24583 | $9546 | $13552 |
|  | 70-74 | $24583 | $9546 | $14907 |
|  | 75-79 | $19230 | $3859 | $14907 |
|  | 80-84 | $19230 | $3859 | $16262 |
|  | 85-89 | $19230 | $3181 | $16262 |
|  | 90-94 | $19230 | $3181 | $17617 |
|  | 95-99 | $19230 | $3181 | $17617 |

**Table S38: Lung cancer d**isease costs

| **Sex** | **Age group** | **C** | **D** | **E** |
| --- | --- | --- | --- | --- |
| Male | <1 | $36736 | $2888 | $31826 |
|  | 1-4 | $36736 | $2888 | $31826 |
|  | 5-9 | $36736 | $2888 | $31826 |
|  | 10-14 | $36736 | $2888 | $31826 |
|  | 15-19 | $36736 | $2888 | $31826 |
|  | 20-24 | $36736 | $2888 | $31826 |
|  | 25-29 | $36736 | $2888 | $31826 |
|  | 30-34 | $36736 | $2888 | $31826 |
|  | 35-39 | $36736 | $2888 | $31826 |
|  | 40-44 | $36736 | $2888 | $31826 |
|  | 45-49 | $28065 | $4382 | $31826 |
|  | 50-54 | $28065 | $4382 | $31826 |
|  | 55-59 | $28065 | $4382 | $31826 |
|  | 60-64 | $28065 | $4382 | $31826 |
|  | 65-69 | $23645 | $5302 | $16390 |
|  | 70-74 | $23645 | $5302 | $18029 |
|  | 75-79 | $15400 | $3919 | $18029 |
|  | 80-84 | $15400 | $3919 | $19668 |
|  | 85-89 | $9886 | $2801 | $19668 |
|  | 90-94 | $9886 | $2801 | $21306 |
|  | 95-99 | $9886 | $2801 | $21306 |
| Females | <1 | $62918 | $9580 | $22974 |
|  | 1-4 | $62918 | $9580 | $22974 |
|  | 5-9 | $62918 | $9580 | $22974 |
|  | 10-14 | $62918 | $9580 | $22974 |
|  | 15-19 | $62918 | $9580 | $22974 |
|  | 20-24 | $62918 | $9580 | $22974 |
|  | 25-29 | $62918 | $9580 | $22974 |
|  | 30-34 | $62918 | $9580 | $22974 |
|  | 35-39 | $62918 | $9580 | $22974 |
|  | 40-44 | $62918 | $9580 | $22974 |
|  | 45-49 | $27522 | $7077 | $22974 |
|  | 50-54 | $27522 | $7077 | $22974 |
|  | 55-59 | $27522 | $7077 | $22974 |
|  | 60-64 | $27522 | $7077 | $22974 |
|  | 65-69 | $24583 | $5315 | $13552 |
|  | 70-74 | $24583 | $5315 | $14907 |
|  | 75-79 | $19230 | $5256 | $14907 |
|  | 80-84 | $19230 | $5256 | $16262 |
|  | 85-89 | $19230 | $5256 | $16262 |
|  | 90-94 | $19230 | $5256 | $17617 |
|  | 95-99 | $19230 | $5256 | $17617 |

**Table S39: Melanoma d**isease costs

| **Sex** | **Age group** | **C** | **D** | **E** |
| --- | --- | --- | --- | --- |
| Male | <1 | $3901 | $406 | $33930 |
|  | 1-4 | $3901 | $406 | $33930 |
|  | 5-9 | $3901 | $406 | $33930 |
|  | 10-14 | $3901 | $406 | $33930 |
|  | 15-19 | $3901 | $406 | $33930 |
|  | 20-24 | $3901 | $406 | $33930 |
|  | 25-29 | $3901 | $406 | $33930 |
|  | 30-34 | $3901 | $406 | $33930 |
|  | 35-39 | $3901 | $406 | $33930 |
|  | 40-44 | $3901 | $406 | $33930 |
|  | 45-49 | $2325 | $285 | $27792 |
|  | 50-54 | $2325 | $285 | $27792 |
|  | 55-59 | $2325 | $285 | $27792 |
|  | 60-64 | $2325 | $285 | $27792 |
|  | 65-69 | $2917 | $1188 | $18533 |
|  | 70-74 | $2917 | $1188 | $20386 |
|  | 75-79 | $4236 | $1796 | $17749 |
|  | 80-84 | $4236 | $1796 | $19362 |
|  | 85-89 | $4300 | $2902 | $23158 |
|  | 90-94 | $4300 | $2902 | $25088 |
|  | 95-99 | $4300 | $2902 | $25088 |
| Females | <1 | $2297 | $498 | $35721 |
|  | 1-4 | $2297 | $498 | $35721 |
|  | 5-9 | $2297 | $498 | $35721 |
|  | 10-14 | $2297 | $498 | $35721 |
|  | 15-19 | $2297 | $498 | $35721 |
|  | 20-24 | $2297 | $498 | $35721 |
|  | 25-29 | $2297 | $498 | $35721 |
|  | 30-34 | $2297 | $498 | $35721 |
|  | 35-39 | $2297 | $498 | $35721 |
|  | 40-44 | $2297 | $498 | $35721 |
|  | 45-49 | $1834 | $39 | $21046 |
|  | 50-54 | $1834 | $39 | $21046 |
|  | 55-59 | $1834 | $39 | $21046 |
|  | 60-64 | $1834 | $39 | $21046 |
|  | 65-69 | $2773 | $723 | $20701 |
|  | 70-74 | $2773 | $723 | $22772 |
|  | 75-79 | $4458 | $1370 | $17989 |
|  | 80-84 | $4458 | $1370 | $19624 |
|  | 85-89 | $3363 | $1419 | $12391 |
|  | 90-94 | $3363 | $1419 | $13424 |
|  | 95-99 | $3363 | $1419 | $13424 |

**Table S40: Esophageal cancer d**isease costs

| **Sex** | **Age group** | **C** | **D** | **E** |
| --- | --- | --- | --- | --- |
| Male | <1 | $41098 | $19284 | $35864 |
|  | 1-4 | $41098 | $19284 | $35864 |
|  | 5-9 | $41098 | $19284 | $35864 |
|  | 10-14 | $41098 | $19284 | $35864 |
|  | 15-19 | $41098 | $19284 | $35864 |
|  | 20-24 | $41098 | $19284 | $35864 |
|  | 25-29 | $41098 | $19284 | $35864 |
|  | 30-34 | $41098 | $19284 | $35864 |
|  | 35-39 | $41098 | $19284 | $35864 |
|  | 40-44 | $41098 | $19284 | $35864 |
|  | 45-49 | $41098 | $5876 | $35864 |
|  | 50-54 | $41098 | $5876 | $35864 |
|  | 55-59 | $41098 | $5876 | $35864 |
|  | 60-64 | $41098 | $5876 | $35864 |
|  | 65-69 | $28524 | $3614 | $21550 |
|  | 70-74 | $28524 | $3614 | $23706 |
|  | 75-79 | $28524 | $13381 | $23706 |
|  | 80-84 | $28524 | $13381 | $25861 |
|  | 85-89 | $28524 | $13381 | $25861 |
|  | 90-94 | $28524 | $13381 | $28016 |
|  | 95-99 | $28524 | $13381 | $28016 |
| Females | <1 | $38856 | $5236 | $35580 |
|  | 1-4 | $38856 | $5236 | $35580 |
|  | 5-9 | $38856 | $5236 | $35580 |
|  | 10-14 | $38856 | $5236 | $35580 |
|  | 15-19 | $38856 | $5236 | $35580 |
|  | 20-24 | $38856 | $5236 | $35580 |
|  | 25-29 | $38856 | $5236 | $35580 |
|  | 30-34 | $38856 | $5236 | $35580 |
|  | 35-39 | $38856 | $5236 | $35580 |
|  | 40-44 | $38856 | $5236 | $35580 |
|  | 45-49 | $38856 | $5236 | $35580 |
|  | 50-54 | $38856 | $5236 | $35580 |
|  | 55-59 | $38856 | $5236 | $35580 |
|  | 60-64 | $38856 | $5236 | $35580 |
|  | 65-69 | $22377 | $3431 | $19857 |
|  | 70-74 | $22377 | $3431 | $21844 |
|  | 75-79 | $22377 | $3431 | $21844 |
|  | 80-84 | $22377 | $3431 | $23829 |
|  | 85-89 | $22377 | $3431 | $23829 |
|  | 90-94 | $22377 | $3431 | $25815 |
|  | 95-99 | $22377 | $3431 | $25815 |

**Table S41: Pancreatic cancer d**isease costs

| **Sex** | **Age group** | **C** | **D** | **E** |
| --- | --- | --- | --- | --- |
| Male | <1 | $66801 | $16141 | $30263 |
|  | 1-4 | $66801 | $16141 | $30263 |
|  | 5-9 | $66801 | $16141 | $30263 |
|  | 10-14 | $66801 | $16141 | $30263 |
|  | 15-19 | $66801 | $16141 | $30263 |
|  | 20-24 | $66801 | $16141 | $30263 |
|  | 25-29 | $66801 | $16141 | $30263 |
|  | 30-34 | $66801 | $16141 | $30263 |
|  | 35-39 | $66801 | $16141 | $30263 |
|  | 40-44 | $66801 | $16141 | $30263 |
|  | 45-49 | $42276 | $10327 | $30263 |
|  | 50-54 | $42276 | $10327 | $30263 |
|  | 55-59 | $42276 | $10327 | $30263 |
|  | 60-64 | $42276 | $10327 | $30263 |
|  | 65-69 | $28697 | $6162 | $15957 |
|  | 70-74 | $28697 | $6162 | $17553 |
|  | 75-79 | $19597 | $2505 | $17553 |
|  | 80-84 | $19597 | $2505 | $19149 |
|  | 85-89 | $19597 | $2505 | $19149 |
|  | 90-94 | $19597 | $2505 | $20745 |
|  | 95-99 | $19597 | $2505 | $20745 |
| Females | <1 | $37685 | $13777 | $29290 |
|  | 1-4 | $37685 | $13777 | $29290 |
|  | 5-9 | $37685 | $13777 | $29290 |
|  | 10-14 | $37685 | $13777 | $29290 |
|  | 15-19 | $37685 | $13777 | $29290 |
|  | 20-24 | $37685 | $13777 | $29290 |
|  | 25-29 | $37685 | $13777 | $29290 |
|  | 30-34 | $37685 | $13777 | $29290 |
|  | 35-39 | $37685 | $13777 | $29290 |
|  | 40-44 | $37685 | $13777 | $29290 |
|  | 45-49 | $37030 | $3166 | $29290 |
|  | 50-54 | $37030 | $3166 | $29290 |
|  | 55-59 | $37030 | $3166 | $29290 |
|  | 60-64 | $37030 | $3166 | $29290 |
|  | 65-69 | $32521 | $4616 | $14386 |
|  | 70-74 | $32521 | $4616 | $15825 |
|  | 75-79 | $19643 | $3396 | $15825 |
|  | 80-84 | $19643 | $3396 | $17264 |
|  | 85-89 | $19643 | $937 | $17264 |
|  | 90-94 | $19643 | $937 | $18702 |
|  | 95-99 | $19643 | $937 | $18702 |

**Table S42: Stomach cancer d**isease costs

| **Sex** | **Age group** | **C** | **D** | **E** |
| --- | --- | --- | --- | --- |
| Male | <1 | $48329 | $1931 | $51193 |
|  | 1-4 | $48329 | $1931 | $51193 |
|  | 5-9 | $48329 | $1931 | $51193 |
|  | 10-14 | $48329 | $1931 | $51193 |
|  | 15-19 | $48329 | $1931 | $51193 |
|  | 20-24 | $48329 | $1931 | $51193 |
|  | 25-29 | $48329 | $1931 | $51193 |
|  | 30-34 | $48329 | $1931 | $51193 |
|  | 35-39 | $48329 | $1931 | $51193 |
|  | 40-44 | $48329 | $1931 | $51193 |
|  | 45-49 | $44341 | $8272 | $24819 |
|  | 50-54 | $44341 | $8272 | $24819 |
|  | 55-59 | $44341 | $8272 | $24819 |
|  | 60-64 | $44341 | $8272 | $24819 |
|  | 65-69 | $32594 | $4295 | $24984 |
|  | 70-74 | $32594 | $4295 | $27482 |
|  | 75-79 | $21968 | $2159 | $22735 |
|  | 80-84 | $21968 | $2159 | $24803 |
|  | 85-89 | $11608 | $2384 | $14452 |
|  | 90-94 | $11608 | $2384 | $15657 |
|  | 95-99 | $11608 | $2384 | $15657 |
| Females | <1 | $60299 | $3495 | $51756 |
|  | 1-4 | $60299 | $3495 | $51756 |
|  | 5-9 | $60299 | $3495 | $51756 |
|  | 10-14 | $60299 | $3495 | $51756 |
|  | 15-19 | $60299 | $3495 | $51756 |
|  | 20-24 | $60299 | $3495 | $51756 |
|  | 25-29 | $60299 | $3495 | $51756 |
|  | 30-34 | $60299 | $3495 | $51756 |
|  | 35-39 | $60299 | $3495 | $51756 |
|  | 40-44 | $60299 | $3495 | $51756 |
|  | 45-49 | $38469 | $6786 | $27114 |
|  | 50-54 | $38469 | $6786 | $27114 |
|  | 55-59 | $38469 | $6786 | $27114 |
|  | 60-64 | $38469 | $6786 | $27114 |
|  | 65-69 | $34030 | $9546 | $18842 |
|  | 70-74 | $34030 | $9546 | $20726 |
|  | 75-79 | $22881 | $3859 | $20952 |
|  | 80-84 | $22881 | $3859 | $22856 |
|  | 85-89 | $10378 | $3181 | $13422 |
|  | 90-94 | $10378 | $3181 | $14541 |
|  | 95-99 | $10378 | $3181 | $14541 |

**Table S43: Thyroid cancer d**isease costs

| **Sex** | **Age group** | **C** | **D** | **E** |
| --- | --- | --- | --- | --- |
| Male | <1 | $12799 | $2358 | $31211 |
|  | 1-4 | $12799 | $2358 | $31211 |
|  | 5-9 | $12799 | $2358 | $31211 |
|  | 10-14 | $12799 | $2358 | $31211 |
|  | 15-19 | $12799 | $2358 | $31211 |
|  | 20-24 | $12799 | $2358 | $31211 |
|  | 25-29 | $12799 | $2358 | $31211 |
|  | 30-34 | $12799 | $2358 | $31211 |
|  | 35-39 | $12799 | $2358 | $31211 |
|  | 40-44 | $12799 | $2358 | $31211 |
|  | 45-49 | $16962 | $4233 | $31211 |
|  | 50-54 | $16962 | $4233 | $31211 |
|  | 55-59 | $16962 | $4233 | $31211 |
|  | 60-64 | $16962 | $4233 | $31211 |
|  | 65-69 | $17110 | $3787 | $31211 |
|  | 70-74 | $17110 | $3787 | $34333 |
|  | 75-79 | $9909 | $2814 | $34333 |
|  | 80-84 | $9909 | $2814 | $37454 |
|  | 85-89 | $9909 | $2422 | $37454 |
|  | 90-94 | $9909 | $2422 | $40576 |
|  | 95-99 | $9909 | $2422 | $40576 |
| Females | <1 | $11700 | $1323 | $36176 |
|  | 1-4 | $11700 | $1323 | $36176 |
|  | 5-9 | $11700 | $1323 | $36176 |
|  | 10-14 | $11700 | $1323 | $36176 |
|  | 15-19 | $11700 | $1323 | $36176 |
|  | 20-24 | $11700 | $1323 | $36176 |
|  | 25-29 | $11700 | $1323 | $36176 |
|  | 30-34 | $11700 | $1323 | $36176 |
|  | 35-39 | $11700 | $1323 | $36176 |
|  | 40-44 | $11700 | $1323 | $36176 |
|  | 45-49 | $10337 | $1779 | $36176 |
|  | 50-54 | $10337 | $1779 | $36176 |
|  | 55-59 | $10337 | $1779 | $36176 |
|  | 60-64 | $10337 | $1779 | $36176 |
|  | 65-69 | $18969 | $3481 | $36176 |
|  | 70-74 | $18969 | $3481 | $39794 |
|  | 75-79 | $9463 | $2198 | $39794 |
|  | 80-84 | $9463 | $2198 | $43412 |
|  | 85-89 | $9463 | $1437 | $43412 |
|  | 90-94 | $9463 | $1437 | $47030 |
|  | 95-99 | $9463 | $1437 | $47030 |

**Table S44: Stroke d**isease costs

| **Sex** | **Age group** | **C** | **D** | **E** |
| --- | --- | --- | --- | --- |
| Male | <1 | $15077 | $3633 | $11288 |
|  | 1-4 | $15077 | $3633 | $11288 |
|  | 5-9 | $15077 | $3633 | $11288 |
|  | 10-14 | $15077 | $3633 | $11288 |
|  | 15-19 | $15077 | $3633 | $11288 |
|  | 20-24 | $15077 | $3633 | $11288 |
|  | 25-29 | $15077 | $3633 | $11288 |
|  | 30-34 | $15077 | $3633 | $11288 |
|  | 35-39 | $15077 | $3633 | $11288 |
|  | 40-44 | $15077 | $3633 | $11288 |
|  | 45-49 | $8733 | $3567 | $11288 |
|  | 50-54 | $8733 | $3567 | $11288 |
|  | 55-59 | $8733 | $3567 | $11288 |
|  | 60-64 | $8733 | $3567 | $11288 |
|  | 65-69 | $6848 | $2724 | $6588 |
|  | 70-74 | $6848 | $2724 | $7247 |
|  | 75-79 | $5203 | $2479 | $7247 |
|  | 80-84 | $5203 | $2479 | $7906 |
|  | 85-89 | $5367 | $2813 | $7906 |
|  | 90-94 | $5367 | $2813 | $8565 |
|  | 95-99 | $5367 | $2813 | $8565 |
| Females | <1 | $14833 | $4382 | $14585 |
|  | 1-4 | $14833 | $4382 | $14585 |
|  | 5-9 | $14833 | $4382 | $14585 |
|  | 10-14 | $14833 | $4382 | $14585 |
|  | 15-19 | $14833 | $4382 | $14585 |
|  | 20-24 | $14833 | $4382 | $14585 |
|  | 25-29 | $14833 | $4382 | $14585 |
|  | 30-34 | $14833 | $4382 | $14585 |
|  | 35-39 | $14833 | $4382 | $14585 |
|  | 40-44 | $14833 | $4382 | $14585 |
|  | 45-49 | $9959 | $3159 | $14585 |
|  | 50-54 | $9959 | $3159 | $14585 |
|  | 55-59 | $9959 | $3159 | $14585 |
|  | 60-64 | $9959 | $3159 | $14585 |
|  | 65-69 | $7203 | $3165 | $4883 |
|  | 70-74 | $7203 | $3165 | $5372 |
|  | 75-79 | $5367 | $2369 | $5372 |
|  | 80-84 | $5367 | $2369 | $5860 |
|  | 85-89 | $4054 | $1548 | $5860 |
|  | 90-94 | $4054 | $1548 | $6348 |
|  | 95-99 | $4054 | $1548 | $6348 |
